# Hexa-Fe(III) Carboxylate Complexes Facilitate Aerobic Hydrocarbon Oxidative Functionalization: Rh Catalyzed Oxidative Coupling of Benzene and Ethylene to Form Styrene

**DOI:** 10.1021/acscatal.4c02355

**Published:** 2024-06-24

**Authors:** Marc T. Bennett, Kwanwoo A. Park, Charles B. Musgrave, Jack W. Brubaker, Diane A. Dickie, William A. Goddard, T. Brent Gunnoe

**Affiliations:** † Department of Chemistry, University of Virginia, Charlottesville, Virginia 22904, United States; ‡ Materials and Process Simulation Center, California Institute of Technology, Pasadena, California 91125, United States

**Keywords:** C−H activation, styrene, oxidative arene alkenylation, rhodium, iron

## Abstract

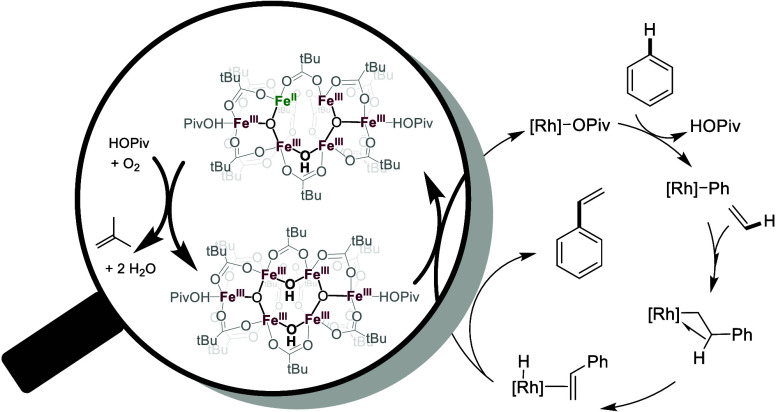

Fe(II) carboxylates react with dioxygen and carboxylic acid to form Fe_6_(μ–OH)_2_(μ_3_–O)_2_(μ–X)_12_(HX)_2_ (X = acetate or pivalate), which is an active oxidant for Rh-catalyzed arene alkenylation. Heating (150–200 °C) the catalyst precursor [(η^2^–C_2_H_4_)_2_Rh(μ–OAc)]_2_ with ethylene, benzene, Fe(II) carboxylate, and dioxygen yields styrene >30-fold faster than the reaction with dioxygen in the absence of the Fe(II) carboxylate additive. It is also demonstrated that Fe_6_(μ–OH)_2_(μ_3_–O)_2_(μ–X)_12_(HX)_2_ is an active oxidant under anaerobic conditions, and the reduced material can be reoxidized to Fe_6_(μ–OH)_2_(μ_3_–O)_2_(μ–X)_12_(HX)_2_ by dioxygen. At optimized conditions, a turnover frequency of ∼0.2 s^–1^ is achieved. Unlike analogous reactions with Cu(II) carboxylate oxidants, which undergo stoichiometric Cu(II)-mediated production of phenyl esters (e.g., phenyl acetate) as side products at temperatures ≥150 °C, no phenyl ester side product is observed when Fe carboxylate additives are used. Kinetic isotope effect experiments using C_6_H_6_ and C_6_D_6_ give *k*_H_/*k*_D_ = 3.5(3), while the use of protio or monodeutero pivalic acid reveals a small KIE with *k*_H_/*k*_D_ = 1.19(2). First-order dependencies on Fe(II) carboxylate and dioxygen concentration are observed in addition to complicated kinetic dependencies on the concentration of carboxylic acid and ethylene, both of which inhibit the reaction rate at a high concentration. Mechanistic studies are consistent with irreversible benzene C–H activation, ethylene insertion into the formed Rh–Ph bond, β–hydride elimination, and reaction of Rh–H with Fe_6_(μ–OH)_2_(μ_3_–O)_2_(μ–X)_12_(HX)_2_ to regenerate a Rh-carboxylate complex.

## Introduction

For many catalytic oxidation reactions, the use of dioxygen as the terminal oxidant can be essential for potential scalability.^1^ Transition-metal oxidative functionalization of hydrocarbons can involve β-hydride elimination reactions to produce metal hydride intermediates, and the reaction of metal hydride intermediates with an oxidant for the net removal of hydrogen is often critical to a successful catalytic cycle.^2,3^ Direct reaction of dioxygen with metal hydride intermediates to regenerate an active catalyst is often kinetically challenging and/or unselective.^4^ An alternative to the direct reaction of dioxygen with metal hydride intermediates is the use of a co-oxidant, which upon reduction can be reoxidized by dioxygen.^5,6^ A classic example of this latter approach is the Pd-catalyzed Hoechst–Wacker process for ethylene oxidation for which CuCl_2_ reacts with a Pd hydride intermediate, and CuCl and HCl are converted back to CuCl_2_ upon reaction with dioxygen.^7,8^

There are limited examples of co-oxidants capable of rapid reaction with metal hydrides that also have oxidation potentials amenable to aerobic reoxidation.^6,9^ There are a few reports of Fe-based oxidants for transition-metal catalyzed processes. For example, Pd-catalyzed Wacker oxidation has been reported using simple Fe salts in addition to Fe(II) phthalocyanine under aerobic conditions in the presence of 1,4-benzoquinone.^5,10,11^ Also, the Bäckvall group has reported aerobic Pd-catalyzed arene–olefin and olefin–olefin oxidative coupling reactions using Fe(II) phthalocyanine additive, which was proposed to react with dioxygen to form a species capable of reoxidizing hydroquinone to 1,4-benzoquinone, the proposed direct oxidant.^12−14^

Given the widespread use of Cu(II) and Ag(I) carboxylate oxidants for catalytic oxidative coupling reactions that operate through C–H activation, we hypothesized that Fe carboxylates might provide similar activity as oxidants.^2,3,15,16^ In addition to the potential use of Fe(III) carboxylates, formulated as FeX_3_ (X = carboxylate)^17^ or trinuclear Fe carboxylates formulated as [Fe(μ_3_–O)(μ–X)_6_]X^18,19^ as an oxidant, previous reports disclose that multinuclear Fe(II) carboxylate complexes can undergo reaction with dioxygen to form superoxo, peroxo, μ-hydroxide, or μ-oxo complexes, which could serve as active oxidants.^20−25^ Also relevant are multinuclear Fe carboxylate complexes containing μ_4_-peroxo^26−29^ and μ-hydroxide^18,28,30−33^ functionalities. Previously, Fe carboxylate complexes have been reported as catalyst precursors for thermal^20,26,28,33,34^ and photodriven^35^ hydrocarbon oxidation reactions.

**Scheme 1:**
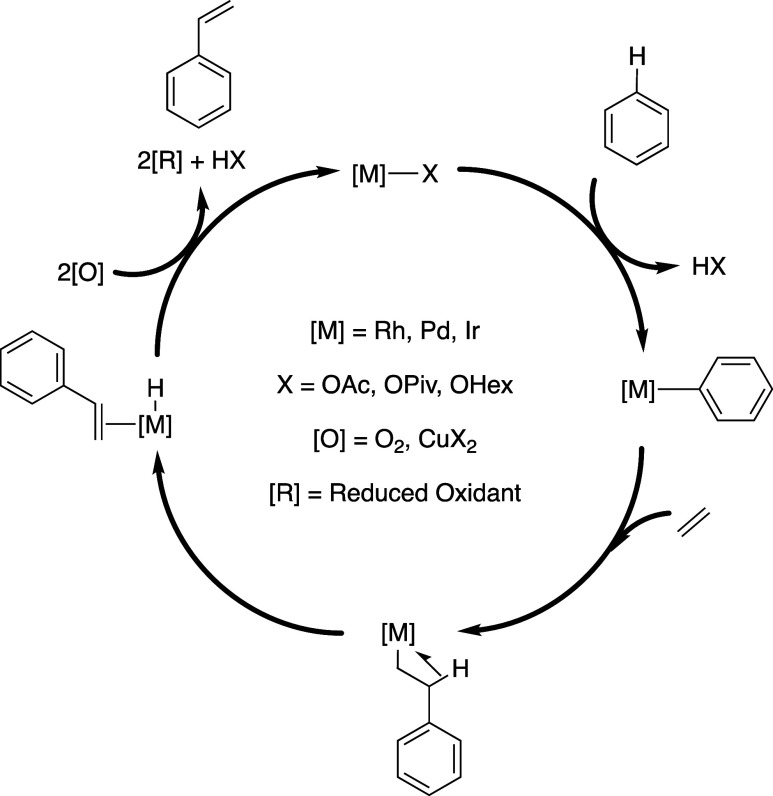
General Mechanism of Transition-Metal Catalyzed Arene Alkenylation

Our group in collaboration with the Goddard and Cundari groups in addition to the Goldberg, Hartwig/Eisenstein, Periana/Goddard, Tilley, and Milstein groups have studied molecular Ni,^36,37^ Ru,^38−44^ Pt,^45−53^ and Ir^54−57^ catalysts for arene alkylation and Rh,^58−63^ Ru,^64,65^ and Pd^66,67^ catalysts for arene alkenylation. These catalytic processes are attractive routes to synthesize alkyl and alkenyl arenes, which are used as precursors to polymers, fragrances, detergents, and pharmaceuticals.^68^ The catalytic oxidative arene alkenylation processes generally involve the combination of arene and olefin with an oxidant at temperatures ≥120 °C (Scheme 1). The proposed reaction mechanisms generally involve arene C–H activation, olefin insertion, β–hydride elimination, and reaction of the resulting metal–hydride intermediate with an oxidant to regenerate the starting catalyst. Previously reported oxidants for oxidative arene alkenylation include dioxygen,^59,65^ peroxides,^69,70^ ethylene,^64^ Cu(II) salts,^71−73^ and Ag(I) salts.^74^

Our group has studied Rh^58,60−63,75−77^ and Pd^66,67^ catalysis for arene alkenylation utilizing Cu(II) carboxylates as the oxidant both in the absence and presence of dioxygen. Following the reduction of Cu(II) to Cu(I) and formation of carboxylic acid, dioxygen can be used to regenerate Cu(II) carboxylate either *in situ* or in a separate step from catalysis at lower temperatures.^77^ Rh-catalyzed arene alkenylation can also be performed with dioxygen as the direct oxidant;^59^ however, the turnover frequency is 2 orders of magnitude slower than when Cu(II) carboxylates are present. Additionally, with dioxygen as the sole oxidant, the oxidation of the styrene product to benzaldehyde by dioxygen is kinetically competitive with styrene formation.

An important disadvantage of using Cu(II) carboxylates as the oxidant is that they mediate a stoichiometric side reaction with arenes to form aryl esters (e.g., phenyl acetate).^61,77,78^ The formation of aryl esters is kinetically competitive with alkenyl arene production at temperatures >170 °C, and irreversible thermal decomposition of Cu(II) carboxylates is typically observed at temperatures >180 °C.^79^

With the goals of (1) avoiding phenyl ester and other side product production and (2) achieving comparable or faster turnover frequencies to those measured with Cu(II) carboxylate oxidants, we sought out an alternative additive to Cu(II) carboxylates. We hypothesized that Fe carboxylates might be suitable additives given the potential dioxygen recyclability of Fe(II) and the potential ability of Fe(III) complexes to serve as an oxidant (see above).

Herein, we report that Fe(II) carboxylates react with dioxygen to form an active oxidant for styrene production from benzene and ethylene, with [(η^2^–C_2_H_4_)_2_Rh(μ–OAc)]_2_ as the catalyst precursor. Mechanistic studies indicate first-order dependencies on dioxygen and Fe(II) carboxylate concentration with complicated dependencies on ethylene and carboxylic acid concentrations. The proposed active oxidant is likely Fe_6_(μ–OH)_2_(μ_3_–O)_2_(μ–X)_12_(HX)_2_ (X = carboxylate). Further, this catalytic process reduces the formation of phenyl ester byproducts compared to the use of Cu(II) carboxylates as an *in situ* oxidant.

## Results and Discussion

### Catalyst Activity with Fe(II) and Fe(III) Carboxylate Additives

Rh-catalyzed benzene ethenylation was performed with [(η^2^–C_2_H_4_)_2_Rh(μ–OAc)]_2_ (OAc = acetate) as the catalyst precursor in the presence of 0.960 mol % HOPiv (OPiv = pivalate) relative to benzene with 70 psig of ethylene. Three formulations of Fe carboxylate additives were identified and probed, including Fe(OAc)_2_, which could form a higher oxidation state species in the presence of dioxygen,^20,21,25^ Fe(TFA)_3_,^17^ and the μ_3_-oxo-centered trinuclear complex [Fe(μ_3_–O)(μ–OPiv)_6_][OPiv].^18^ Additional Fe(II) and Fe(III) salts not containing carboxylate ligands were screened without success, and the results are shown in the Supporting Information. Below, we discuss the probing of these Fe additives in both the presence and absence of dioxygen.

**Figure 1:**
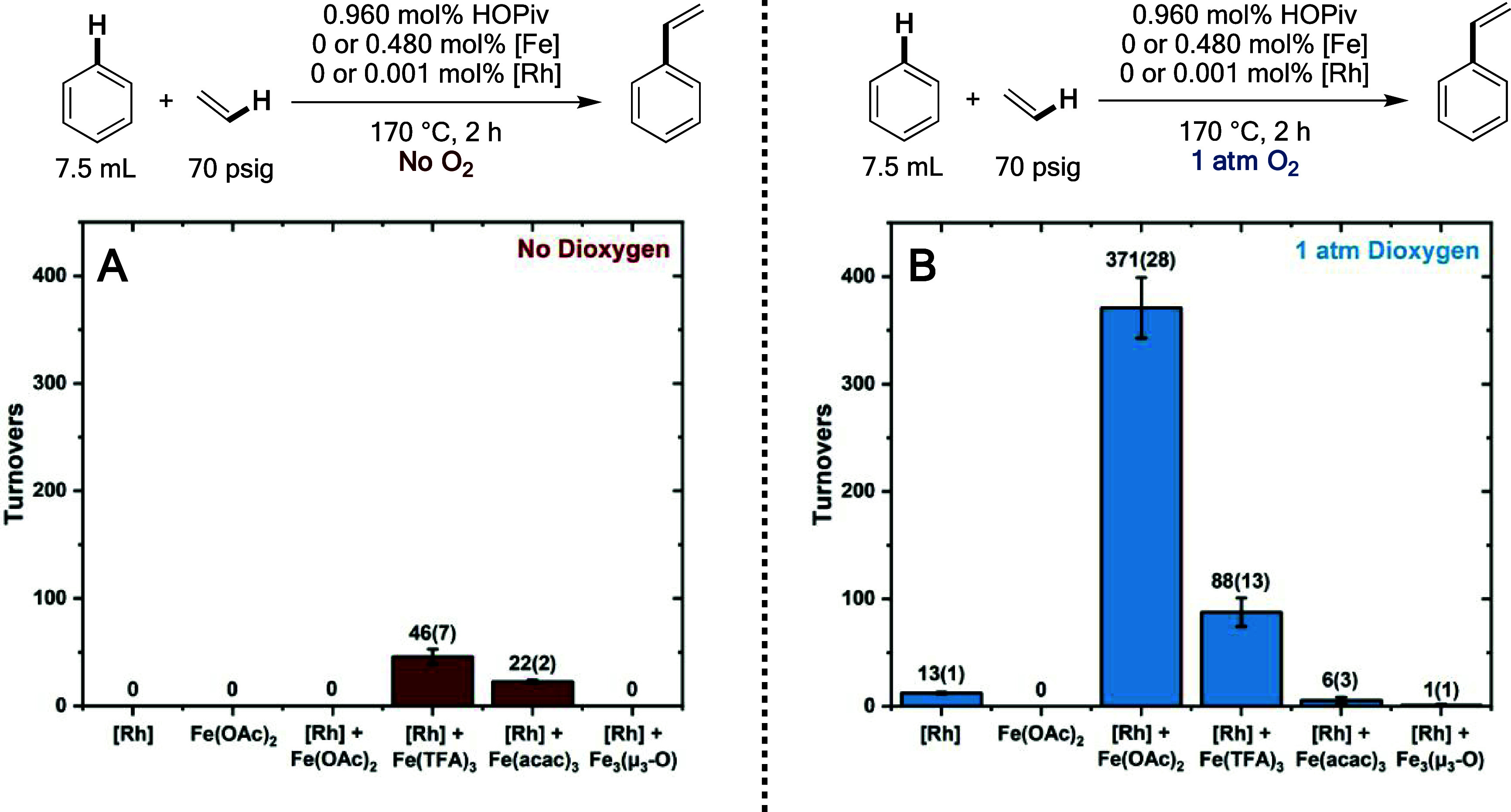
Screening of Fe(II) and Fe(III) carboxylate additives for Rh-catalyzed benzene ethenylation in the absence of dioxygen (A) and in the presence of 1 atm of dioxygen (B). Reaction conditions: 7.5 mL of benzene, 0.001 mol % (relative to benzene per single Rh atom) [(η^2^–C_2_H_4_)_2_Rh(μ–OAc)]_2_ if present, 0.480 mol % Fe(OAc)_2_, Fe(TFA)_3_, Fe(acac)_3_, or Fe_3_(μ_3_–O); (Fe_3_(μ_3_–O) = [Fe_3_(μ_3_–O)(μ–OPiv)_6_][OPiv]) (per single Fe atom) if present, 0.960 mol % HOPiv, 70 psig ethylene, 1 atm dioxygen or no dioxygen, 170 °C, 2 h. All data points reflect the average of a minimum of three independent reactions and error bars represent the standard deviation from multiple independent experiments.

As shown in Figure 1a, anaerobic benzene ethenylation reactions at 170 °C were performed using 7.5 mL of benzene and 70 psig of ethylene with 0.001 mol % (relative to benzene per single Rh atom) [(η^2^–C_2_H_4_)_2_Rh(μ–OAc)]_2_, 0.480 mol % (relative to benzene per single Fe atom) of Fe additive, and 0.960 mol % HOPiv. The HOPiv additive was used rather than HOAc because, upon heating and addition of dioxygen, HOPiv imparted complete solubility of the material formed from Fe(OAc)_2_ and Fe(TFA)_3_ whereas only partial solubility is achieved with HOAc or in the absence of carboxylic acid. In the absence of Fe additive and under anaerobic conditions, [(η^2^–C_2_H_4_)_2_Rh(μ–OAc)]_2_ is not active for styrene production. Also, the combination of [(η^2^–C_2_H_4_)_2_Rh(μ–OAc)]_2_ and Fe(OAc)_2_ is not active for styrene production, which indicates that Fe(II) cannot serve as an oxidant. Use of [Fe(μ_3_–O)(μ–OPiv)_6_][OPiv] as an additive in combination with [(η^2^–C_2_H_4_)_2_Rh(μ–OAc)]_2_ did not result in any detectable styrene product after 2 h, suggesting that [Fe(μ_3_–O)(μ–OPiv)_6_][OPiv] is not likely an active oxidant. The inactivity of [Fe(μ_3_–O)(μ–OPiv)_6_][OPiv] resembles our group’s previous finding that the related complex [Fe_3_^III^(μ_3_–O)(H_2_O)_2_(μ-TFA)_6_(TFA)](HTFA) is inactive for photodriven methane partial oxidation.^35^ The combination of [(η^2^–C_2_H_4_)_2_Rh(μ–OAc)]_2_ and Fe(TFA)_3_ results in 46(7) turnovers (TOs) of styrene (per Rh atom) after 2 h, which indicates that Fe(III) can serve as a direct oxidant in some formulations. Similar to the use of Fe(TFA)_3_, the use of Fe(acac)_3_ additive results in 22(2) TOs after 2 h. Notably, Fe(acac)_3_ is inactive as an oxidant when used in the absence of HOPiv, indicating that carboxylate ligands are likely necessary for catalysis (Table S1).

Next, as shown in Figure 1b, benzene ethenylation reactions in the presence of one atm of dioxygen at 170 °C were performed using 7.5 mL of benzene, 70 psig ethylene, 0.001 mol % [(η^2^–C_2_H_4_)_2_Rh(μ–OAc)]_2_ (relative to benzene per single Rh atom), 0.480 mol % (per single Fe atom) of Fe additive, and 0.960 mol % HOPiv. With [(η^2^–C_2_H_4_)_2_Rh(μ–OAc)]_2_ alone, ∼15 TOs of styrene were observed after 2 h, consistent with previously reported results.^59^ Under aerobic conditions, the combination of [(η^2^–C_2_H_4_)_2_Rh(μ–OAc)]_2_ and Fe(OAc)_2_ produced 371(28) TOs of styrene after 2 h, which is ∼28-fold more TOs than recorded in the absence of Fe(OAc)_2_. The catalysis is ∼95% selective to styrene after 2 h with small quantities of benzaldehyde, *trans*-stilbene, biphenyl, and vinyl pivalate; however, no phenyl pivalate or phenyl acetate was detected. The selectivity for styrene versus side products decreases over time as a result of the styrene product undergoing oxidation to benzaldehyde and oxidative hydrophenylation to form *trans*-stilbene, which is discussed in more detail later in this manuscript. As noted above, our attempted catalysis using Fe(OAc)_2_ in the absence of dioxygen did not yield styrene. In the presence of dioxygen, the white Fe(OAc)_2_ starting material rapidly undergoes a color change to maroon upon heating at 170 °C. When using [Fe(μ_3_–O)(μ–OPiv)_6_][OPiv] additive in the presence of dioxygen, statistically identical TOs of styrene were observed after 2 h compared to reactions performed in the absence of the Fe additive, suggesting that [Fe(μ_3_–O)(μ–OPiv)_6_][OPiv] does not likely serve as an oxidant and possibly deactivates the Rh based catalyst under these reaction conditions. In the presence of one atm of dioxygen, Fe(TFA)_3_ gave 88(13) TOs of styrene, which is ∼2-fold more than that observed in the absence of dioxygen. The use of Fe(acac)_3_ as the oxidant in the presence of 1 atm dioxygen results in 6(3) TOs after 2 h, which is less than what was observed for the reaction without Fe additive. In the absence of HOPiv, the use of Fe(acac)_3_, FeCl_3_, and FeCl_2_ additives results in the formation of trace quantities of styrene (Table S1). Having determined that the use of Fe(OAc)_2_ as an additive in the presence of dioxygen results in the most significant enhancement in reaction rate relative to catalysis without dioxygen, we performed studies of the effect of Fe(II) carboxylate and carboxylic acid identity (see the Supporting Information). It was found that a combination of Fe(OAc)_2_ and HOPiv resulted in optimal catalyst longevity and activity.

### Catalysis in the Absence of Dioxygen

**Figure 2:**
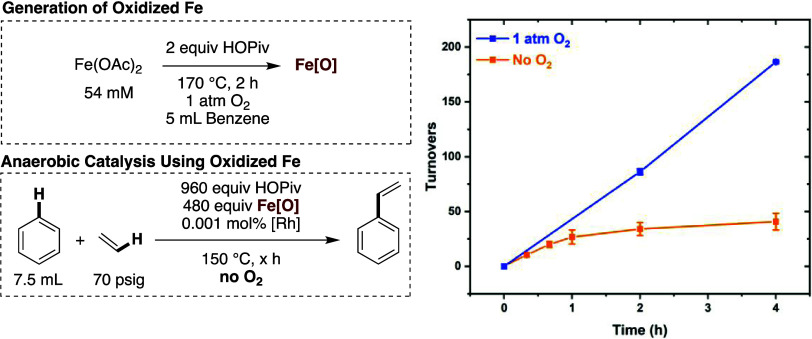
Kinetics of benzene ethenylation using the material generated by the reaction of Fe(OAc)_2_ and HOPiv with dioxygen under anaerobic conditions and comparison to the kinetics of an aerobic reaction in the presence of Fe(OAc)_2_. Reaction conditions: Reaction 1: 5 mL of benzene, 0.004 mol of Fe(OAc)_2_, 0.008 mol of HOPiv, 1 atm dioxygen, and 70 psig N_2_ 170 °C, 2 h. Reaction 2: 5 mL solutions from Reaction 1 were combined with [(η^2^–C_2_H_4_)_2_Rh(μ–OAc)]_2_ in 2.5 mL of benzene to give 7.5 mL of benzene, with 0.001 mol % [(η^2^–C_2_H_4_)_2_Rh(μ–OAc)]_2_, 480 equiv of Fe (relative to Rh per single Fe atom), 960 equiv of HOPiv, 70 psig ethylene, 150 °C. All data points reflect the average of a minimum of three independent reactions, and error bars represent the standard deviation from the multiple independent experiments.

It has been demonstrated that Rh and Pd-catalyzed arene alkenylation can be performed with Cu(II) carboxylates as the oxidant and limiting reagent in the absence of dioxygen.^61,63^ Following the reduction of Cu(II) to Cu(I), dioxygen can be used to regenerate Cu(II) in a step separate from catalysis at lower temperatures, preventing the undesired reaction of the styrene product or an active catalyst with dioxygen.^61^ This two-step process with a separate Cu(II) recycle step is similar to a commercial variant of the Wacker–Hoechst process.^80^ We speculated that a similar two-step reaction could be carried out using an oxidized Fe material. As shown in Figure 2, the use of material generated by the reaction of Fe(OAc)_2_ with dioxygen as the oxidant at anaerobic conditions was probed. To do so, Fe(OAc)_2_ and HOPiv were heated at 170 °C in benzene under one atm of dioxygen for 2 h. Next, [(η^2^–C_2_H_4_)_2_Rh(μ–OAc)]_2_ was added under a flow of dinitrogen, and dioxygen was removed from the reaction mixtures by flushing with dinitrogen. Following the removal of dioxygen, the reactors were pressurized with ethylene, and catalysis was performed at 150 °C.

The kinetics of benzene ethenylation in the absence of dioxygen were compared to an otherwise identical reaction in the presence of one atm of dioxygen. As demonstrated in Figure 2, the initial rate of catalysis in the absence of dioxygen is slightly slower than that of an analogous reaction performed in the presence of one atm of dioxygen. Additionally, the reaction in the absence of dioxygen stops after the production of 40(5) TOs of styrene, and the reaction undergoes a color change from bright maroon to dark brown, which is potentially consistent with the consumption of the active oxidant (see the Supporting Information for photographs).

If it is assumed that each Fe atom serves as one oxidizing equiv, 240 TOs of styrene would represent a 100% yield under conditions in which 480 equiv of Fe atoms relative to Rh are present. Thus, the observation of ∼40 TOs of styrene corresponds to a yield of approximately one-sixth if one oxidizing equiv per Fe atom is assumed. This observation could be consistent with (1) the active oxidant being unstable under the reaction conditions, (2) the active oxidant not being quantitatively generated, or (3) multiple Fe centers being necessary to store one oxidizing equiv.

### Studies of Fe Speciation

**Figure 3:**
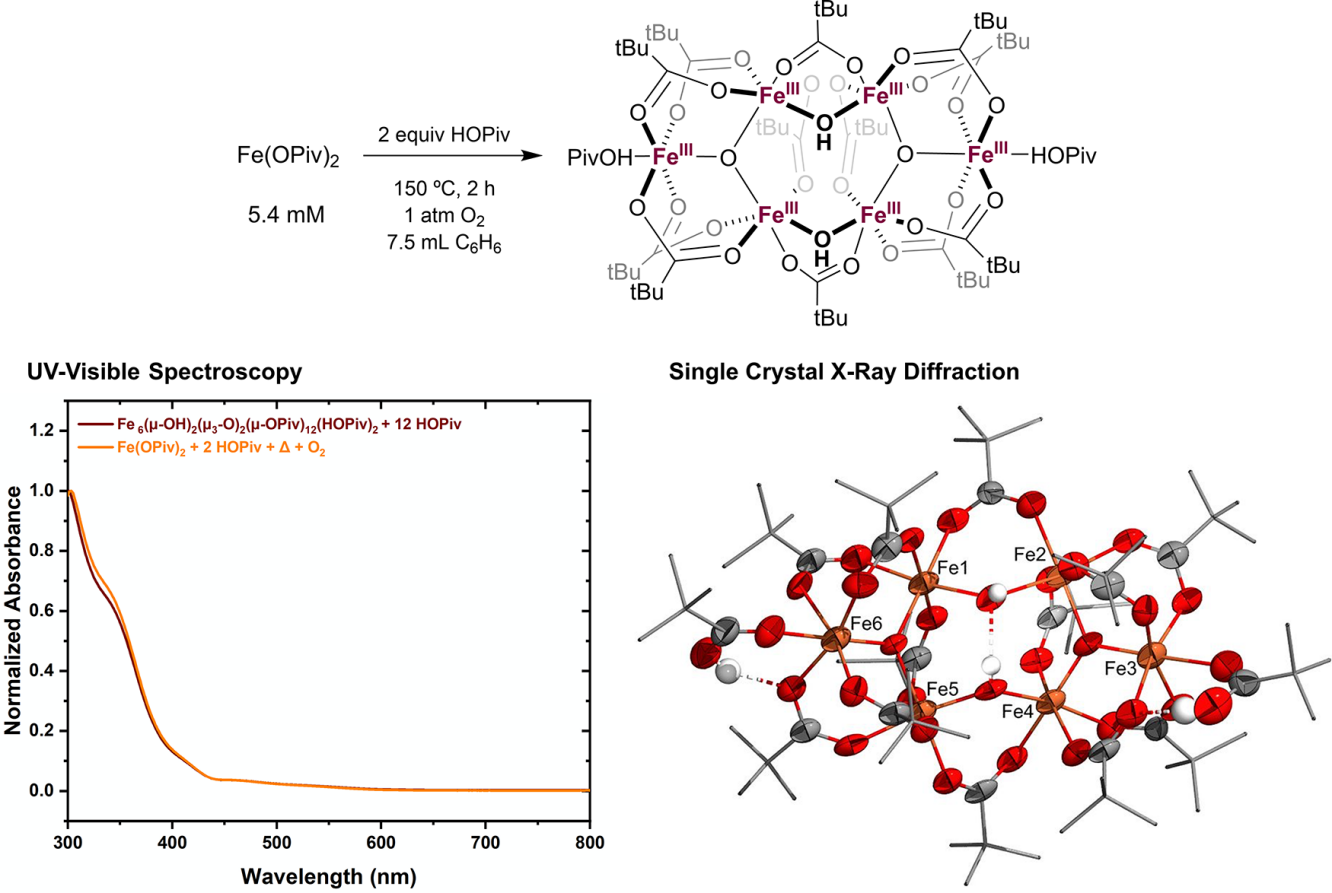
Generation of the oxidized Fe species formed under catalytic conditions and characterization by UV–visible spectroscopy and ORTEP of Fe_6_(μ–OH)_2_(μ_3_–O)_2_(μ–OPiv)_12_(HOPiv)_2_·C_6_H_6_ obtained from single-crystal X-ray diffraction. Reaction conditions: 7.5 mL of benzene, 5.4 mM Fe(OPiv)_2_, two equiv of HOPiv (relative to Fe(OPiv)_2_), 1 atm O_2_, 150 °C, 2 h. For the X-ray crystal structure, the benzene solvate molecule and H atoms from all C–H bonds are omitted for clarity, and ellipsoids are drawn at 50% probability. Only the major positions of the disordered atoms are shown. For the UV–visible spectroscopy experiment, 0.1 mL of the reaction solution was diluted in benzene to a total volume of 5 mL. The standard solution of Fe_6_(μ–OH)_2_(μ_3_–O)_2_(μ–OPiv)_12_(HOPiv)_2_ was prepared at 0.9 mM in 7.5 mL of benzene in the presence of 12 equiv of HOPiv (two equiv per Fe atom), and 0.1 mL of this solution was diluted to 5 mL in a benzene solution.

To understand the speciation of Fe under catalytic conditions, the active oxidant was obtained by heating Fe(OPiv)_2_ and 2 equiv of HOPiv at 150 °C under 1 atm of dioxygen for 2 h. This solution was characterized by single-crystal X-ray diffraction (XRD), UV–visible spectroscopy, and ^1^H NMR spectroscopy. From the reaction mixture, complex Fe_6_(μ–OH)_2_(μ_3_–O)_2_(μ–OPiv)_12_(HOPiv)_2_·C_6_H_6_ was identified by single-crystal XRD (Figure 3). Fe_6_(μ–OH)_2_(μ_3_–O)_2_(μ–OPiv)_12_(HOPiv)_2_ is similar to previously reported hexanuclear Fe(III) carboxylate structures bearing μ–OH ligands.^18,32^ UV–visible spectroscopy was performed on the oxidized material, and the obtained spectrum is nearly identical to that of Fe_6_(μ–OH)_2_(μ_3_–O)_2_(μ–OPiv)_12_(HOPiv)_2_ and 12 equiv of HOPiv present at the same concentration (Figure 3). Additionally, ^1^H NMR spectra of the oxidized material are similar to that of Fe_6_(μ–OH)_2_(μ_3_–O)_2_(μ–OPiv)_12_(HOPiv)_2_ and 12 equiv of HOPiv (Figures S11 and S17). These findings suggest that Fe_6_(μ–OH)_2_(μ_3_–O)_2_(μ–OPiv)_12_(HOPiv)_2_ is the dominant species under aerobic conditions, and are potentially consistent with Fe_6_(μ–OH)_2_(μ_3_–O)_2_(μ–OPiv)_12_(HOPiv)_2_ serving as the active oxidant. The observation of a yield of approximately one-sixth relative to total Fe atoms when performing anaerobic benzene ethenylation can likely be rationalized by hexanuclear Fe_6_(μ–OH)_2_(μ_3_–O)_2_(μ–OPiv)_12_(HOPiv)_2_ serving as one oxidizing equivalent (hereafter equiv).

**Scheme 2:**
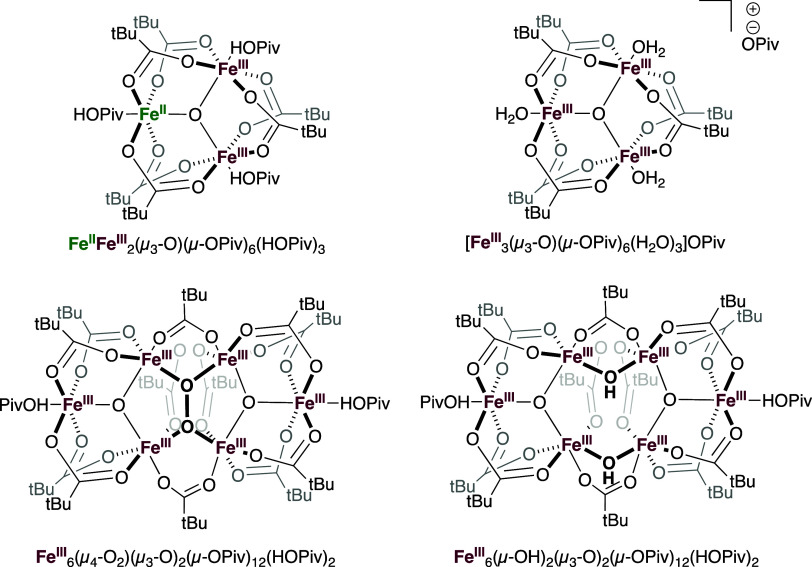
Fe Pivalate Complexes That Were Synthesized and Isolated to Probe Their Activity as Oxidants for Benzene Ethenylation in Aerobic and Anaerobic Conditions

As discussed above, the complex Fe_6_(μ–OH)_2_(μ_3_–O)_2_(μ–OPiv)_12_(HOPiv)_2_ is the dominant Fe species under aerobic conditions and the likely active oxidant. To further study Fe speciation, Fe_6_(μ–OH)_2_(μ_3_–O)_2_(μ–OPiv)_12_(HOPiv)_2_ was synthesized in addition to other multinuclear Fe complexes that could plausibly form under the reaction conditions and/or serve as intermediates in the formation of Fe_6_(μ–OH)_2_(μ_3_–O)_2_(μ–OPiv)_12_(HOPiv)_2_ (Scheme 2). These complexes included the all Fe(III) trimeric complex [Fe_3_^III^(μ_3_–O)(μ–OPiv)_6_(H_2_O)_3_][OPiv], the Fe(II)/Fe(III) mixed-valent complex Fe_2_^III^Fe^II^(μ_3_–O)(μ–OPiv)_6_(HOPiv)_3_, the dioxygen-bridged hexanuclear complex Fe_6_^III^(μ_4_–O_2_)(μ_3_–O)_2_(μ–OPiv)_12_(HOPiv)_2_, and the hydroxide-bridged hexanuclear complex Fe_6_^III^(μ–OH)_2_(μ_3_–O)_2_(μ–OPiv)_12_(HOPiv)_2_. Each multinuclear Fe pivalate complex was used as an additive for aerobic and anaerobic benzene ethenylation and compared to kinetics when using Fe(OAc)_2_ as the starting material.

**Figure 4:**
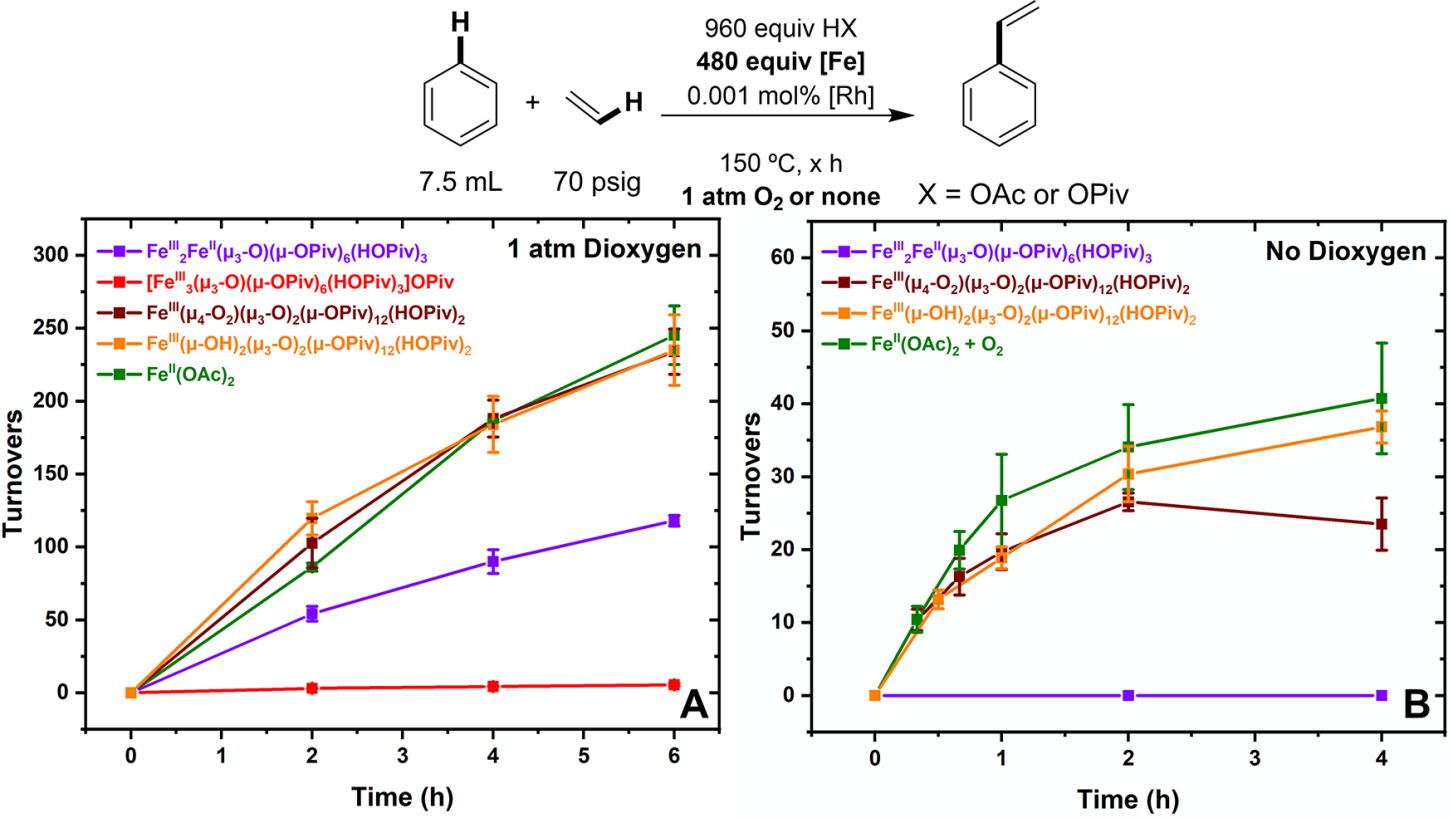
Kinetics of benzene ethenylation when Fe pivalate additives were used in the presence of HOAc under either aerobic or anaerobic conditions. Reaction conditions: 7.5 mL of benzene, 0.001 mol % (relative to benzene per single Rh atom) [(η^2^–C_2_H_4_)_2_Rh(μ–OAc)]_2_, 480 equiv (relative to Rh per single Fe atom) of Fe pivalate additive, 960 equiv of HOAc, 70 psig ethylene, 1 atm dioxygen, 150 °C, × hours. Reactions with Fe(OAc)_2_ were performed with 480 equiv of Fe(OAc)_2_ and 960 equiv of HOPiv. For the anaerobic reactions with Fe(OAc)_2_, 5 mL benzene, 0.0042 mol of Fe(OAc)_2_, and 0.0084 mol of HOPiv were heated under 1 atm dioxygen for 2 h, and 0.001 mol % [(η^2^–C_2_H_4_)_2_Rh(μ–OAc)]_2_ (relative to benzene per single Rh atom) were added to the reactors, and dioxygen was removed prior to the addition of ethylene. All data points reflect the average of a minimum of three independent reactions and error bars represent the standard deviation from the multiple independent experiments.

As shown in Figure 4a, benzene ethenylation reactions were studied in the presence of 1 atm of dioxygen using a 0.001 mol % loading of [(η^2^–C_2_H_4_)_2_Rh(μ–OAc)]_2_ (relative to benzene per single Rh atom) and 480 equiv of Fe pivalate additive (relative to Rh per single Fe atom). As discussed in the Supporting Information, carboxylate identity has a complicated but important effect on catalyst activity and longevity, and the reaction rate and longevity are optimized when a combination of OPiv and OAc sources are used. Accordingly, reactions were performed with 960 equiv of HOAc (relative to Rh). The cationic Fe pivalate complex [Fe_3_^III^(μ_3_–O)(μ–OPiv)_6_(H_2_O)_3_][OPiv] provided a rate of reaction similar to that observed in the absence of the Fe additive (with only dioxygen as the oxidant, Figure S18), suggesting that it cannot access an active Fe oxidant. The charge neutral trimeric complex Fe_2_^III^Fe^II^(μ_3_–O)(μ–OPiv)_6_(HOPiv)_3_, which has one formal Fe(II) center, was active but slower than the reaction performed with Fe(OPiv)_2_. The decreased rate with Fe_2_^III^Fe^II^(μ_3_–O)(μ–OPiv)_6_(HOPiv)_3_ might be attributable to its bound HOPiv ligands, as the reaction rate has an inverse dependence on the concentration of HOPiv (see below). The Fe_2_^III^Fe^II^(μ_3_–O)(μ–OPiv)_6_(HOPiv)_3_ complex is active under aerobic conditions whereas [Fe_3_^III^(μ_3_–O)(μ–OPiv)_6_(H_2_O)_3_][OPiv] is not active, which indicates that the reaction of dioxygen with a Fe(II) center is likely necessary to form an active Fe-based oxidant. The use of Fe_6_^III^(μ_4_–O_2_)(μ_3_–O)_2_(μ–OPiv)_12_(HOPiv)_2_ gave statistically identical results to Fe(OAc)_2_, suggesting that it is a possible intermediate or active oxidant. Likewise, the rate of reaction with Fe_6_^III^(μ–OH)_2_(μ_3_–O)_2_(μ–OPiv)_12_(HOPiv)_2_ was statistically identical to that observed with Fe(OAc)_2_.

Next, the kinetics of styrene production under otherwise identical conditions, but in the absence of dioxygen, were studied (Figure 4b). [Fe_3_^III^(μ_3_–O)(μ–OPiv)_6_(H_2_O)_3_][OPiv] is not an active oxidant and does not form an active oxidant under aerobic conditions, suggesting that neither its Fe(III) centers nor its μ_3_–O functionalities are capable of serving as an oxidant under the reaction conditions. Fe_2_^III^Fe^II^(μ_3_–O)(μ–OPiv)_6_(HOPiv)_3_, which was active under aerobic conditions, is inactive in the absence of dioxygen, suggesting that dioxygen must react with Fe_2_^III^Fe^II^(μ_3_–O)(μ–OPiv)_6_(HOPiv)_3_ to form an active oxidant. The complexes Fe_6_^III^(μ_4_–O_2_)(μ_3_–O)_2_(μ–OPiv)_12_(HOPiv)_2_ and Fe_6_^III^(μ–OH)_2_(μ_3_–O)_2_(μ–OPiv)_12_(HOPiv)_2_ give statistically identical results to those of material generated by heating Fe(OAc)_2_ with dioxygen. These results indicate that Fe_6_^III^(μ_4_–O_2_)(μ_3_–O)_2_(μ–OPiv)_12_(HOPiv)_2_ is a possible intermediate in the formation of Fe_6_^III^(μ–OH)_2_(μ_3_–O)_2_(μ–OPiv)_12_(HOPiv)_2_, and the results support the identification of Fe_6_^III^(μ–OH)_2_(μ_3_–O)_2_(μ–OPiv)_12_(HOPiv)_2_ as the likely active oxidant. The stoichiometry of the anaerobic reaction using Fe_6_^III^(μ–OH)_2_(μ_3_–O)_2_(μ–OPiv)_12_(HOPiv)_2_ is consistent with each equiv serving as one oxidizing equiv. Although Fe_6_^III^(μ–OH)_2_(μ_3_–O)_2_(μ–OPiv)_12_(HOPiv)_2_ has two hydroxide ligands, which could each serve to abstract a net hydrogen atom, the experimental results suggest that the oxidizing ability of the cluster deteriorates after its first oxidizing equiv is spent.

**Figure 5:**
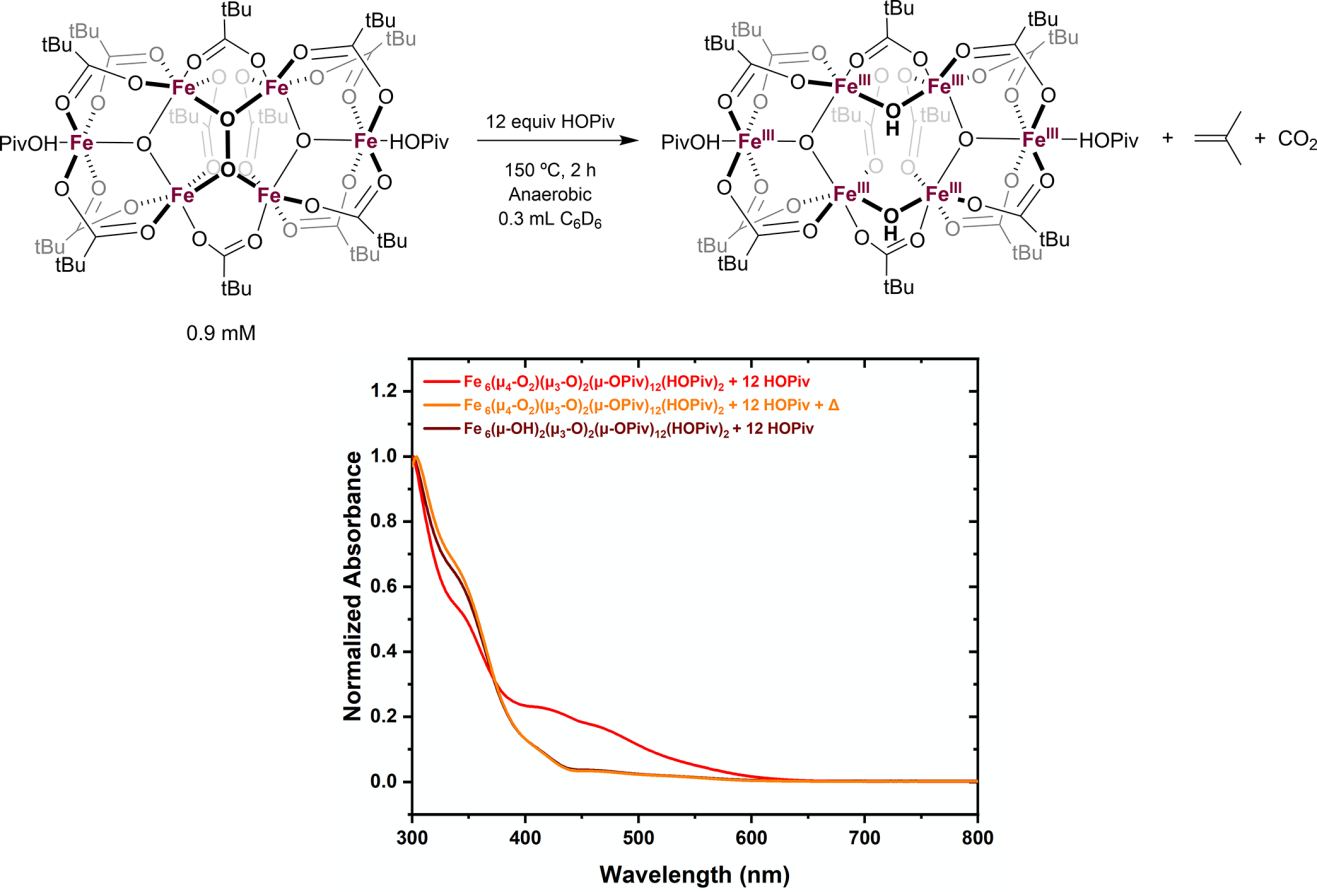
Reaction of Fe_6_^III^(μ_4_–O_2_)(μ_3_–O)_2_(μ–OPiv)_12_(HOPiv)_2_ with HOPiv to form Fe_6_^III^(μ–OH)_2_(μ_3_–O)_2_(μ–OPiv)_12_(HOPiv)_2_. Reaction conditions: 7.5 mL of C_6_H_6_, 0.9 mM Fe_6_^III^(μ_4_–O_2_)(μ_3_–O)_2_(μ–OPiv)_12_(HOPiv)_2_, 12 equiv (relative to hexanuclear Fe) of HOPiv, 150 °C, 2 h. UV–visible spectroscopy was carried out by combining 0.1 mL aliquots of the C_6_H_6_ solutions with 4.9 mL of benzene.

As discussed above, Fe_6_^III^(μ–OH)_2_(μ_3_–O)_2_(μ–OPiv)_12_(HOPiv)_2_ is the dominant Fe-based species under aerobic conditions and can access an active oxidant under anaerobic conditions. Fe_6_^III^(μ_4_–O_2_)(μ_3_–O)_2_(μ–OPiv)_12_(HOPiv)_2_ can also access an active oxidant under anaerobic conditions, suggesting that it is a likely intermediate in the formation of Fe_6_^III^(μ–OH)_2_(μ_3_–O)_2_(μ–OPiv)_12_(HOPiv)_2_. To probe the intermediacy of Fe_6_^III^(μ_4_–O_2_)(μ_3_–O)_2_(μ–OPiv)_12_(HOPiv)_2_ in the formation of Fe_6_^III^(μ–OH)_2_(μ_3_–O)_2_(μ–OPiv)_12_(HOPiv)_2_, and identify the source of hydrogen atoms in Fe_6_^III^(μ–OH)_2_(μ_3_–O)_2_(μ–OPiv)_12_(HOPiv)_2_, ^1^H NMR and UV–visible spectroscopy were used. Upon heating Fe_6_^III^(μ_4_–O_2_)(μ_3_–O)_2_(μ–OPiv)_12_(HOPiv)_2_ and 12 equiv of HOPiv (relative to the hexanuclear Fe complex) under anaerobic conditions, a color change from maroon to dark orange is observed. UV–visible spectroscopy was performed on the reaction mixtures before and after heating and the results showed that the material formed gives a spectrum nearly identical to that of Fe_6_^III^(μ–OH)_2_(μ_3_–O)_2_(μ–OPiv)_12_(HOPiv)_2_ and 12 equiv of HOPiv, and also nearly identical to the material generated by the reaction of Fe(OPiv)_2_ and HOPiv with dioxygen (Figure 5). This reaction was also performed in C_6_D_6_, and the recorded ^1^H NMR spectra are consistent with the conversion of Fe_6_^III^(μ_4_–O_2_)(μ_3_–O)_2_(μ–OPiv)_12_(HOPiv)_2_ to Fe_6_^III^(μ–OH)_2_(μ_3_–O)_2_(μ–OPiv)_12_(HOPiv)_2_ (Figures S15 and S17). Additionally, isobutene was observed by ^1^H NMR and gas chromatography–mass spectrometry (GC-MS), and CO_2_ was observed by GC-MS (Figure S14). The observation of isobutene and CO_2_ suggests that Fe_6_^III^(μ_4_–O_2_)(μ_3_–O)_2_(μ–OPiv)_12_(HOPiv)_2_ mediates the oxidative decarboxylation of HOPiv to form isobutene and CO_2_, resulting in the formation of Fe_6_^III^(μ–OH)_2_(μ_3_–O)_2_(μ–OPiv)_12_(HOPiv)_2_ after abstraction of two hydrogen atoms from HOPiv. Acetone formation was also observed, potentially suggesting that isobutene is readily oxidatively cleaved by an intermediate that forms during this process. Performing reactions under otherwise identical conditions but with Fe_6_^III^(μ–OH)_2_(μ_3_–O)_2_(μ–OPiv)_12_(HOPiv)_2_ results in no significant isobutene or CO_2_ formation, suggesting that Fe_6_^III^(μ–OH)_2_(μ_3_–O)_2_(μ–OPiv)_12_(HOPiv)_2_ cannot readily oxidatively decarboxylate HOPiv (Figures S13 and S16). When using Fe(OAc)_2_ and 2 equiv of HOPiv, minor quantities of isobutene are observed and methyl acetate is the major product (Figure S12). This suggests that the two H atoms of Fe_6_^III^(μ–OH)_2_(μ_3_–O)_2_(μ–OPiv)_12_(HOPiv)_2_ originate predominantly from two equiv of HOAc, resulting in the formation of methyl acetate and CO_2_.

The formation of Fe_6_^III^(μ–OH)_2_(μ_3_–O)_2_(μ–OPiv)_12_(HOPiv)_2_ as a result of HOPiv oxidative decarboxylation when the Fe_6_^III^(μ_4_–O_2_)(μ_3_–O)_2_(μ–OPiv)_12_(HOPiv)_2_ starting material is used resembles previous examples of Fe μ–OH formation as a result of H atom abstraction. For example, the Lippard group reported oxidative *N*-dealkylation of amine ligands upon exposure of carboxylate-bridged binuclear Fe(II) structures to dioxygen, which produces a Fe_2_(μ–OH)_2_(μ-carboxylate)_2_ core.^81−83^ The Mukhopadhyay and Kirschenbaum groups reported pyruvic acid oxidation by a binuclear Fe μ–O complex, which forms a μ–OH functionality after pyruvic acid oxidation.^84^ Similarly, the Kovacs group reported the formation of a Fe_2_(μ–OH)_2_ core upon the reaction of mononuclear Fe(II) complexes with dioxygen, which forms an intermediate, which abstracts an H atom from the acetonitrile solvent.^85^ It was found that the formed Fe_2_(μ–OH)_2_ complex is capable of abstracting H atoms from 9,10-dihydroanthracene, which contains substantially weaker C–H bonds than acetonitrile. These previous studies mirror our findings that Fe_6_^III^(μ_4_–O_2_)(μ_3_–O)_2_(μ–OPiv)_12_(HOPiv)_2_, or a possible Fe(IV) intermediate that it forms, can abstract H atoms from HOPiv. The formed complex Fe_6_^III^(μ–OH)_2_(μ_3_–O)_2_(μ–OPiv)_12_(HOPiv)_2_, which is dominant under catalytic conditions, is likely a sufficiently strong oxidant to oxidize Rh–H, forming H_2_O as the byproduct.

**Scheme 3:**
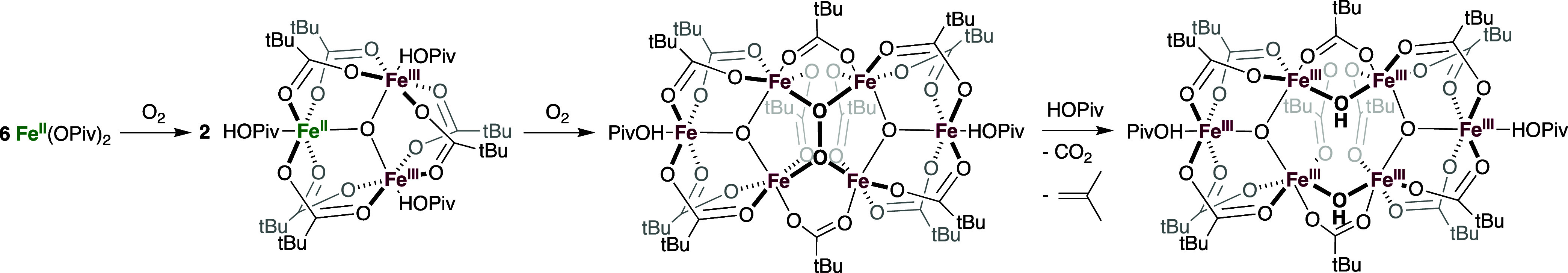
Proposed Mechanism of Fe_6_^III^(μ–OH)_2_(μ_3_–O)_2_(μ–OPiv)_12_(HOPiv)_2_ Formation from Fe(OPiv)_2_, HOPiv, and dioxygen

From the findings above, the following mechanism of Fe_6_^III^(μ–OH)_2_(μ_3_–O)_2_(μ–OPiv)_12_(HOPiv)_2_ formation from Fe(OPiv)_2_ and dioxygen is proposed (Scheme 3): six equiv of Fe(OPiv)_2_ react with one equiv of dioxygen to form two equiv of Fe_2_^III^Fe^II^(μ_3_–O)(μ–OPiv)_6_(HOPiv)_3_. Next, two equiv of Fe_2_^III^Fe^II^(μ_3_–O)(μ–OPiv)_6_(HOPiv)_3_ activate one equiv of dioxygen to form Fe_6_^III^(μ_4_–O_2_)(μ_3_–O)_2_(μ–OPiv)_12_(HOPiv)_2_. The complex Fe_6_^III^(μ_4_–O_2_)(μ_3_–O)_2_(μ–OPiv)_12_(HOPiv)_2_ then mediates oxidative decarboxylation of one equiv of HOPiv to form Fe_6_^III^(μ–OH)_2_(μ_3_–O)_2_(μ–OPiv)_12_(HOPiv)_2_, one equiv of isobutene and one equiv of CO_2_.

**Scheme 4:**
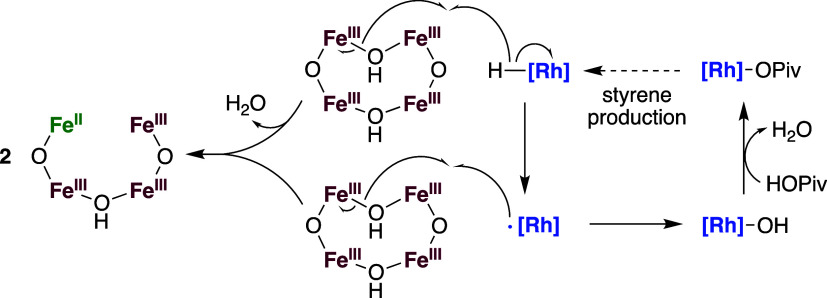
Proposed Mechanism of Rh–H Oxidation with 2 equiv of Fe_6_^III^(μ–OH)_2_(μ_3_–O)_2_(μ–OPiv)_12_(HOPiv)_2_

The active site of the proposed oxidant Fe_6_^III^(μ–OH)_2_(μ_3_–O)_2_(μ–OPiv)_12_(HOPiv)_2_ is likely its μ–OH functional groups, and, as discussed above, the reaction stoichiometry is consistent with each hexanuclear cluster serving as one oxidizing equiv. In one potential mechanism (Scheme 4), a Fe^III^–OH–Fe^III^ functionality performs one-electron oxidation of an Rh–H intermediate, abstracting a hydrogen atom to form one Fe^II^, water, and Rh•, which could undergo reaction with a μ–OH functionality in a second equiv of Fe_6_^III^(μ–OH)_2_(μ_3_–O)_2_(μ–OPiv)_12_(HOPiv)_2_ to form an Rh–OH intermediate. This Rh–OH intermediate could subsequently be protonated by an equiv of HOPiv to regenerate the starting Rh–OPiv intermediate and release water.^86^ If Rh–OH protonation is occurring, this could rationalize improved catalysis when more acidic carboxylic acids are present (see the Supporting Information), in addition to the observed *k*_H_/*k*_D_ of 1.19(2) when using HOPiv versus DOPiv (see below), although other explanations for the positive role of carboxylic acid are possible.

**Figure 6:**
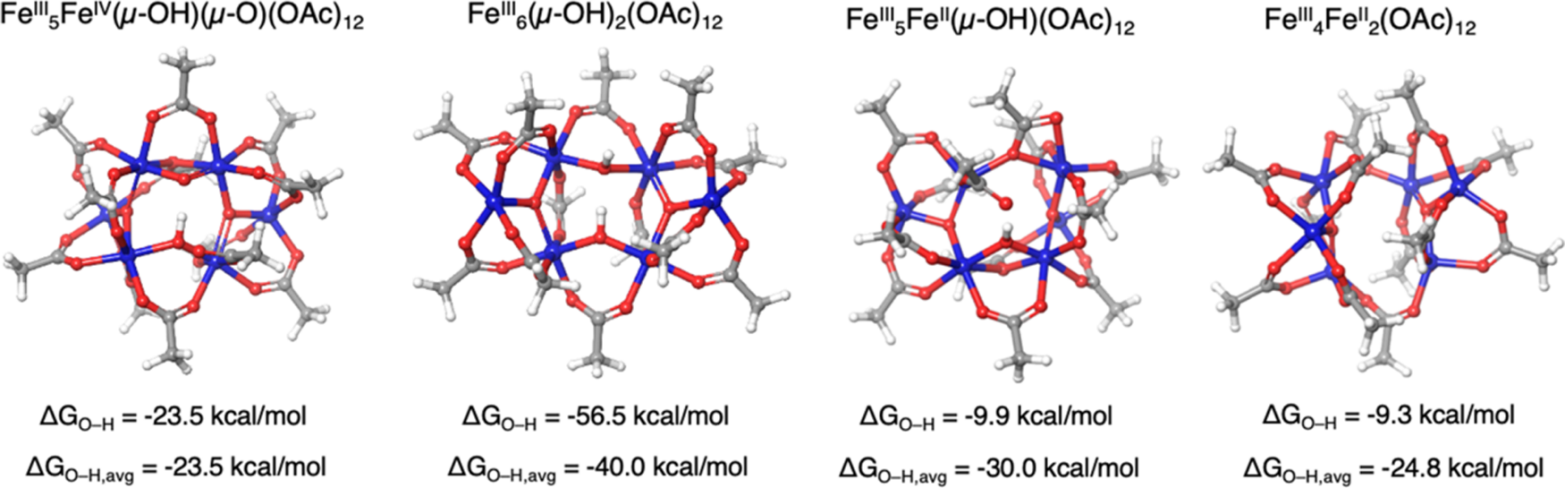
DFT-optimized species along the Fe_6_ hydrogenation sequence.

We employed density functional theory (DFT) calculations to understand how Fe_6_^III^(μ_4_–O_2_)(μ_3_–O)_2_(μ–OPiv)_12_(HOPiv)_2_ is converted to Fe_6_^III^(μ–OH)(μ–OH_2_)(μ_3_–O)_2_(μ–OPiv)_12_(HOPiv)_2_, and to understand why the complex apparently is not hydrogenated beyond this point. We are particularly interested in the strength of the O–H bonds formed during this conversion. For simplicity, we truncate our pivalates to acetates and remove pivalic acids (see Computational Methods for more details). DFT-optimized structures for the species of interest are depicted in Figure 6.

Starting with Fe_6_^III^(μ_4_–O_2_)(μ_3_–O)_2_(OAc)_12_, DFT predicts the first O–H bond (Δ*G*_O–H_) to incur −23.5 kcal/mol of stabilization, resulting in the formation of Fe_6_^III^(μ–OH)(μ–O)(μ_3_–O)_2_(OAc)_12_. The next logical hydrogenation would occur at the remaining μ–O bond to afford Fe_6_^III^(μ–OH)_2_(μ_3_–O)_2_(OAc)_12_. This second O–H bond yields Δ*G*_O–H_ = −56.5 kcal/mol, which is −33.0 kcal/mol stronger than the prior step. Further hydrogenation yields Fe_6_^III^(μ–OH)(μ–OH_2_)(μ_3_–O)_2_(OAc)_12_. DFT predicts that the third hydrogenation will result in Δ*G*_O–H_ = −9.9 kcal/mol, which is +46.6 kcal/mol weaker than the prior step. This weaker O–H bond is likely because μ–OH is being converted to μ–OH_2_, whereas the prior O–H bonds were formed by the conversion of μ–O to μ–OH. We suspect that the thermodynamic driving force for converting μ–OH to μ–OH_2_ is less than the driving force for converting μ–O to μ–OH because the O–H bond is weaker than the HO–H bond in these complexes. We find that this third hydrogenation leads to the dissociation of water from the complex, forming Fe_6_^III^(μ–OH)(μ_3_–O)_2_(OAc)_12_ + H_2_O. While not observed experimentally, Fe_6_^III^(μ–OH)(μ_3_–O)_2_(OAc)_12_ could hypothetically hydrogenate once more to form Fe_6_^III^(μ–OH_2_)(μ_3_–O)_2_(OAc)_12_; DFT predicts this hydrogenation to be stable by only −9.3 kcal/mol. This fourth and final hydrogenation is accompanied by dissociation of the second water to afford Fe_6_^III^(μ_3_–O)_2_(OAc)_12_ + H_2_O. Our experimental observations find that the first two hydrogens come from HOPiv, while the third hydrogenation occurs through oxidation of a Rh–H species by two equiv of some hexanuclear Fe. One interpretation of these results is that the first two hydrogenations are strong, the third hydrogenation is relatively weak, and the fourth hydrogenation does not occur. DFT agrees with this interpretation, predicting the first two O–H bonds (−23.5 and −56.5 kcal/mol, respectively) being strongest, the third O–H bond (−9.9 kcal/mol) being relatively weak, and the fourth O–H bond (−9.3 kcal/mol) being weakest.

### Mechanistic Studies

On the basis of the observations above (which include identification of the likely active Fe oxidant and importance of carboxylate identity), mechanistic studies of styrene production from benzene and ethylene using a combination of HOPiv and Fe(OAc)_2_ under aerobic conditions at 150 °C were performed.

### Order in Rh

**Figure 7:**
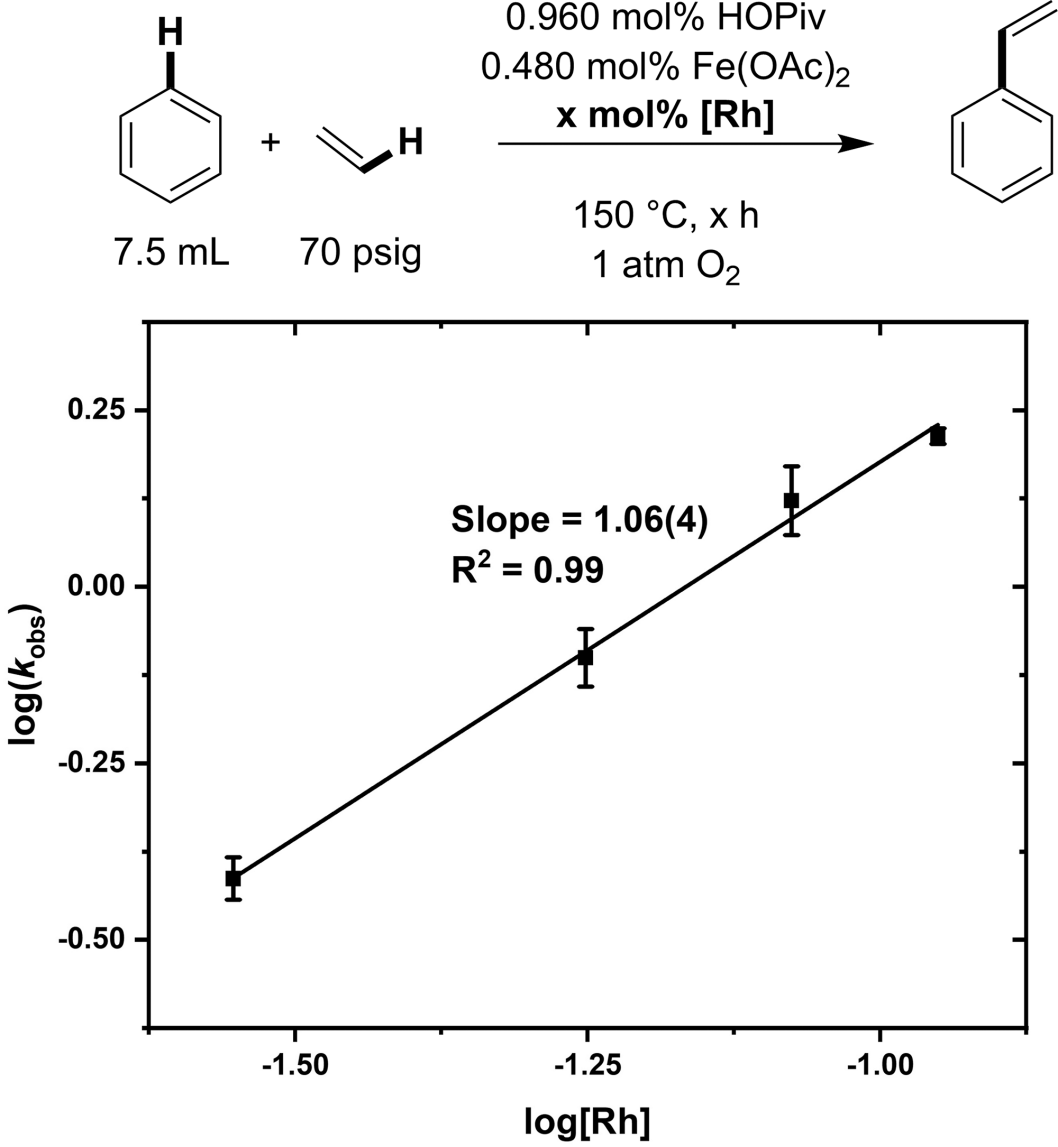
Log–log plot for the dependence of the initial reaction rate on the [(η^2^–C_2_H_4_)_2_Rh(μ–OAc)]_2_ concentration. Reaction conditions: 7.5 mL of benzene, × mol % (relative to benzene per single Rh atom) [(η^2^–C_2_H_4_)_2_Rh(μ–OAc)]_2_, 0.480 mol % Fe(OAc)_2_, 0.960 mol % HOPiv, 70 psig ethylene, 1 atm dioxygen, 150 °C. All data points reflect the average of a minimum of three independent reactions, and error bars represent the standard deviation from the multiple independent experiments. Note: for these experiments, rates were calculated based on TOs at the 2 h time point because catalyst longevity worsens as the catalyst loading is decreased.

Previously, our group reported an order of 1.54(4) in Rh when using Cu(II) carboxylates as the oxidant under anaerobic conditions at 120 °C.^87^ The observation of order in Rh between one and two was attributed to a complex equilibrium between mono- and bis-Rh active catalysts, for which the bis-Rh complex was proposed to be the more active catalyst. To determine whether a similar equilibrium occurs with Fe carboxylate additives, the order in [(η^2^–C_2_H_4_)_2_Rh(μ–OAc)]_2_ was studied. As shown in Figure 7, a near first-order dependence on Rh concentration was observed, suggesting that either (1) mono- and bis-Rh complexes provide similar activity or (2) interconversion between mono- and bis-Rh complexes does not occur under catalytic conditions.

### Arene C–H Activation

**Figure 8:**
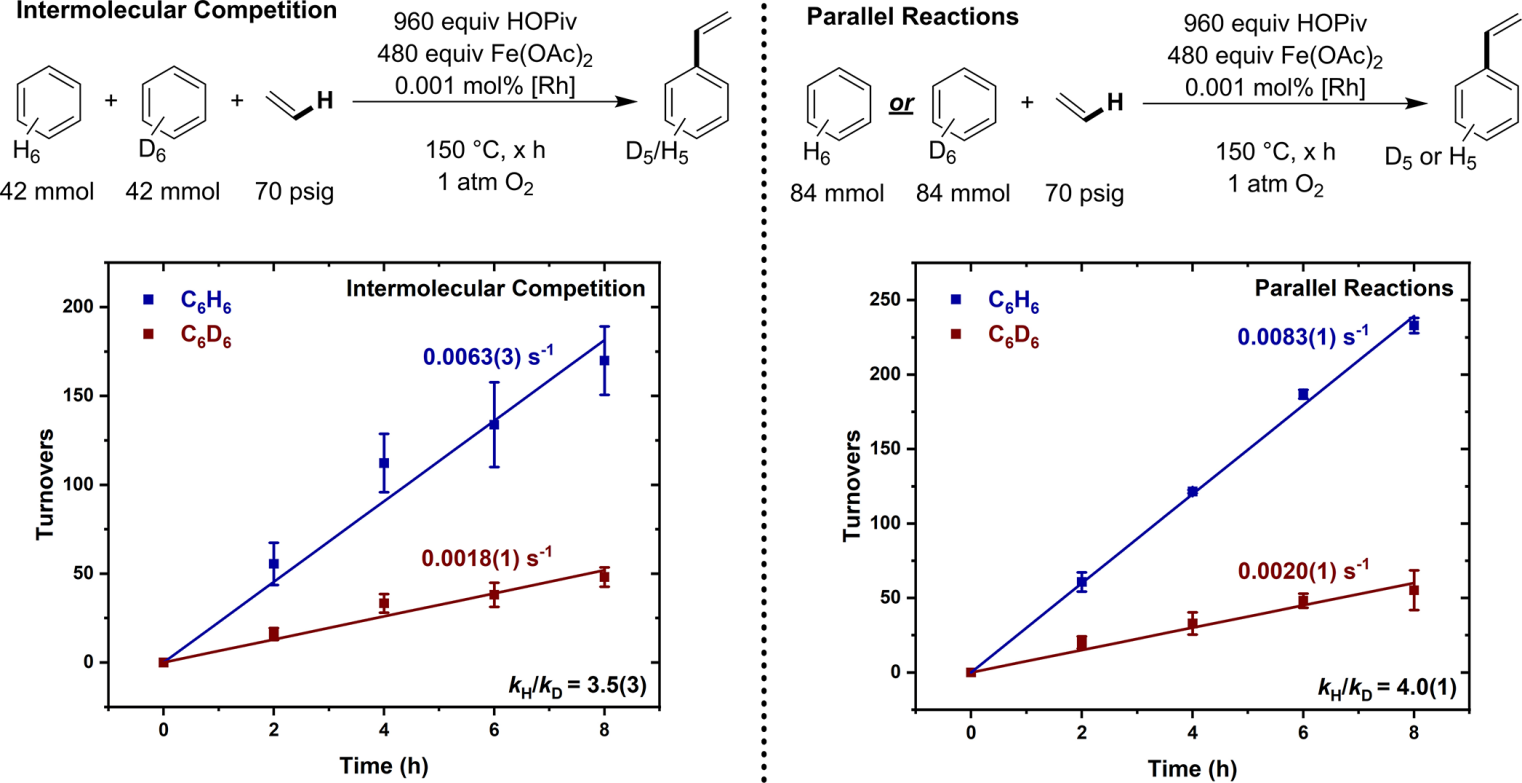
Kinetic isotope effect intermolecular competition experiments with equimolar quantities of C_6_H_6_ and C_6_D_6_ with Fe oxidants. Reaction conditions: intermolecular competition: 0.042 mol benzene, 0.042 mol benzene-*d*_6_, 0.001 mol % (relative to total moles of benzene and benzene-*d*_6_ per single Rh atom) [(η^2^–C_2_H_4_)_2_Rh(μ–OAc)]_2_, 480 equiv (relative to single Rh atom) of Fe(OAc)_2_, 960 equiv of HOPiv, 70 psig ethylene, 1 atm dioxygen, 150 °C. Reaction conditions: parallel reactions: 0.084 mol benzene or benzene-*d*_6_, 0.001 mol % (relative to benzene or benzene-*d*_6_ per single Rh atom) [(η^2^–C_2_H_4_)_2_Rh(μ–OAc)]_2_, 480 equiv (relative to single Rh atom) of Fe(OAc)_2_, 960 equiv of HOPiv, 70 psig ethylene, 1 atm dioxygen, 150 °C. All data points reflect the average of a minimum of three independent reactions and error bars represent the standard deviation from the multiple independent experiments.

Kinetic isotope effect experiments utilized benzene in intermolecular competition reactions (with equimolar quantities of benzene and benzene-*d*_6_) and independently performed (parallel) reactions. KIEs of 3.53(3) and 4.0(1) were observed for intermolecular competition and parallel reactions, respectively (Figure 8). The two measured KIEs give reasonable agreement, considering the standard deviations. The observation of primary kinetic isotope effects in both experiments is consistent with benzene C–H activation occurring before or during the rate-limiting step. Given the necessity of carboxylate ligands for the reaction to occur (see above), it is likely that C–H activation occurs either by a concerted metalation-deprotonation or by a stepwise process involving arene C–H activation and subsequent carboxylic acid reductive elimination. Both of these pathways have been studied previously for Rh catalysis with Cu(II) carboxylate oxidants using DFT, and lower barriers are generally obtained for the stepwise process involving oxidative addition followed by reductive elimination when Rh(I) is used as the catalyst precursor.^58,77,87^ In contrast, for Pd(II) catalysis the barrier for concerted metalation-deprotonation was found to be substantially lower than the stepwise oxidative addition/reductive elimination process.^66^ Previously, a KIE of 3.3(2) was observed for anaerobic catalysis with Cu(OPiv)_2_ as the oxidant and 2.7(6) for catalysis with dioxygen as the oxidant.^59,62^ The different KIE values observed as a function of oxidant identity are consistent with different active catalysts and reaction mechanisms existing for catalysis with each oxidant.

**Figure 9:**
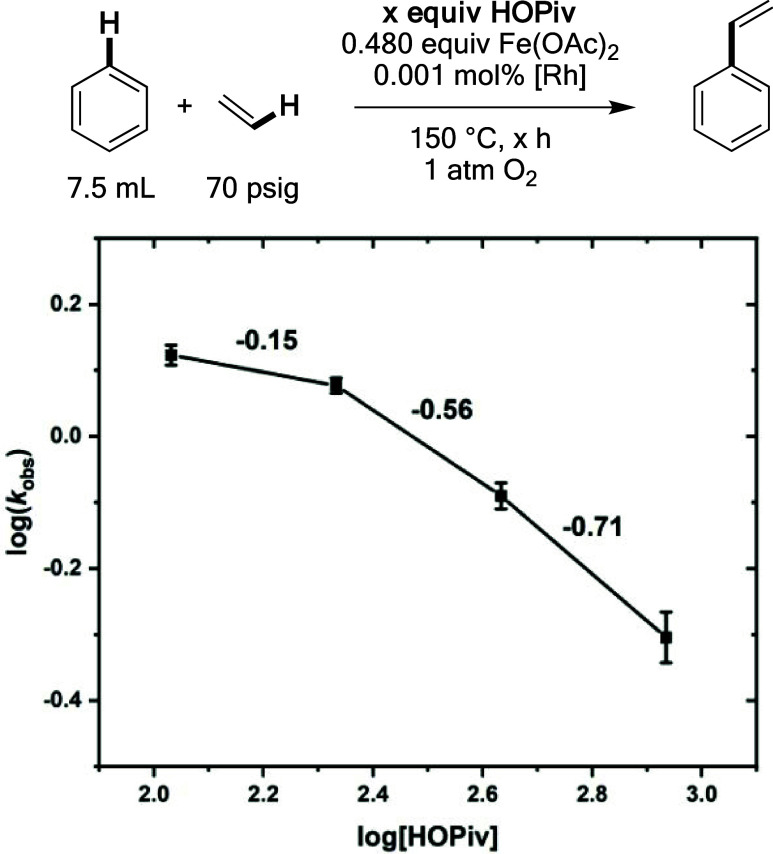
Log–log plot for the dependence of the initial reaction rate on the HOPiv concentration. Reaction conditions: 7.5 mL of benzene, 0.001 mol % (relative to benzene per single Rh atom) [(η^2^–C_2_H_4_)_2_Rh(μ–OAc)]_2_, 480 equiv of Fe(OAc)_2_, × equiv of HOPiv, 70 psig ethylene, 1 atm dioxygen, 150 °C. All data points reflect the average of a minimum of three independent reactions, and error bars represent the standard deviation from the multiple independent experiments. Note: the values shown to the right of the trendline represent the slope between each two data points, and are intended only to provide context for the dependence of the reaction rate on HOPiv concentration.

Next, the dependence of the reaction rate on the concentration of HOPiv was studied. As shown in Figure 9, the reaction has an inverse dependence in HOPiv concentration, which is consistent with three likely possibilities: (1) the acidic proton of HOPiv enhancing the rate of reverse arene C–H activation, (2) coordination of HOPiv to Rh or Fe inhibiting the reaction, or (3) increasing the OPiv:OAc ratio results in decreased activity.

**Figure 10:**
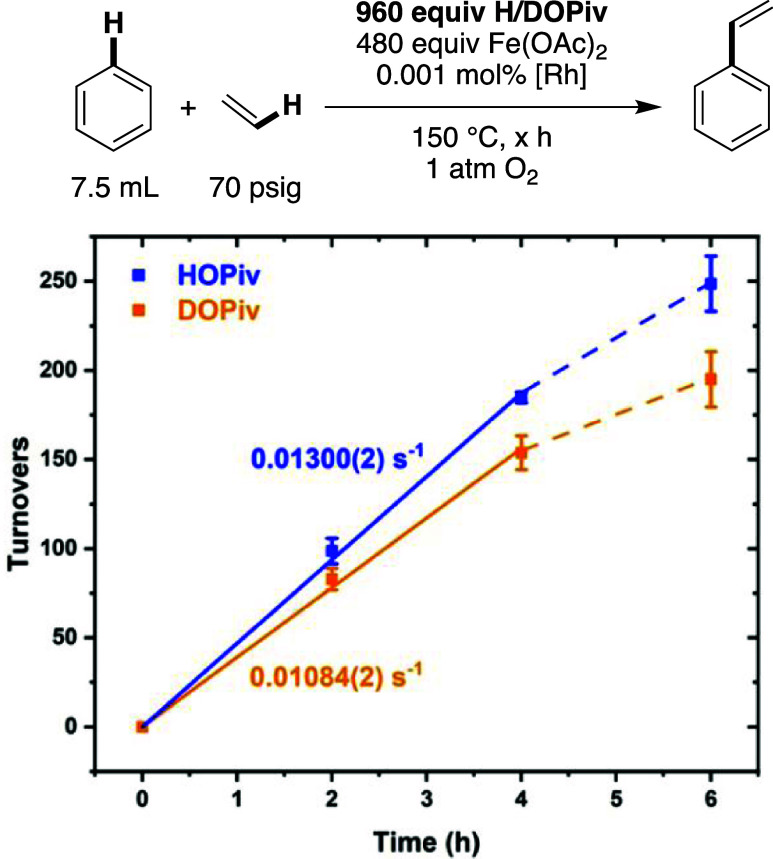
Kinetic isotope effect experiments for benzene ethenylation using HOPiv or DOPiv in independently performed reactions. Reaction conditions: 7.5 mL of benzene, 0.001 mol % (relative to benzene per single Rh atom) [(η^2^–C_2_H_4_)_2_Rh(μ–OAc)]_2_, 480 equiv of Fe(OAc)_2_, 960 equiv of HOPiv or DOPiv, 70 psig ethylene, 1 atm dioxygen, 150 °C. All data points reflect the average of a minimum of three independent reactions and error bars represent the standard deviation from the multiple independent experiments.

To determine whether the observed dependence on carboxylic acid concentration is related to the acidic proton, kinetic isotope experiments with HOPiv and pivalic acid-*d*_1_ (DOPiv) were performed (Figure 10). A KIE (*k*_H_/*k*_D_) of 1.19(2) was measured, indicating a small KIE, suggesting that either the acidic proton of HOPiv is not involved in a kinetically relevant step or there are competitive effects from multiple steps in the catalytic cycle.

**Scheme 5:**
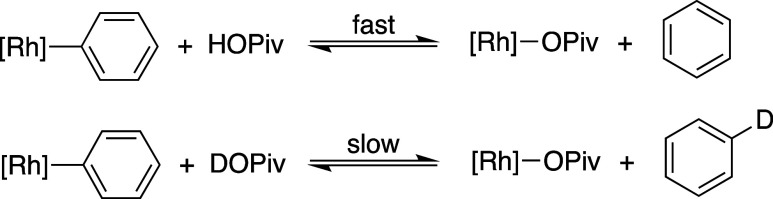
Demonstration of the Anticipated Relative Rates of Rh–Ph Protonation by HOPiv or DOPiv

A potentially relevant reaction for the acidic proton of HOPiv to undergo is protonolysis of a Rh–Ph intermediate in a reverse of the benzene C–H activation step (Scheme 5), which would likely be more rapid with HOPiv than with DOPiv. Since the undesired reverse C–H activation would be faster with HOPiv than with DOPiv, the rate of styrene production would consequently be slower with HOPiv than with DOPiv, which would likely result in an inverse KIE in the absence of other effects.^88,89^

**Figure 11:**
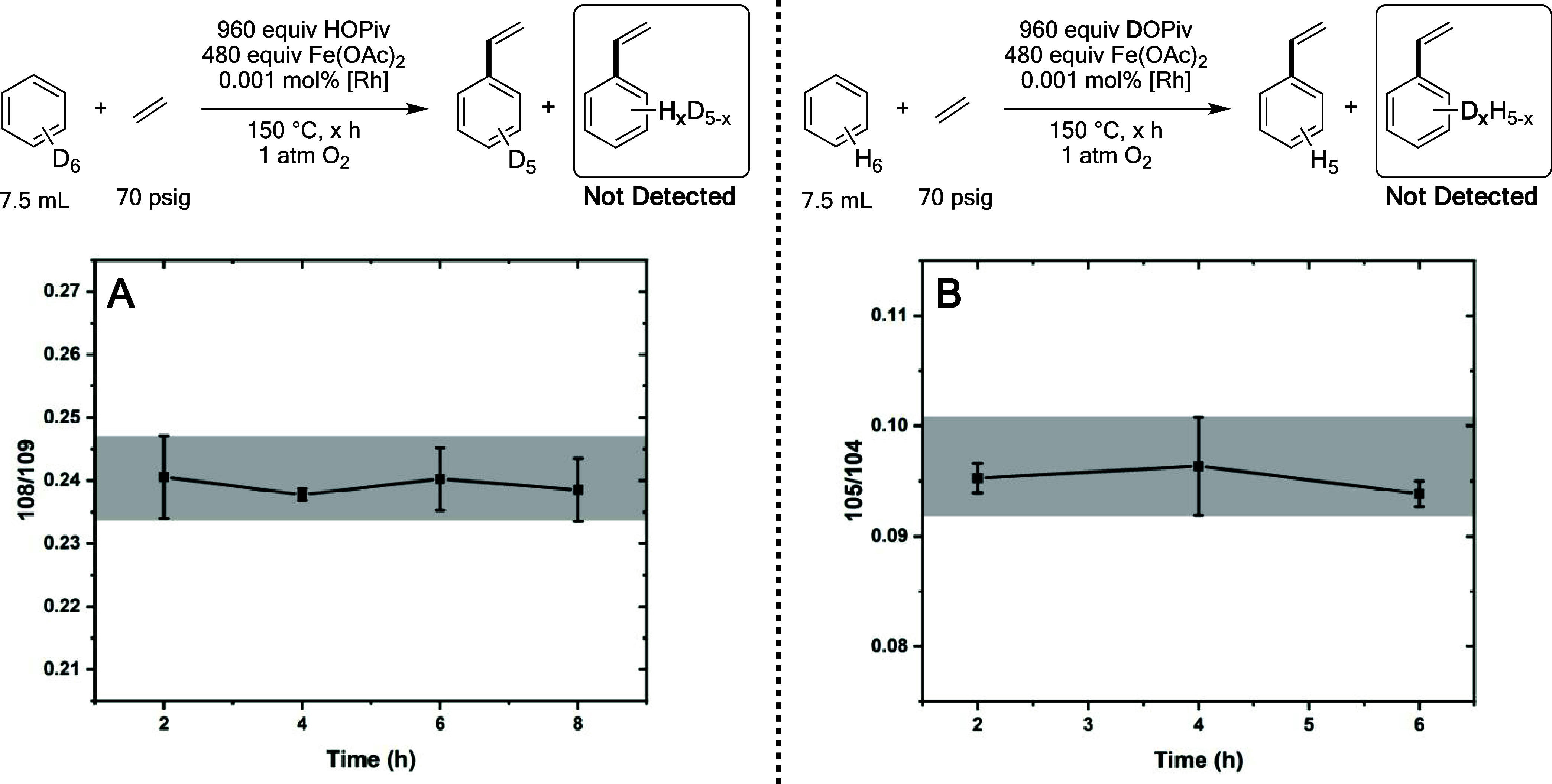
H/D scrambling for benzene ethenylation using HOPiv and C_6_D_6_ or DOPiv and C_6_H_6_. Reaction conditions: 7.5 mL of C_6_H_6_ or C_6_D_6_, 0.001 mol % (relative to benzene per single Rh atom) [(η^2^–C_2_H_4_)_2_Rh(μ–OAc)]_2_, 480 equiv of Fe(OAc)_2_, 960 equiv of DOPiv or HOPiv, 70 psig ethylene, 1 atm dioxygen, 150 °C. All data points reflect the average of a minimum of three independent reactions, and error bars represent the standard deviation from the multiple independent experiments. The gray shaded boxes are superimposed over the largest standard deviation observed, and demonstrate that no statistically significant change over time is observed for either experiment.

To determine if benzene C–H activation is reversible, deuterium or proton incorporation into the styrene product was studied using GC-MS for reactions using a combination of C_6_H_6_ and DOPiv or C_6_D_6_ and HOPiv. As shown in Figure 11a, the ratio of the *m*/*Z* = 105 to *m*/*Z* = 104 peak areas, which corresponds to monodeutero and per-protio styrene, respectively, does not undergo a statistically significant change over time when using a combination of C_6_H_6_ and DOPiv. Similarly, in Figure 11b, when using C_6_D_6_ and HOPiv, the ratio of *m*/*Z* = 108 to *m*/*Z* = 109, which correspond to styrene-*d*_4_ and styrene-*d*_5_, respectively, does not change over time. The observation that the isotopic makeup of the styrene product does not change over time in these experiments indicates that H/D exchange between benzene and carboxylic acid is not likely to occur. From these findings, it can be concluded that benzene C–H activation is likely irreversible under the reaction conditions.

**Figure 12:**
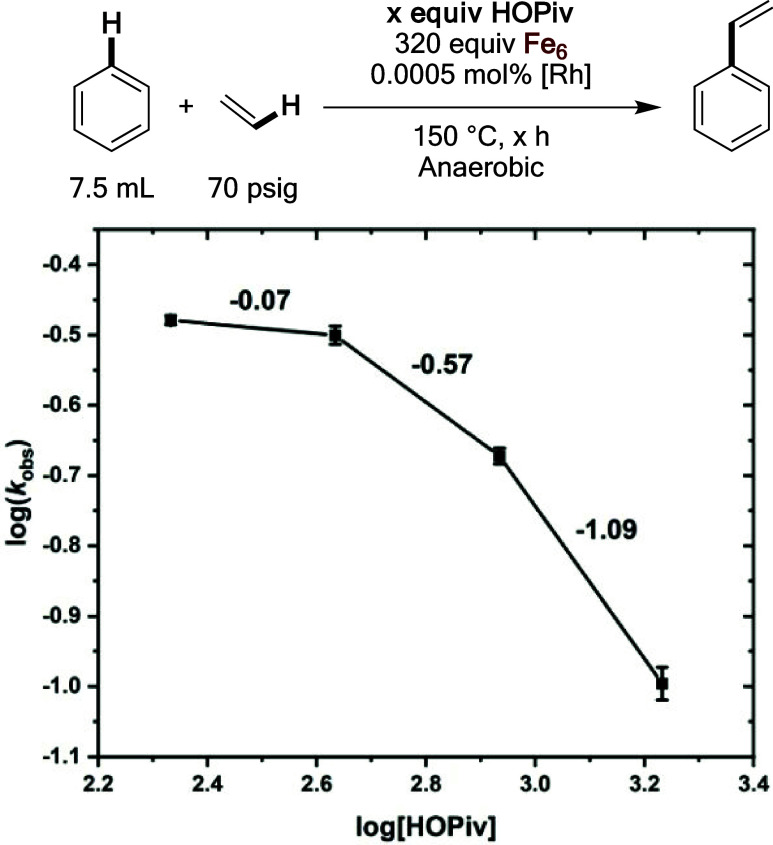
Log–log plot for the initial rate of benzene ethenylation under anaerobic conditions as a function of [HOPiv]. Reaction conditions: 7.5 mL of benzene, 0.0005 mol % (relative to benzene per single Rh atom) [(η^2^–C_2_H_4_)_2_Rh(μ–OAc)]_2_, × equiv of HOPiv (relative to Rh), 70 psig ethylene, 80 psig dinitrogen, 320 equiv of Fe_6_, 150 °C. Fe_6_ = Fe_6_^III^(μ–OH)_2_(μ_3_–O)_2_(μ–X)_12_(HX)_2_, which was prepared *in situ* by heating 0.81 mmol of Fe(OAc)_2_ and 1.62 mmol of HOPiv at 150 °C in 5 mL of benzene under 3 atm of dioxygen for 2 h. All data points reflect the average of a minimum of three independent reactions and error bars represent the standard deviation from the multiple independent experiments. The values shown to the right of the trendline are the slopes between the data points and are intended only to provide context for the dependence of the reaction rate on HOPiv concentration.

Next, we studied whether HOPiv inhibits the formation of Fe_6_^III^(μ–OH)_2_(μ_3_–O)_2_(μ–X)_12_(HX)_2_ by studying the effect of the HOPiv concentration on the rate of styrene production at anaerobic conditions. At anaerobic conditions, reoxidation of Fe to the active oxidant does not occur, so the influence of [HOPiv] on other steps in the catalytic cycle can be isolated from (1) the aerobic oxidation of reduced Fe by dioxygen and (2) oxidative decarboxylation of HOPiv. As shown in Figure 12, similar to aerobic conditions, a nonlinear inverse dependence on [HOPiv] is observed at anaerobic conditions. The consistent inverse dependence on [HOPiv] under aerobic and aerobic conditions indicates that HOPiv does not significantly inhibit the formation of Fe_6_^III^(μ–OH)_2_(μ_3_–O)_2_(μ–X)_12_(HX)_2_.

In summary, the inverse dependence on HOPiv is likely not attributable to (1) a reversible C–H activation step, (2) HOPiv inhibiting formation of Fe_6_^III^(μ–OH)_2_(μ_3_–O)_2_(μ–X)_12_(HX)_2_ or (3) HOPiv promoting catalyst deactivation (see the Supporting Information). Rather, we speculate that the inverse dependence of reaction rate on [HOPiv] can be attributed to HOPiv coordinating to Rh and inhibiting ethylene and/or benzene coordination. It is also possible that as [HOPiv] is increased, it induces a polarity change in the solvent that negatively affects the reaction rate.

### Effect of Ethylene Pressure

**Figure 13:**
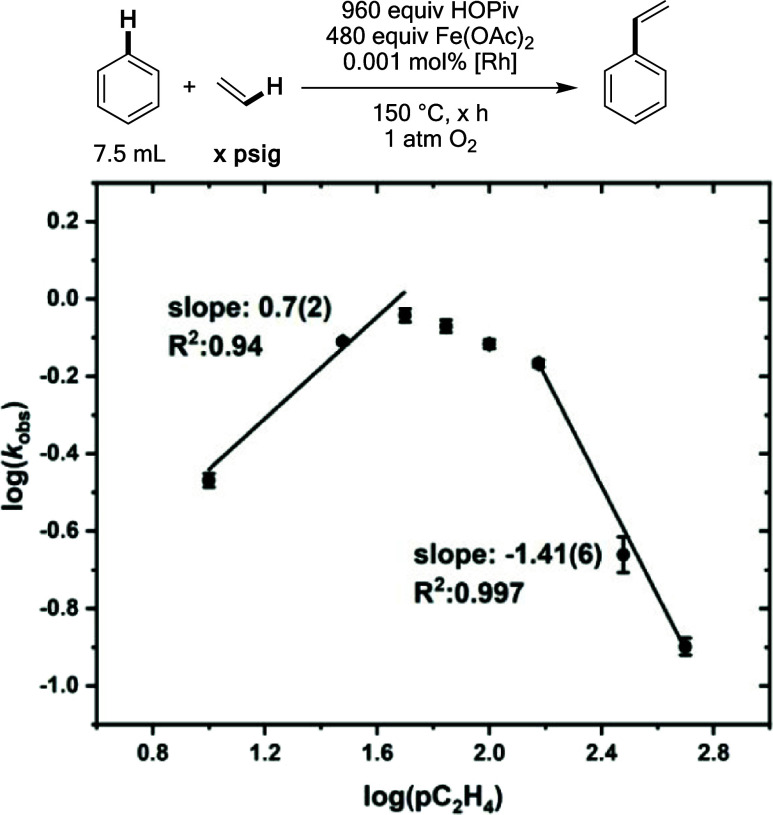
Log–log plot for the dependence of the initial benzene ethenylation reaction rate on ethylene pressure. Reaction conditions: 7.5 mL of benzene, 0.001 mol % (relative to benzene per single Rh atom) [(η^2^–C_2_H_4_)_2_Rh(μ–OAc)]_2_, 480 equiv of Fe(OAc)_2_ or Cu(OPiv)_2_, × mol % HOPiv, 70 psig ethylene, 1 atm dioxygen, 150 °C. All data points reflect the average of a minimum of three independent reactions, and error bars represent the standard deviation from the multiple independent experiments.

The dependence of the styrene formation rate on ethylene pressure was studied. An initial positive dependence on ethylene pressure was observed, followed by a sharp inverse dependence at ethylene pressures >150 psig (Figure 13). Inverse dependencies on olefin concentration have been observed previously with Ru, Ir, and Pt arene alkylation catalysts, which was attributed to the coordination of a second equiv of olefin with the olefin insertion product to inhibit arene coordination and C–H activation (graphical representation below).^39,49,54^ This contrasts with our previously reported results using Rh arene alkenylation catalysts for which first-order dependencies on ethylene concentration were observed under all conditions studied both with Cu(II) and dioxygen as the oxidant.^59,62^ In the present case, it is possible to envision ethylene coordination to a Rh-phenethyl intermediate inhibiting β-hydride elimination, and it is also possible that the inverse dependence on ethylene concentration results from ethylene coordination to a reduced Fe species, which might inhibit its reaction with dioxygen to form Fe_6_^III^(μ–OH)_2_(μ_3_–O)_2_(μ–X)_12_(HX)_2_ (see below for a graphical representation). We studied the possibility that a competitive ethylene functionalization reaction occurs at elevated ethylene pressure and observed small quantities of butadiene at 70 and 500 psig (4.9(5) and 3.7(4) TOs, respectively). These reactions were carried out in *tert*-butylbenzene rather than benzene to allow quantification of butadiene by condensing the reaction mixtures at −50 °C (Table S2). Although the ethylene oxidative hydroarylation reaction is more sensitive to ethylene pressure than the ethylene oxidative coupling reaction, the lack of an increase in butadiene production upon increasing the ethylene pressure suggests that competitive butadiene formation is not likely the origin of an inverse dependence on ethylene pressure. The presence of [(η^2^–C_2_H_4_)_2_Rh(μ–OAc)]_2_ was required in order for butadiene formation to occur, suggesting that butadiene is not formed by a Fe-catalyzed reaction (Table S2).

**Figure 14:**
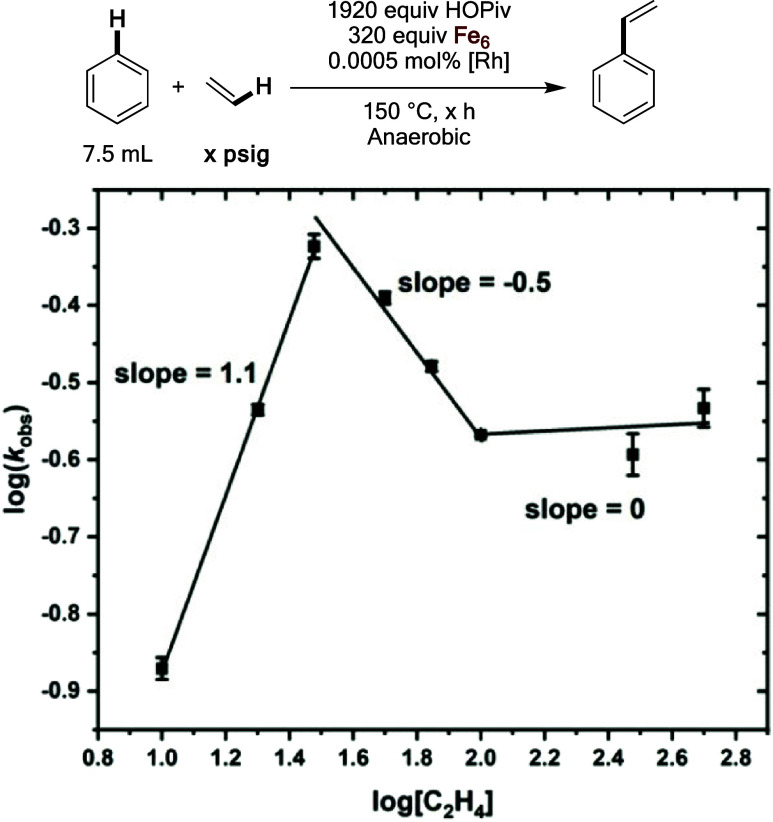
Log–log plot for the dependence on the initial benzene ethenylation rate as a function of ethylene pressure under anaerobic conditions. Reaction conditions: 7.5 mL of benzene, 0.0005 mol % (relative to benzene per single Rh atom) [(η^2^–C_2_H_4_)_2_Rh(μ–OAc)]_2_, 1920 equiv of HOPiv (relative to Rh), × psig ethylene, and at ethylene pressures <150 psig, dinitrogen pressure was added to bring the total pressure to 150 psig, 320 equiv of Fe_6_, 150 °C. Fe_6_ = Fe_6_^III^(μ–OH)_2_(μ_3_–O)_2_(μ–X)_12_(HX)_2_, which was prepared *in situ* by heating 0.81 mmol of Fe(OAc)_2_ and 1.62 mmol of HOPiv at 150 °C in 5 mL of benzene under 3 atm of dioxygen for 2 h. All data points reflect the average of a minimum of three independent reactions and error bars represent the standard deviation from the multiple independent experiments.

Similar to the experiments carried out with HOPiv, kinetic studies under anaerobic conditions were performed at varying ethylene pressure. Since reoxidation of Fe by dioxygen does not occur under anaerobic conditions, the effects of ethylene pressure on steps except for dioxygen activation can be isolated. The results in Figure 14 show a near first-order dependence on ethylene at low ethylene pressures, which is consistent with ethylene coordination and subsequent insertion occurring before or during the rate-limiting step. Notably, under aerobic conditions, the slope in the positive regime is <1, possibly indicating that inhibition effects of ethylene contribute to the initial less than first-order dependence. At ethylene pressures >30 psig, an inverse dependence on ethylene pressure is observed, which ultimately saturates. The observation of an inverse dependence suggests that ethylene coordination to a Rh-phenethyl intermediate to form an intermediate formulated as Rh(η^2^–C_2_H_4_)(CH_2_CH_2_Ph) might be occurring, as has been reported previously for Ru, Pt, and Ir catalysts for arene alkylation.^43,49,54^ There are two important differences in the inverse dependencies observed in aerobic and anaerobic conditions: (1) in anaerobic conditions, the slope in the inverse region is −0.5 while it is −1.4 in aerobic conditions, and (2) the inverse dependence saturates at anaerobic conditions but not at aerobic conditions. These findings suggest that in addition to ethylene coordination to Rh to form off-cycle Rh(η^2^–C_2_H_4_)(CH_2_CH_2_Ph) intermediates, ethylene also likely coordinates to reduced Fe species to inhibit its reoxidation to Fe_6_^III^(μ–OH)_2_(μ_3_–O)_2_(μ–X)_12_(HX)_2_ by dioxygen.

**Scheme 6:**
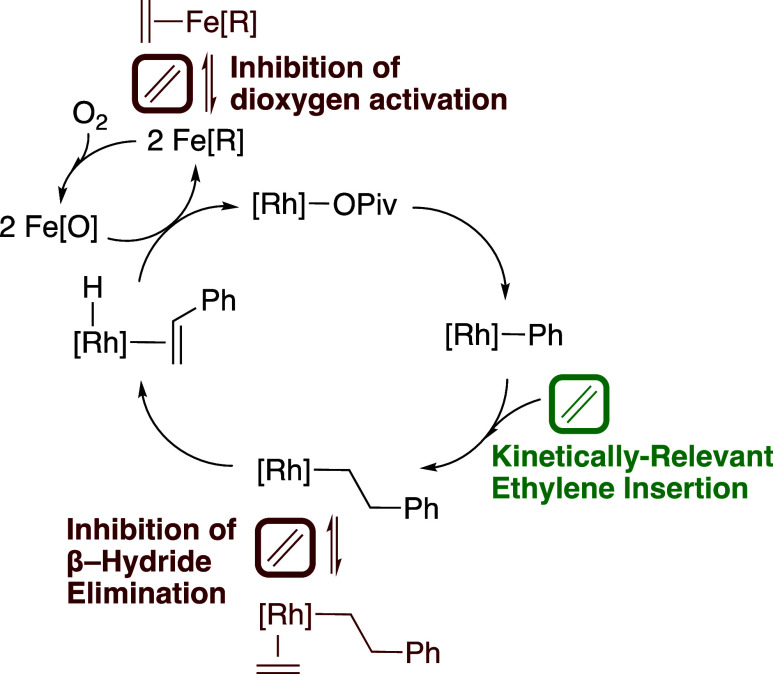
Simplified Representation of the Possible Origins of the Observed Complicated Dependence on Ethylene Pressure Fe[O] represents the oxidized form of the Fe-based oxidant and Fe[R] represents its reduced form.

From the studies of ethylene pressure effects, it is proposed that the following three factors contribute to the complicated ethylene pressure dependence (Scheme 6): (1) ethylene coordination and insertion occurs before the rate-limiting step in the Rh cycle, resulting in a first-order contribution, (2) ethylene coordinates with a Rh-phenethyl intermediate to form Rh(η^2^–C_2_H_4_)(CH_2_CH_2_Ph), which is an off-cycle intermediate, and (3) ethylene coordinates with the reduced form of the Fe-based oxidant to form an ethylene-coordinated off-cycle intermediate, which decreases the concentration of Fe_6_^III^(μ–OH)_2_(μ_3_–O)_2_(μ–X)_12_(HX)_2_, the reaction of which has a near first-order dependence in (see below).

### Kinetic Dependence on the Oxidant

**Figure 15:**
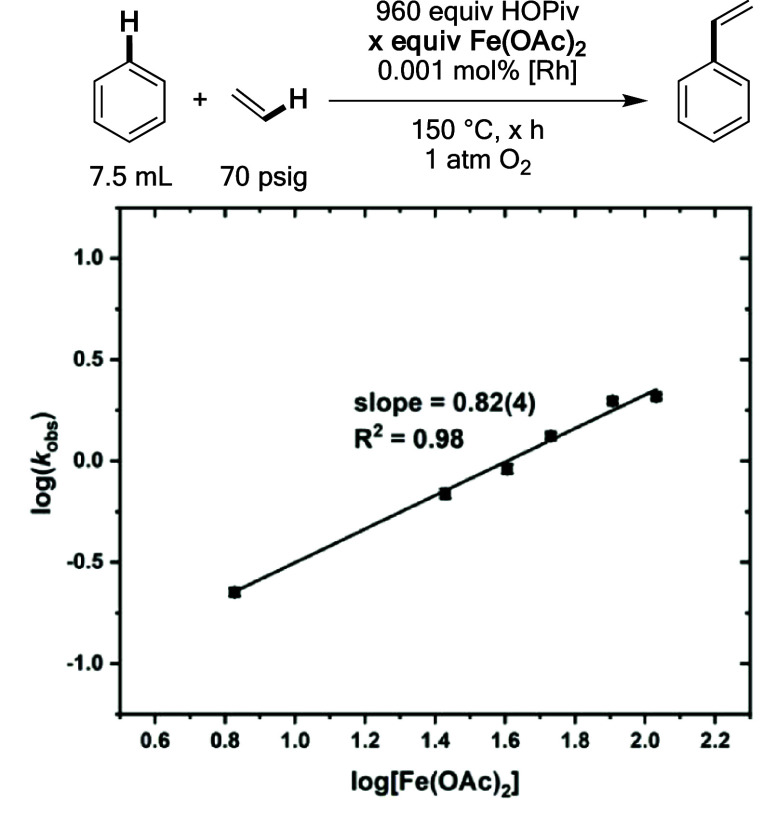
Log–log plot for the dependence of the initial benzene ethenylation reaction rate on the concentration of Fe(OAc)_2_. Reaction conditions: 7.5 mL of benzene, 0.001 mol % (relative to benzene per single Rh atom) [(η^2^–C_2_H_4_)_2_Rh(μ–OAc)]_2_, × equiv of Fe(OAc)_2_ or Cu(OPiv)_2_, 960 equiv of HOPiv, 70 psig ethylene, 1 atm dioxygen, 150 °C. All data points reflect the average of a minimum of three independent reactions, and error bars represent the standard deviation from the multiple independent experiments.

Next, the influence of the Fe carboxylate concentration and dioxygen pressure was probed. With respect to the concentration of Fe(OAc)_2_, an order of 0.82(4) was observed, which is slightly less than a first-order dependence (Figure 15). This result suggests that the oxidation of Rh–H by a Fe-based species is likely kinetically relevant. The slightly less than first-order dependence might be attributable to an undesired competitive oxidation of Rh to an off-cycle intermediate. Previously, our group reported a near zero-order, but slightly inverse dependence on the concentration of Cu(OPiv)_2_ under anaerobic conditions, and speculated that higher Cu(OPiv)_2_ concentrations might result in undesired Rh oxidation.^62^

**Figure 16:**
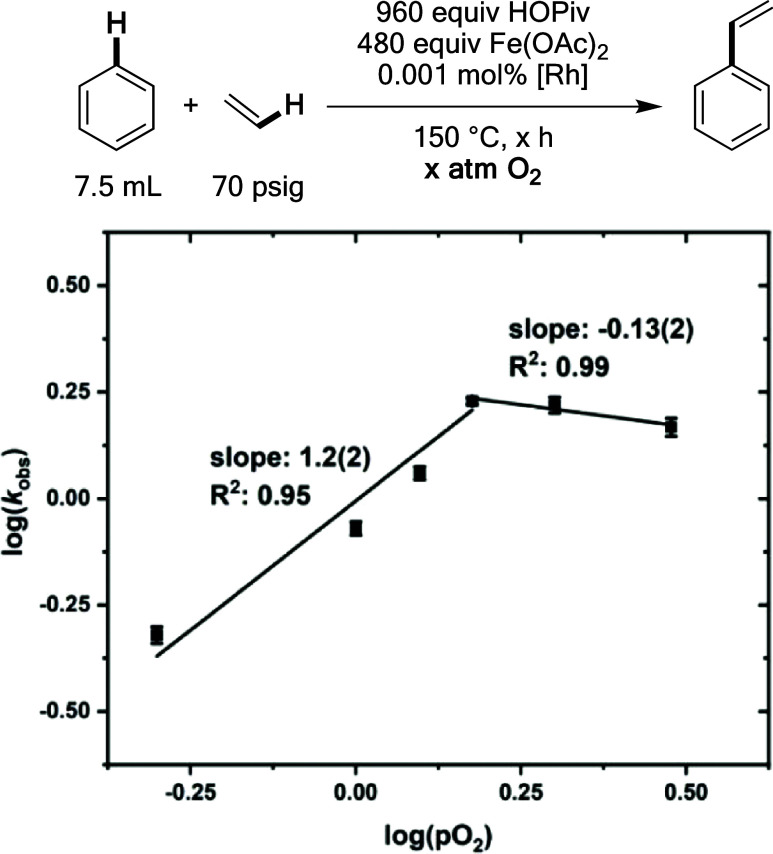
Log–log plot for the dependence of the initial benzene ethenylation reaction rate on dioxygen pressure. Reaction conditions: 7.5 mL of benzene, 0.001 mol % (relative to benzene per single Rh atom) [(η^2^–C_2_H_4_)_2_Rh(μ–OAc)]_2_, 480 equiv of Fe(OAc)_2_, 960 equiv of HOPiv, 70 psig ethylene, × atm dioxygen, 150 °C. All data points reflect the average of a minimum of three independent reactions and error bars represent the standard deviation from the multiple independent experiments.

Saturation kinetics after an initial first-order dependence in dioxygen pressure were measured (Figure 16). As discussed above, Rh–H oxidation by Fe_6_^III^(μ–OH)_2_(μ_3_–O)_2_(μ–X)_12_(HX)_2_ is likely kinetically relevant because a near first-order dependence on its concentration was observed. The observation of an initial first-order dependence on the dioxygen concentration suggests that the rate of Fe_6_^III^(μ–OH)_2_(μ_3_–O)_2_(μ–X)_12_(HX)_2_ formation has a first-order dependence on the dioxygen concentration.

### Proposed Reaction Mechanism

**Scheme 7:**
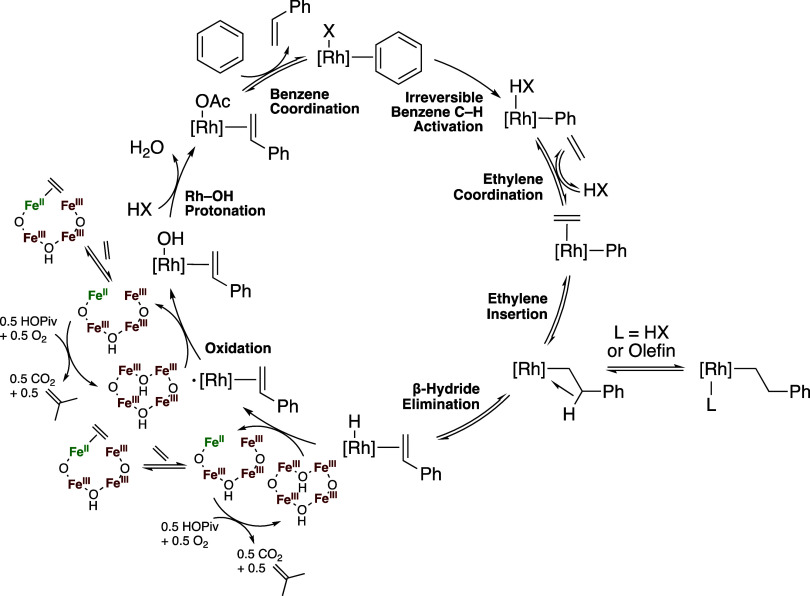
Proposed Mechanism of Aerobic Benzene Ethenylation

From the results of Fe speciation studies and mechanistic studies of aerobic and anaerobic benzene ethenylation, the mechanism shown in Scheme 7 is proposed. An Rh–X (X = OAc or OPiv) intermediate performs irreversible benzene C–H activation, forming an Rh(Ph)(HOPiv) intermediate. HOPiv is subsequently reversibly displaced by ethylene, and ethylene insertion into the Rh–Ph bond occurs. HX or a second equivalent of ethylene can coordinate with the formed Rh(CH_2_CH_2_Ph) intermediate, resulting in an equilibrium with off-cycle Rh(L)(CH_2_CH_2_Ph) (L = C_2_H_4_ or HX). Following β-hydride elimination, oxidation of Rh–H by two equiv of Fe_6_^III^(μ–OH)_2_(μ_3_–O)_2_(μ–X)_12_(HX)_2_ occurs, possibly by the mechanism detailed in Scheme 4 earlier in the manuscript. The reduced form of the Fe-based oxidant is then reoxidized to Fe_6_^III^(μ–OH)_2_(μ_3_–O)_2_(μ–X)_12_(HX)_2_ by dioxygen in a kinetically relevant step. Coordination of ethylene to the reduced form of the oxidant likely occurs, which inhibits its reaction with dioxygen to form Fe_6_^III^(μ–OH)_2_(μ_3_–O)_2_(μ–X)_12_(HX)_2_. The mechanism of Fe_6_^III^(μ–OH)_2_(μ_3_–O)_2_(μ–X)_12_(HX)_2_ formation is discussed in detail in Scheme 3 above.

### Studies of Catalyst Longevity under Aerobic Conditions

**Figure 17:**
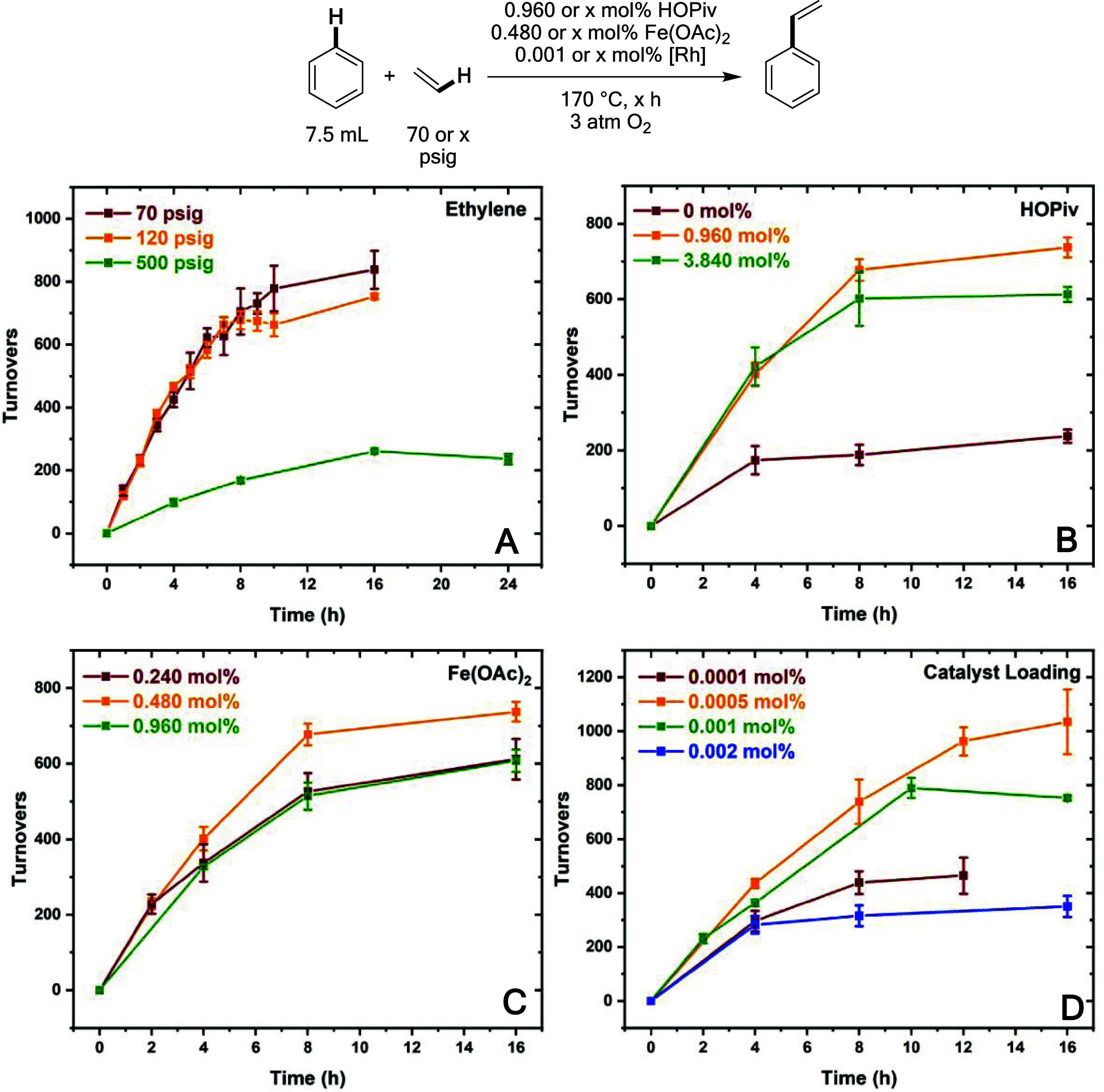
TOs versus time plots with varying [Rh], [ethylene], [HOPiv], and [Fe(OAc)_2_]. Reaction conditions: 7.5 mL of benzene, 0.001 or × mol % (relative to benzene per single Rh atom) [(η^2^–C_2_H_4_)_2_Rh(μ–OAc)]_2_, 0.480 or × mol % (relative to a single Rh atom) Fe(OAc)_2_, 0.960 or × mol % HOPiv, 70 or × psig ethylene, 3 atm dioxygen, 170 °C. All data points reflect the average of a minimum of three independent reactions, and error bars represent the standard deviation from the multiple independent experiments.

Studies of catalyst longevity were performed at 170 °C to identify factors that lead to catalyst deactivation and to further optimize the reaction conditions. We studied whether the concentrations of ethylene, HOPiv, Fe(OAc)_2_, and [(η^2^–C_2_H_4_)_2_Rh(μ–OAc)]_2_ impact catalyst longevity. Mechanistic studies indicated a complicated dependence of reaction rate on ethylene pressure at 150 °C, which reaches a maximum initial rate with 50 psig of ethylene (see above). Accordingly, the effect of ethylene pressure on the initial reaction rate and the longevity of catalysis was probed. As shown in Figure 17A, statistically identical initial reaction rates and turnover numbers were observed in the presence 70 and 120 psig of ethylene, suggesting that these ethylene pressures result in ethylene concentrations near the optimal value. Consistent with mechanistic studies discussed above, with 500 psig of ethylene, a slower initial rate is observed, and apparent catalyst deactivation occurs at a similar time to the reaction with 70 and 120 psig of ethylene. Next, we studied whether HOPiv concentration impacts catalyst longevity (Figure 17B). Previously, it was found that the presence of excess carboxylic acid is beneficial for aerobic reactions with Cu(II) carboxylate oxidants since carboxylic acids likely inhibit the reaction of Cu(II) carboxylate with water to form CuO.^77^ Additionally, the proposed mechanism of Fe_6_^III^(μ–OH)_2_(μ_3_–O)_2_(μ–X)_12_(HX)_2_ involves HOPiv oxidative decarboxylation; therefore, its presence is likely required for long-lived catalysis. As shown in Figure 17B, with no HOPiv present, limited catalysis occurs, suggesting that the formation of Fe_6_^III^(μ–OH)_2_(μ_3_–O)_2_(μ–X)_12_(HX)_2_ occurs by oxidative decarboxylation of OPiv ligands, which are depleted over time, or possibly that a different species is serving as the oxidant. With HOPiv loadings of 0.960 and 3.840 mol %, similar initial rates were observed, and slightly more TOs were achieved with an HOPiv loading of 0.960 mol %. Next, the effect of Fe(OAc)_2_ loading was studied (Figure 17C). It was found that slightly more TOs were achieved with a 0.480 mol % loading of Fe(OAc)_2_ than with loadings of 0.240 and 0.960 mol %. When varying the catalyst loading, limited catalyst longevity was observed with a loading of 0.0001 mol %. With 0.0005 mol % [Rh], slightly more TOs were observed after 16 h than with a loading of 0.001 mol % [Rh], and with a loading of 0.002 mol %, the TOs after 16 h are roughly half of those achieved with a loading of 0.001 mol % (Figure 17D). The observation that higher catalyst loadings give lower TOs at deactivation suggests that the concentration of styrene, benzaldehyde, and water that form might be factors that induce a halting of catalysis. We studied the effect of styrene, benzaldehyde, and water on the reaction and found that water and benzaldehyde have negligible effects on the reaction rate, while the addition of styrene substantially inhibits the reaction (Figure S27).

**Figure 18:**
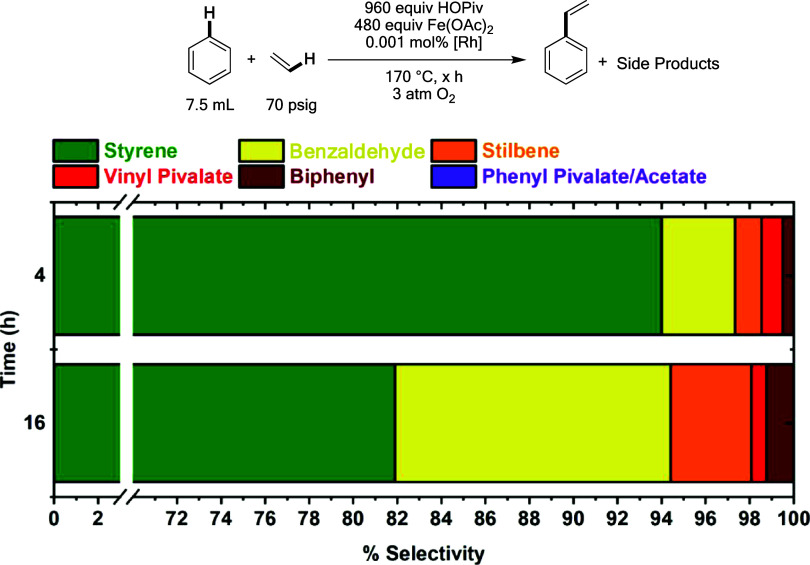
Selectivity for styrene versus side products at 4 and 16 h. Reaction conditions: 7.5 mL of benzene, 0.001 mol % (relative to benzene per single Rh atom) [(η^2^–C_2_H_4_)_2_Rh(μ–OAc)]_2_, 480 equiv (relative to single Rh atom) of Fe(OAc)_2_, 960 equiv of HOPiv, 70 psig ethylene, 3 atm dioxygen, 170 °C. The data reflect the average of a minimum of three independent reactions.

As demonstrated in Figure 18, the selectivity for styrene versus side products deteriorates over time for aerobic benzene ethenylation reactions performed at 170 °C. While the percentage of biphenyl and vinyl pivalate remains roughly constant and minimal between four and 16 h, substantial quantities of benzaldehyde and *trans*-stilbene are detected after 16 h. Benzaldehyde was previously found to form under aerobic conditions by oxidative cleavage of styrene by dioxygen,^59^ which does not require a catalyst to occur, although it was found that Fe and Rh additives accelerate this reaction (Figure S28). The formation of *trans*-stilbene as a result of styrene oxidative hydrophenylation can likely be suppressed by increasing the ethylene pressure; however, the reaction rate has an inverse dependence on ethylene pressure (see above). Since both benzaldehyde and *trans*-stilbene form as a result of styrene undergoing subsequent reactions, designing a process that continuously removes styrene would be necessary to achieve >95% selectivity for styrene. Neither phenyl pivalate nor phenyl acetate were detected by GC-MS, which, as discussed earlier in the manuscript, is a potential advantage of catalysis with Fe additives versus Cu additives.

### Studies of Anaerobic Benzene Ethenylation

**Figure 19:**
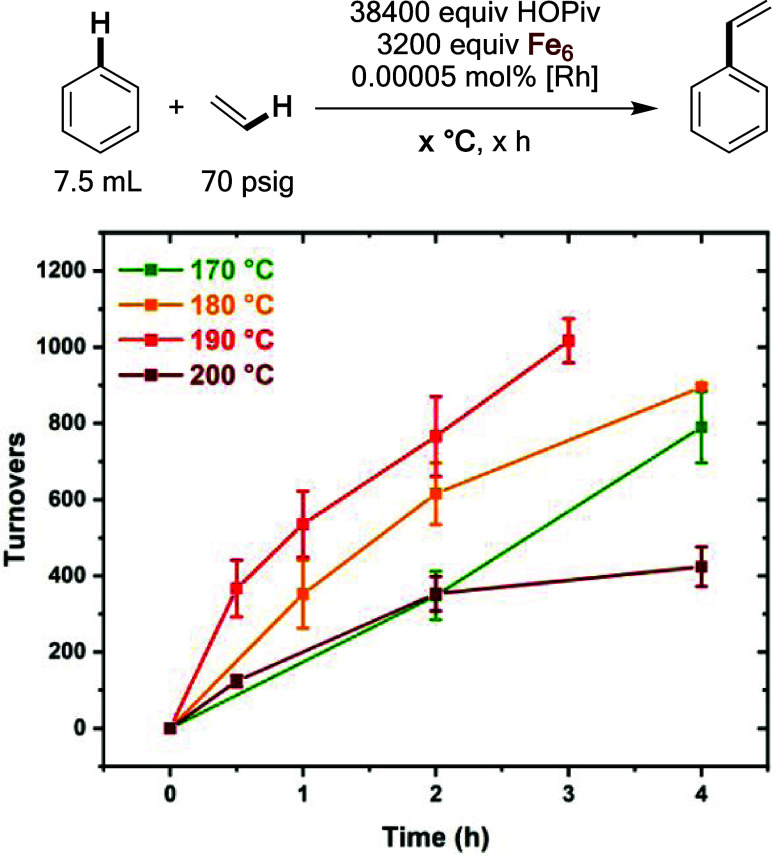
Initial kinetics of anaerobic benzene ethenylation as a function of the reaction temperature. Reaction conditions: 7.5 mL of benzene, 0.00005 mol % (relative to benzene per single Rh atom) [(η^2^–C_2_H_4_)_2_Rh(μ–OAc)]_2_, 3200 equiv of Fe_6_, 38 400 equiv of HOPiv, 70 psig ethylene, 80 psig dinitrogen, 170, 180, 190, or 200 °C. Fe_6_ = Fe_6_^III^(μ–OH)_2_(μ_3_–O)_2_(μ–X)_12_(HX)_2_ which was prepared *in situ* by heating 0.81 mmol of Fe(OAc)_2_ and 1.62 mmol of HOPiv at 170 °C in 5 mL of benzene under 3 atm of dioxygen for 2 h. All data points reflect the average of a minimum of three independent reactions and error bars represent the standard deviation from the multiple independent experiments.

To potentially avoid the production of significant benzaldehyde side products at extended reaction times, we performed reaction condition optimization under anaerobic conditions (see the Supporting Information). At optimized conditions, an initial TOF of ∼0.05 s^–1^ was achieved at 170 °C with a quantitative yield of styrene relative to the hexanuclear oxidant. Next, the kinetics of the reaction as a function of temperature were studied (Figure 19). It was found that the initial turnover frequency roughly doubles each ten-degree increment from 170 to 190 °C, however, a slower turnover frequency is observed at 200 °C. The observation of decreased turnover frequency at 200 °C might be attributable to deactivation of the Rh catalyst or of the Fe oxidant, or the result of thermal decomposition of OPiv functional groups.^78^ Reactions at otherwise identical conditions utilizing HOAc or HOiBu additives, which might impart greater thermal stability, resulted in substantially slower catalysis than when the HOPiv additive is used (Figure S31).

**Figure 20:**
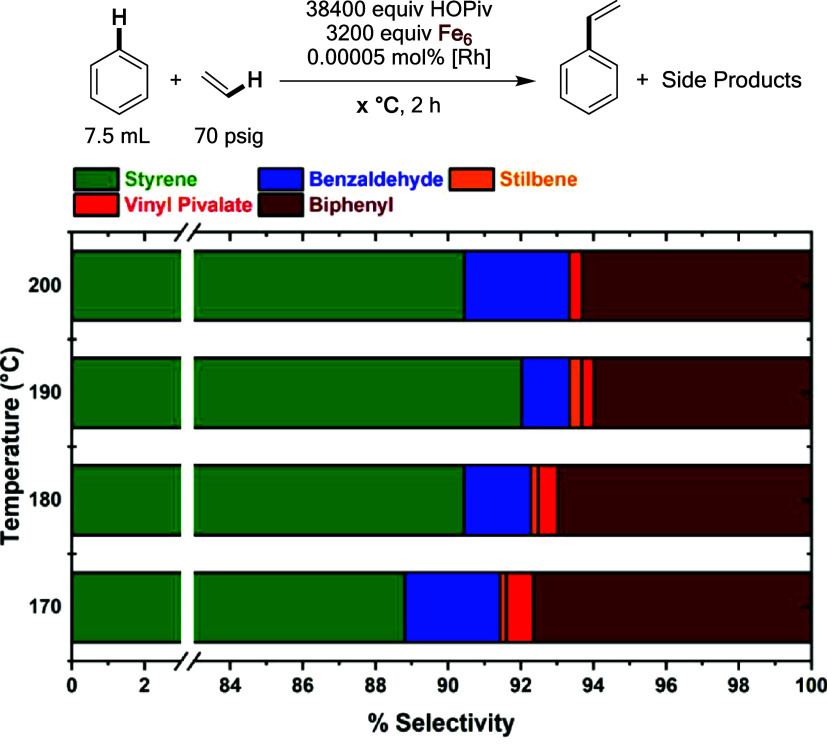
Selectivity for styrene versus side products after 2 h for anaerobic benzene ethenylation reactions performed at varying temperatures. Reaction conditions: 7.5 mL benzene, 0.00005 mol % (relative to benzene per single Rh atom) [(η^2^–C_2_H_4_)_2_Rh(μ–OAc)]_2_, 3200 equiv of Fe_6_, 38 400 equiv of HOPiv, 70 psig ethylene, 80 psig dinitrogen, 170, 180, 190, or 200 °C. Fe_6_ = Fe_6_^III^(μ–OH)_2_(μ_3_–O)_2_(μ–X)_12_(HX)_2_ which was prepared *in situ* by heating 0.81 mmol of Fe(OAc)_2_ and 1.62 mmol of HOPiv at 170 °C in 5 mL of benzene under 3 atm of dioxygen for 2 h. All data points reflect the average of three independent reactions.

As shown in Figure 20, benzene ethenylation reactions performed under anaerobic conditions yield product distributions similar to those performed under aerobic conditions (see above). The two most significant differences are that biphenyl is a more significant side product under anaerobic conditions than in aerobic conditions, and benzaldehyde is a less significant side product under anaerobic conditions than in aerobic conditions. Although less stilbene is observed at anaerobic conditions, this is likely the result of using a 20-fold lower catalyst loading under anaerobic conditions than in aerobic conditions, resulting in a lower concentration of styrene to compete with ethylene as a substrate for oxidative hydrophenylation. The observation of benzaldehyde under anaerobic conditions suggests that Fe_6_^III^(μ–OH)_2_(μ_3_–O)_2_(μ–X)_12_(HX)_2_ is capable of oxidatively cleaving styrene in the absence of dioxygen.

**Figure 21:**
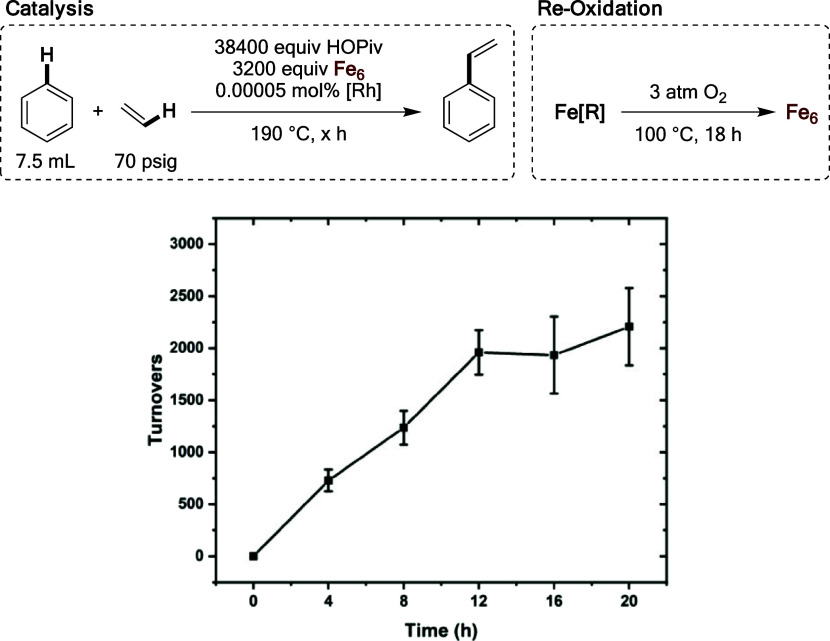
Turnovers versus time plot for anaerobic benzene ethenylation reactions in which a reoxidation of the reduced Fe material was performed after every 4 h of reaction time (after each time point shown in the plot). Reaction conditions: 7.5 mL of benzene, 0.00005 mol % (relative to benzene per single Rh atom) [(η^2^–C_2_H_4_)_2_Rh(μ–OAc)]_2_, 3200 equiv of Fe_6_ (relative to Rh), 38 400 equiv of HOPiv, 70 psig of ethylene, 80 psig of dinitrogen, 190 °C. Dioxygen was purged out of each reactor prior to benzene ethenylation. Fe_6_ = Fe_6_^III^(μ–OH)_2_(μ_3_–O)_2_(μ–X)_12_(HX)_2_, which was prepared *in situ* by heating 0.81 mmol of Fe(OAc)_2_ and 1.62 mmol of HOPiv at 170 °C in 5 mL of benzene under 3 atm of dioxygen for 2 h. For Fe reoxidation reactions, reactors were pressurized with 3 atm of dioxygen and heated at 100 °C for 18 h. All data points reflect the average of a minimum of three independent reactions.

Having identified 190 °C as the temperature at which turnover frequency is maximized, we studied the possibility of reoxidizing the reduced form of the oxidant in a step separate from catalysis. Since the reaction rate is dependent upon the concentration of Fe_6_^III^(μ–OH)_2_(μ_3_–O)_2_(μ–X)_12_(HX)_2_, and slows over time as a result of this, we speculated that performing dioxygen reoxidations at approximately 50% conversion of Fe_6_^III^(μ–OH)_2_(μ_3_–O)_2_(μ–X)_12_(HX)_2_ might result in prolonged catalysis at faster rates. Accordingly, reaction mixtures were heated at 100 °C under 3 atm of dioxygen for 18 h after every 4 h of benzene ethenylation reaction time. As shown in Figure 21, near linearity in the turnovers versus time plot is observed for 12 h, and ∼2000 turnovers are achieved prior to apparent catalyst deactivation. This contrasts with reactions in which dioxygen reoxidations are not performed, for which a decrease in reaction rate is observed over time as a result of Fe_6_^III^(μ–OH)_2_(μ_3_–O)_2_(μ–X)_12_(HX)_2_ consumption. This experiment demonstrates the ability to regenerate Fe_6_^III^(μ −OH)_2_(μ_3_–O)_2_(μ–X)_12_(HX)_2_ from its reduced form and dioxygen.

## Summary and Conclusions

Rh-catalyzed aerobic and anaerobic production of styrene from benzene and ethylene with Fe_6_^III^(μ–OH)_2_(μ_3_–O)_2_(μ–X)_12_(HX)_2_ (X = carboxylate) as the likely direct oxidant has been demonstrated. At optimized conditions, a turnover frequency of ∼0.2 s^–1^ is demonstrated, with ∼92% selectivity for styrene. Given the earth abundance of Fe and the activity of Fe_6_^III^(μ–OH)_2_(μ_3_–O)_2_(μ–X)_12_(HX)_2_ as an easily accessible and dioxygen-recyclable oxidant, the use of Fe_6_^III^(μ–OH)_2_(μ_3_–O)_2_(μ–X)_12_(HX)_2_ and related complexes for other hydrocarbon oxidative functionalization reactions could be of interest.

Primary conclusions from our studies include:

1. The proposed mechanism of Fe_6_^III^(μ–OH)_2_(μ_3_–O)_2_(μ–X)_12_(HX)_2_ formation from FeX_2_ and HX involves the oxidation of 6 equiv of FeX_2_ by dioxygen to form two equiv of Fe^II^Fe_2_^III^(μ_3_–O)(μ–X)_6_(HX)_3_. Two equiv of Fe^II^Fe_2_^III^(μ_3_–O)(μ–X)_6_(HX)_3_ then undergo the reaction with one equiv of dioxygen to form Fe_6_^III^(μ_4_–O_2_)(μ_3_–O)_2_(μ–X)_12_(HX)_2_, which oxidatively decarboxylates carboxylic acid to form Fe_6_^III^(μ–OH)_2_(μ_3_–O)_2_(μ–X)_12_(HX)_2_.
2. Rh catalyst longevity and turnover frequency are sensitive to carboxylate identity: optimal conditions are achieved with the combination of pivalate and acetate-based Fe(II) carboxylates and carboxylic acids.
3. Mechanistic studies indicate irreversible benzene C–H activation, kinetically relevant ethylene insertion into the Rh–Ph bond, β-hydride elimination, and kinetically relevant oxidation of the formed Rh–H intermediate by two equiv of Fe_6_^III^(μ–OH)_2_(μ_3_–O)_2_(μ–X)_12_(HX)_2_. The reduced form of the oxidant is reoxidized by dioxygen (when present) in a kinetically relevant step. High concentrations of ethylene inhibit the reaction, likely by coordination with RhCH_2_CH_2_Ph to form Rh(η^2^–C_2_H_4_)CH_2_CH_2_Ph, and also by coordination with the reduced form of the Fe-based oxidant, which likely inhibits dioxygen activation.
4. The reaction at aerobic conditions is substantially inhibited by styrene, and substantial quantities of benzaldehyde and *trans*-stilbene form over time as the result of styrene oxidative cleavage and oxidative hydrophenylation, respectively.
5. Fe_6_^III^(μ_4_–O_2_)(μ_3_–O)_2_(μ–X)_12_(HX)_2_ is active as an oxidant under anaerobic conditions, yielding higher selectivity for styrene than was observed under aerobic conditions, and a turnover frequency of ∼0.2 s^–1^. The reduced form of the oxidant can be reoxidized to Fe_6_^III^(μ_4_–O_2_)(μ_3_–O)_2_(μ–X)_12_(HX)_2_ by dioxygen at 100 °C.

## Methods

### Experimental Methods

#### General Considerations

Unless otherwise noted, all synthetic procedures were performed under anaerobic conditions in a dinitrogen-filled glovebox or by using standard Schlenk techniques. Glovebox purity was maintained by periodic dinitrogen purges and was monitored using an oxygen analyzer (O_2_ < 15 ppm for all reactions). Benzene and pentanes were obtained from commercial sources and purified using a solvent purification system with activated alumina. [(η^2^–C_2_H_4_)_2_Rh(μ–OAc)]_2_,^90^ Fe(TFA)_3_,^17^ Fe(OPiv)_2_,^91^ [Fe_3_^III^(μ_3_–O)(μ–OPiv)_6_(H_2_O)_3_][OPiv],^18^ Fe_2_^III^Fe^II^(μ_3_–O)(μ–OPiv)_6_(HOPiv)_3_,^92^ and Fe_6_^III^(μ_4_–O_2_)(μ_3_–O)_2_(μ–OPiv)_12_(HOPiv)_2_^26^ were prepared according to published procedures. All other reagents were obtained from commercial sources and used as received. Quantities of [(η^2^–C_2_H_4_)_2_Rh(μ–OAc)]_2_ of less than 1 mg were obtained by dilution in benzene solutions. CAUTION: ethylene/dioxygen mixtures can be explosive. Care should be taken to avoid sparks, and reactions should be performed in stainless steel reactors with pressure ratings higher than the pressure that would be reached in the event of ignition equipped with pressure relief valves. While heating and pressurizing the vessels, they should be kept behind a blast shield in a fume hood to protect the operator in the event the pressure relief valve opens. GC-MS analysis was performed using a Shimadzu GCMS-QP-2030 Plus system with a 30 m × 0.25 mm RTX-Rxi-5 ms column with 0.25 μM film thickness. A plot of peak area versus molar ratio gave a regression line using hexamethylbenzene as the internal standard. The slope and correlation coefficient of the regression lines were 0.394 and 0.999 (styrene), 0.238 and 0.993 (vinyl pivalate), 0.232 and 0.999 (benzaldehyde), 0.964 and 0.999 (biphenyl), 0.679 and 0.999 (*trans*-stilbene), and 0.763 and 0.999 (4-*tert*-butylstyrene). Quantification of butadiene production was carried out on a Shimadzu GCMS-QP-2030 Plus system with a 30 m × 0.25 mm RT-Q-Bond capillary column with an 8 μM film thickness using tetrahydrofuran (THF) as an external standard. The slope and correlation coefficient for butadiene were 0.470 and 0.999.

#### Synthesis of Fe_6_^III^(μ–OH)_2_(μ_3_–O)_2_(μ–OPiv)_12_(HOPiv)_2_

A solid sample of Fe_2_^III^Fe^II^(μ_3_–O)(μ–OPiv)_6_(HOPiv)_3_ (32.25 g, 29.8 mmol) was allowed to sit in air for 1 month, and a color change from black to dark orange was observed. The sample was dissolved in pentane and filtered through a Celite-loaded fine porosity frit. X-ray quality crystals of Fe_6_(μ–OH)_2_(μ_3_–O)_2_(μ–OPiv)_12_(HOPiv)_2_·C_5_H_12_ formed upon removal of pentane *in vacuo*. Excess HOPiv and the pentane solvate were removed by heating *in vacuo* at 70 °C for 24 h. The isolated yield was 66% (18.02 g). Anal. Calcd for Fe_6_O_32_C_70_H_130_: C, 46.23; H, 7.20. Found (triplicate): C, 45.9(1); H, 7.20(7).

#### General Procedure for Aerobic Benzene Ethenylation

Under an atmosphere of dry dinitrogen, three 10 mL vials with stir bars were charged with 7.5 mL (84.6 mmol) of benzene, [(η^2^–C_2_H_4_)_2_Rh(μ–OAc)]_2_ (0.001 mol % relative to benzene per single Rh atom, 0.184 mg, 0.846 μmol), Fe(OAc)_2_ (69.9 mg, 0.406 mmol), and HOPiv (82.9 mg, 0.812 mmol). The vials were inserted into previously described stainless steel reactors. The headspace of the stainless steel reactors was flushed with dioxygen by pressurizing with 15 psig of dioxygen and releasing the pressure six times, leaving 1 atm (0 psig) of dioxygen in the reactors. The reactors were subsequently pressurized with 70 psig of ethylene and heated in an aluminum block on a hot plate at 150 °C. Upon cooling to room temperature, reactors were sampled in air every 2 h using a long needle. 50 μL aliquots of the reaction mixtures were combined with 50 μL of a 111 mM hexamethylbenzene benzene solution in 0.25 mL of benzene to give 100 equiv of external standard hexamethylbenzene. The benzene solutions were washed with a saturated aqueous solution of NaOH (1.5 mL) to remove Fe complexes and HOPiv, and the organic layer was analyzed by GC-MS.

#### General Procedure for Anaerobic Benzene Ethenylation

Under an atmosphere of dry dinitrogen, three 10 mL vials with stir bars were charged with 7.5 mL (84.6 mmol) of benzene, Fe(OAc)_2_ (139.7 mg, 0.812 mmol), and HOPiv (165.2 mg, 1.62 mmol). The vials were inserted into previously described stainless steel reactors. The reactors were subsequently pressurized with 70 psig of dinitrogen and 45 psig of dioxygen and heated in an aluminum block on a hot plate at 150 °C for 2 h. The quantity of [(η^2^–C_2_H_4_)_2_Rh(μ–OAc)]_2_ for three reactions (0.0005 mol % relative to benzene per single Rh atom, 0.184 mg, 0.846 μmol) was added to 7.5 mL of benzene, and 2.5 mL aliquots of this solution were added to each of the three stainless steel reactors. Dioxygen was removed from the reactors by cycling between dinitrogen pressure and partial vacuum on a high-pressure line, and the reactors were subsequently pressurized with 70 psig of ethylene. The reactors were heated in an aluminum block on a hot plate for 30 min intervals. 50 μL aliquots of the reaction mixtures were combined with 50 μL of an 11.1 mM benzene solution of hexamethylbenzene in 0.25 mL of benzene to give 200 equiv of external standard hexamethylbenzene. The benzene solutions were washed with a saturated aqueous solution of NaOH (1.5 mL) to remove Fe salts and carboxylic acid, and the organic layer was analyzed by GC-MS.

### Computational Methods

All density functional theory calculations were performed using Jaguar^93^ v11.7 (Schrodinger Inc.). The B3LYP^94^ hybrid GGA functional was used for all calculations, along with the D3^95^ empirical correction for London Dispersion forces. All atoms were described using the 6-311G^96^ basis set augmented with polarization and diffuse functions, with the exception of Fe which was described by the Los Alamos effective small-core potential^97,98^ (designated LACV3P**++ in Jaguar), which includes explicit (3s)^2^(3p)^6^ core electrons. All calculations used implicit solvation as implemented by Jaguar’s Poisson–Boltzmann Finite element (PBF)^99^ solvation model, which includes the effect of polarization of the implicit solvation interacting with the explicit electrons. Solvent parameters matching benzene were used (ε = xx, radius = xx).

Frequency calculations were performed to confirm local minima (no negative curvatures) and to predict thermochemical properties such as zero-point energies (ZPE), enthalpy correction from 0K to operating temperature, and entropies. To correct for librational modes that are hindered by solution, we reduce translational and rotational entropies by 50%. That is, Gibbs free energy (*G*) is computed as *G* = *H* – T*(0.5*(*S*_trans_ + *S*_rot_) + *S*_vib_ + *S*_elec_), where *H* is enthalpy (including ZPE), and *S*_trans_, *S*_rot_, *S*_vib_, and *S*_elec_ are the translational, rotational, vibrational, and electronic entropies, respectively.

DFT is a single-determinant method such that antiferromagnetic spin states are not correctly captured. Because of this, all complexes were computed in their high-spin configurations (i.e., all spins aligned). Thus, Fe(II), Fe(III), and Fe(IV) were treated as quintet (*S* = 2), sextet, and quintet, respectively. Lower spin states were also computed but were found to be higher in energy in all cases. We do not expect the O–H bond energies to depend on whether the carboxylates are pivalates or acetates, so we used acetates to reduce computational expense. To further save on computational expense, we removed carboxylic acids from our calculations since we do not expect their presence to change O–H bond energies. We assumed 1/2 H_2_ as the hydrogen reference since we do not know the exact form of the Rh–H species. This is acceptable since we are interested in the trend of the O–H bond energies rather than the absolute values.

## Supporting Information Available

Additional experimental details including detailed experimental procedures and data used to generate kinetic plots (PDF)

## Accession Codes

CCDC 2336484–2336485 contains the supplementary crystallographic data for this paper. These data can be obtained free of charge from the Cambridge Crystallographic Data Centre via https://www.ccdc.cam.ac.uk/structures/
