# Supplementary material for: Hexa-Fe(III) Carboxylate Complexes Facilitate Aerobic Hydrocarbon Oxidative Functionalization: Rh Catalyzed Oxidative Coupling of Benzene and Ethylene to Form Styrene
Source: ACS Catal. 2024 Jun 24;14(13):10295–316. doi: 10.1021/acscatal.4c02355 (PMC11232027; doi:10.1021/acscatal.4c02355)
Supplement: Supplementary file 1 — cs4c02355_si_001.pdf [file cs4c02355_si_001.pdf]

Supporting Information

Hexa-Fe(III) Carboxylate Complexes Facilitate Aerobic Hydrocarbon Oxidative Functionalization: Rh Catalyzed Oxidative Coupling of Benzene and Ethylene to Form Styrene

Marc T. Bennett,† Kwanwoo A. Park,† Charles B. Musgrave III,‡ Jack W. Brubaker,‡ Diane A. Dickie,‡ William A. Goddard III‡* and T. Brent Gunnoe†*

†Department of Chemistry, University of Virginia, Charlottesville, Virginia 22904
‡Materials and Process Simulation Center, California Institute of Technology, Pasadena, CA 91125

*Correspondence to: tbg7h@virginia.edu, wag@caltech.edu

Table of Contents:

GC-MS calibration curves for vinyl pivalate, styrene, benzaldehyde, biphenyl and *trans*-stilbene……………………………………………………………………S6
Representative GC-MS chromatogram for Rh catalyzed aerobic benzene ethenylation in the presence of $Fe(OAc)_2$, HOPiv and dioxygen………………..…………………S6
Procedure for screening Fe carboxylate additives under aerobic conditions…………S6
Procedure for screening Fe carboxylate additives under anaerobic conditions………S7
Screening of Fe salts not containing carboxylate ligands in the absence of HOPiv as additives for Rh catalyzed benzene ethenlyation in the presence of 1 atm dioxygen...S8
Procedure for screening Fe additives without carboxylic acid additive under aerobic conditions………………………………………………………………………S8
Discussion of carboxylate effects for aerobic benzene ethenylation…………………S9
The effect of Fe(II) carboxylate and carboxylic acid identity on benzene ethenylation turnovers after two hours……………………………………..………………..S10
Kinetics of benzene ethenylation as a function of carboxylic acid and Fe(II) carboxylate identity……..………………………………………………………………..S13
Turnovers versus time plot for aerobic benzene ethenylation using a 1:1 mixture of $Fe(OAc)_2$ and $Fe(OPiv)_2$ in the absence of carboxylic acid……………………...S14
Discussion on elucidating the origin of limited catalysis when only OPiv ligands are used………………………………………………………………………………S14
Catalyst deactivation when using $Fe(OPiv)_2$ and HOPiv and resumption of catalysis upon addition of $[(\eta^2\text{-}C_2H_4)_2Rh(\mu\text{-}OAc)]_2$ or HOAc………………………………S15
Catalyst longevity when HOPiv is used versus a 1:1 combination of HOPiv and HOAc…………………………………………………………………………………S16
Procedure for screening the influence of acetate versus pivalate based Fe(II) carboxylates and carboxylic acids…………………………………………..…S17
Procedure for screening the influence of carboxylic acid and Fe(II) carboxylate identity on reaction kinetics……………………………………………...……..…S17
Procedure for experiments in which $[(\eta^2\text{-}C_2H_4)_2Rh(\mu\text{-}OAc)]_2$ or HOAc were added to reaction mixtures after apparent catalyst deactivation……………..……………..…S18
Discussion on studies of Fe oxidant stability under the reaction conditions………..S19
Probing the stability of the Fe based oxidant under the reaction conditions………..S20
Procedure for anaerobic benzene ethenylation kinetics using oxidized $Fe(OAc)_2$ in the presence of HOPiv…………………………………………………………..S21
Photographs of reaction mixtures upon reaction of $Fe(OAc)_2$ and HOPiv with dioxygen and after conclusion of catalysis using the oxidized Fe material as the oxidant at anaerobic conditions………..…………………………………………………S22
Procedure for aerobic benzene ethenylation kinetics using different Fe pivalate complexes…………………………………..………………………………S22
Procedure for anaerobic benzene ethenylation kinetics using different Fe pivalate complexes……………………………………….…………………………..S23
Procedure for characterization of the material generated from the reaction of HOPiv and $Fe(OPiv)_2$ with dioxygen…………………………………………………S24

Representative GC-MS chromatogram of the gas phase from the reaction of $Fe(OPiv)_2$ and HOPiv with dioxygen……………………………………………………..S24
^1H NMR spectrum of the material generated by heating $Fe(OPiv)_2$ and 2 equiv of HOPiv under 1 atm of dioxygen at 150 °C for 2 hours……………………………..S25
Representative GC-MS chromatogram for the liquid phase of the reaction of $Fe(OAc)_2$ and 2 equiv of HOPiv with dioxygen……………………………………………….S25
Representative GC-MS chromatogram for the gas phase from the reaction of $Fe^{III}_6(\mu\text{-}OH)_2(\mu_3\text{-}O)_2(\mu\text{-}OPiv)_{12}(HOPiv)_2$ and 12 equiv of HOPiv heated anaerobically at 150 °C for two hours……………………………………………………………….S26
Procedure for characterization of the material generated from the reaction of HOPiv and $Fe^{III}_6(\mu_4\text{-}O_2)(\mu_3\text{-}O)_2(\mu\text{-}OPiv)_{12}(HOPiv)_2$ under anaerobic conditions…………..S26
Representative GC-MS chromatogram for the gas phase from the reaction of $Fe^{III}_6(\mu_4\text{-}O_2)(\mu_3\text{-}O)_2(\mu\text{-}OPiv)_{12}(HOPiv)_2$ and 12 equiv of HOPiv heated anaerobically at 150 °C for two hours………………………...………………………………………S27
Procedure for ^1H NMR characterization of the material generated from the reaction of HOPiv and $Fe^{III}_6(\mu_4\text{-}O_2)(\mu_3\text{-}O)_2(\mu\text{-}OPiv)_{12}(HOPiv)_2$……………………….……S27
in situ ^1H NMR spectra of $Fe^{III}_6(\mu_4\text{-}O_2)(\mu_3\text{-}O)_2(\mu\text{-}OPiv)_{12}(HOPiv)_2$ and 12 equiv of HOPiv and its conversion to $Fe_6(\mu\text{-}OH)_2(\mu_3\text{-}O)_2(\mu\text{-}OPiv)_{12}(HOPiv)_2$ upon heating at 150 °C for two hours under anaerobic conditions……….…………………………S28
in situ ^1H NMR spectra of $Fe_6(\mu\text{-}OH)_2(\mu_3\text{-}O)_2(\mu\text{-}OPiv)_{12}(HOPiv)_2$ and 12 equiv of HOPiv before and after heating at 150 °C for 2 hours under anaerobic conditions...S28
Stacked ^1H NMR spectra of (1) material generated by heating $Fe(OPiv)_2$ and 2 equiv of HOPiv under 1 atm of dioxygen at 150 °C for 2 hours in C_6D_6 and removing the solvent, (2) material generated by heating of $Fe^{III}_6(\mu_4\text{-}O_2)(\mu_3\text{-}O)_2(\mu\text{-}OPiv)_{12}(HOPiv)_2$ and 12 equiv of HOPiv under anaerobic conditions at 150 °C in a J-Young tube in an *in situ* ^1H NMR experiment, (3) $Fe^{III}_6(\mu\text{-}OH)_2(\mu_3\text{-}O)_2(\mu\text{-}OPiv)_{12}(HOPiv)_2$ and 12 equiv of HOPiv. ………………………………………...……..………………S29
Turnovers versus time plot for aerobic benzene ethenylation reactions performed in the absence of Fe additive with only dioxygen as the oxidant…….……………………S30
Turnovers versus time plots for aerobic benzene ethenylation reactions at varying $[(\eta^2\text{-}C_2H_4)_2Rh(\mu\text{-}OAc)]_2$ concentration……………………………………………….S30
Procedure for aerobic benzene ethenylation at varying $[(\eta^2\text{-}C_2H_4)_2Rh(\mu\text{-}OAc)]_2$ concentration…….…………………………………………………………………S31
Turnovers versus time plots for aerobic benzene ethenylation reactions at varying HOPiv concentration……………………..S32
Procedure for aerobic benzene ethenylation at varying HOPiv concentration………S32
Turnovers versus time plots for anaerobic benzene ethenylation reactions at varying HOPiv concentration……………………………………………………………..S33
Procedure for anaerobic benzene ethenylation kinetics at varying HOPiv concentration………………………………………………….……………………S33
Turnovers versus time plots for aerobic benzene ethenylation reactions at varying ethylene pressure………………………...……………………………………S34
Procedure for aerobic benzene ethenylation at varying ethylene concentration……S35

Turnovers versus time plots for anaerobic benzene ethenylation reactions at varying ethylene pressure……………………………………………………………………S36
Quantification of butadiene versus alkenyl arene production using *tert*-butylbenzene as the arene substrate……………………………………………………………..S36
Procedure for studying selectivity for alkenyl arene versus butadiene production ….S36
Representative GC-MS chromatogram used to quantify the production of butadiene in *tert*-butylbenzene solvent………………………….…………………………..S37
Procedure for anaerobic benzene ethenylation kinetics at varying ethylene concentration……………………………………………………………………..S38
Turnovers versus time plots for aerobic benzene ethenylation reactions at varying $Fe(OAc)_2$ concentration………………………………………………………..S39
Procedure for aerobic benzene ethenylation at varying $Fe(OAc)_2$ concentration……S39
Turnovers versus time plots for aerobic benzene ethenylation reactions at varying dioxygen pressure………………...………………………………………………S40
Procedure for aerobic benzene ethenylation at varying dioxygen pressure…………S41
Procedure for studying aerobic benzene ethenylation catalyst longevity at varying ethylene pressure……...…………………………………………………………S41
Procedure for studying aerobic benzene ethenylation catalyst longevity at varying HOPiv concentration……………...………………………………………………...S42
Procedure for studying aerobic benzene ethenylation catalyst longevity at varying $Fe(OAc)_2$ concentration…………………………………………………………..S43
Procedure for studying aerobic benzene ethenylation catalyst longevity at varying $[(\eta^2\text{-}C_2H_4)_2Rh(\mu\text{-}OAc)]_2$ concentration…………………………………………………S43
Discussion on the effects of water, benzaldehyde or styrene additive on catalyst longevity and reaction rate…………………………………………………………..S44
Turnovers versus time at varying water, styrene or benzaldehyde concentration…..S45
Procedure for studying aerobic benzene ethenylation catalyst longevity in the presence of added styrene……………………………………………………………………..S45
Procedure for studying aerobic benzene ethenylation catalyst longevity in the presence of added benzaldehyde……………………………..…………………S46
Procedure for studying aerobic benzene ethenylation catalyst longevity in the presence of added water……………………...……………………………………………S47
Discussion of styrene oxidation to benzaldehyde……………………….………..S47
Kinetics of styrene oxidation to benzaldehyde…………………………….………..S48
Procedure for studying the kinetics of styrene oxidation to benzaldehyde…………S48
Discussion on the optimization of anaerobic catalysis…………………………...….S49
Turnovers of styrene versus time plots as a function of Fe loading……...………..S50
Turnovers of styrene versus time plots as a function of Rh loading…………………S52
Procedure for anaerobic benzene ethenylation kinetics at varying $Fe^{III}_6(\mu\text{-}OH)_2(\mu_3\text{-}O)_2(\mu\text{-}X)_{12}(HX)_2$ concentration…………………………………………………..S52
Procedure for anaerobic benzene ethenylation kinetics at varying $[(\eta^2\text{-}C_2H_4)_2Rh(\mu\text{-}OAc)]_2$ concentration……………………………………………………………..S53
Procedure for anaerobic benzene ethenylation kinetics at varying reaction temperature…………………………………………………………...………..S54

Procedure for anaerobic benzene ethenylation kinetics with varying carboxylic acid……………………………………………………………………………….S55
Turnovers versus time plot for anaerobic benzene ethenylation reactions performed with either HOPiv, HOAc or HOiBu additive……………………………………….S56
Procedure for anaerobic benzene ethenylation with a separate dioxygen-reoxidation step……………………..…………………………………………………………S56
DFT Energies for hexanuclear Fe species investigated in Figure 6 of manuscript…..S57

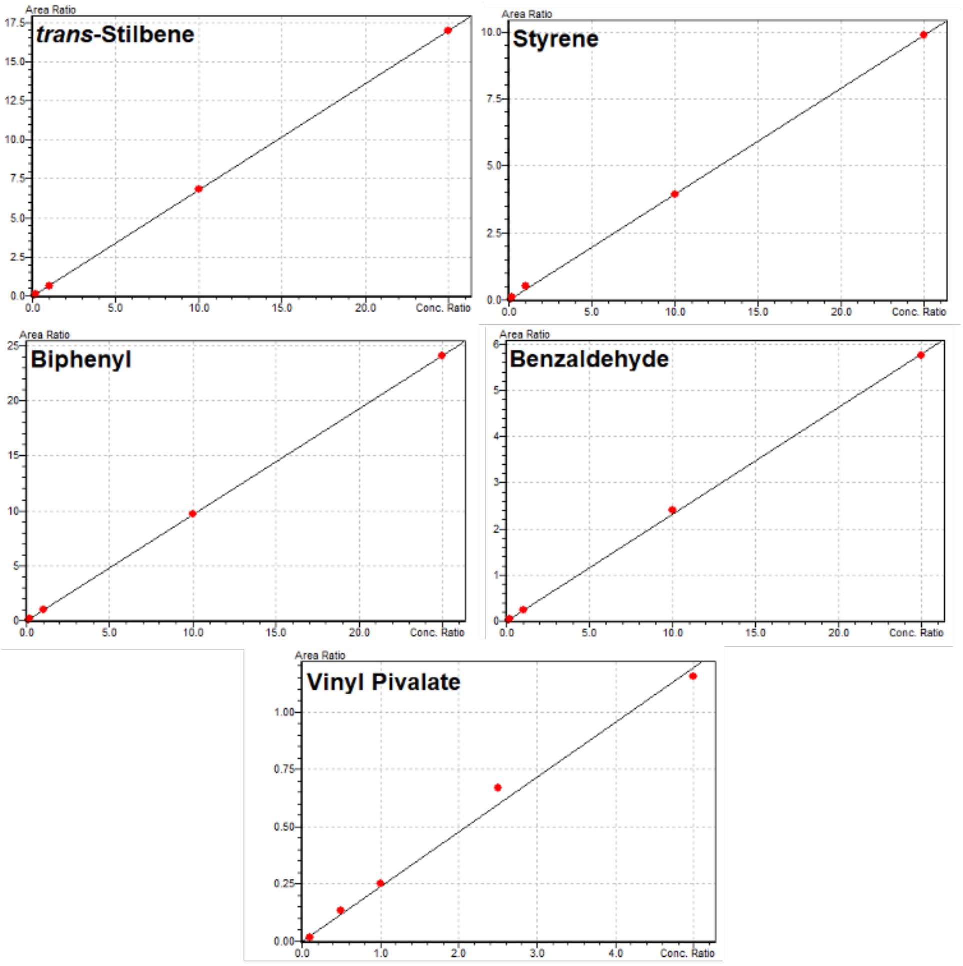

Figure S1. GC-MS calibration curves for vinyl pivalate, styrene, benzaldehyde, biphenyl and *trans*-stilbene.

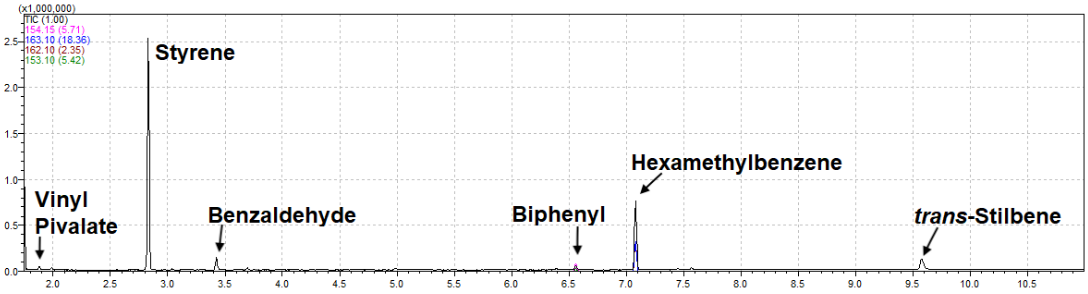

Figure S2. Representative GC-MS chromatogram for Rh catalyzed aerobic benzene ethenylation in the presence of $Fe(OAc)_2$, HOPiv and dioxygen.

Procedure for screening Fe carboxylate additives under aerobic conditions. Under an atmosphere of dry dinitrogen, three 10 mL vials with stir bars were charged with 7.5 mL (84.6 mmol) benzene, $[(\eta^2\text{-}C_2H_4)_2Rh(\mu\text{-OAc})]_2$ (0.001 mol% relative to

benzene per single Rh atom, 0.18 mg, 0.846 μmol per single Rh atom), Fe carboxylate additive (480 equiv relative to Rh per single Fe atom) and HOPiv (960 equiv relative to single Rh atom, 82.9 mg, 0.812 mmol). The vials were inserted into stainless steel reactors, which were subsequently sealed. The headspaces of the stainless-steel reactors were flushed with dioxygen by pressurizing with 15 psig of dioxygen and releasing the pressure six times, leaving 1 atm (0 psig) of dioxygen in the reactors. The reactors were subsequently pressurized with 70 psig of ethylene and heated in an aluminum block on a hot plate at 170 °C for two hours. Upon cooling to room temperature, reactors were sampled in air using a long needle. Next, 50 μL aliquots of the reaction mixtures were combined with 50 μL of a 11.1 mM hexamethylbenzene benzene solution and diluted in 0.25 mL of benzene to give 100 equiv of external standard hexamethylbenzene. The benzene solutions were washed with a saturated aqueous solution of NaOH (1.5 mL) to remove Fe complexes and HOPiv, and the organic layer was analyzed by GC-MS.

Procedure for screening Fe carboxylate additives under anaerobic conditions. Under an atmosphere of dry dinitrogen, three 10 mL vials with stir bars were charged with 7.5 mL (84.6 mmol) benzene, $[(\eta^2\text{-}C_2H_4)_2Rh(\mu\text{-}OAc)]_2$ (0.001 mol% relative to benzene per single Rh atom, 0.18 mg, 0.846 μmol per single Rh atom), Fe carboxylate additive (480 equiv relative to Rh per single Fe atom) and HOPiv (960 equiv relative to single Rh atom, 82.9 mg, 0.812 mmol). The vials were inserted into stainless steel reactors. The reactors were sealed subsequently pressurized with 70 psig of ethylene and heated in an aluminum block on a hot plate at 170 °C for two hours. Upon cooling to room temperature, reactors were sampled in air using a long needle. Next, 50 μL

aliquots of the reaction mixtures were combined with 50 μL of a 11.1 mM hexamethylbenzene benzene solution and diluted in 0.25 mL of benzene to give 100 equiv of external standard hexamethylbenzene. The benzene solutions were washed with a saturated aqueous solution of NaOH (1.5 mL) to remove Fe complexes and HOPiv, and the organic layer was analyzed by GC-MS.

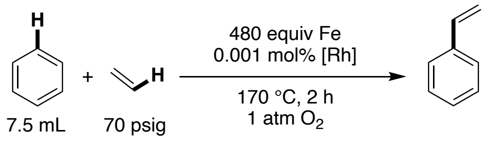

Additive	TOs Styrene
$FeCl_2$	trace
$FeCl_3$	trace
$Fe(acac)_3$	trace
Fe_2O_3	trace
Ferrocene	trace

Table S1. Screening of Fe salts not containing carboxylate ligands and in the absence of HOPiv as additives for Rh catalyzed benzene ethenlyation in the presence of 1 atm dioxygen. Reaction conditions: 7.5 mL benzene, 0.001 mol% (relative to benzene per single Rh atom) $[(\eta^2\text{-}C_2H_4)_2Rh(\mu\text{-}OAc)]$, 480 equiv (relative to Rh per single Fe atom) Fe additive, 70 psig ethylene, 1 atm dioxygen, 170 °C, 2 hours. The results are the average from a minimum of three independent reactions.

Procedure for screening Fe additives without carboxylic acid additive under aerobic conditions. Under an atmosphere of dry dinitrogen, three 10 mL vials with stir bars were charged with 7.5 mL (84.6 mmol) benzene, $[(\eta^2\text{-}C_2H_4)_2Rh(\mu\text{-}OAc)]_2$ (0.001 mol% relative to benzene per single Rh atom, 0.18 mg, 0.846 μmol per single Rh atom) and Fe additive (480 equiv relative to Rh per single Fe atom). The vials were inserted into stainless steel reactors, which were subsequently sealed. The headspaces of the stainless-steel reactors were flushed with dioxygen by pressurizing with 15 psig of

dioxygen and releasing the pressure six times, leaving 1 atm (0 psig) of dioxygen in the reactors. The reactors were subsequently pressurized with 70 psig of ethylene and heated in an aluminum block on a hot plate at 170 °C for two hours. Upon cooling to room temperature, reactors were sampled in air using a long needle. Next, 50 μL aliquots of the reaction mixtures were combined with 50 μL of a 11.1 mM hexamethylbenzene benzene solution and diluted in 0.25 mL of benzene to give 100 equiv of external standard hexamethylbenzene. The benzene solutions were washed with a saturated aqueous solution of NaOH (1.5 mL) to remove Fe complexes and carboxylic acid, and the organic layer was analyzed by GC-MS.

Discussion of Carboxylate Effects. Having identified that the combination of $Fe(OAc)_2$ and dioxygen gives the most substantial improvement in catalyst activity relative to the reaction with dioxygen alone, use of $Fe(OPiv)_2$ in place of $Fe(OAc)_2$ was studied (Figure S3). Previously, our group found that use of $Cu(OAc)_2$ as the oxidant, which is minimally soluble in benzene, resulted in reduced reaction rates relative to benzene-soluble Cu(II) carboxylates such as $Cu(OPiv)_2$ or $Cu(OHex)_2$ (OHex = 2-ethylhexanoate).[62, 63, 79] Benzene ethenylation reactions at 170 °C under one atm of dioxygen were probed with 7.5 mL benzene, 70 psig ethylene, 0.001 mol% $[(\eta^2\text{-}C_2H_4)_2Rh(\mu\text{-}OAc)]_2$ (relative to benzene per single Rh atom), 0.480 mol% of Fe carboxylate additive and either 0 or 0.960 mol% of either HOAc or HOPiv. In the absence of added carboxylic acid, use of $Fe(OPiv)_2$ additive resulted in 104(16) TOs of styrene after two hours, and the use of $Fe(OAc)_2$ additive resulted in 249(24) TOs of styrene. The combination of $Fe(OPiv)_2$ and HOPiv additives results in 111(3) TOs of

styrene after two hours, and the combination of $Fe(OAc)_2$ and HOAc results in 146(29) TOs. The apparent improvement in catalysis when $Fe(OAc)_2$ (with and without HOAc) is used in place of $Fe(OPiv)_2$ (with and without HOPiv) is somewhat surprising since the generated material is only partially soluble in benzene. As discussed above, the combination of HOPiv and $Fe(OAc)_2$ produces a soluble material, which forms 371(28) TOs of styrene after two hours. A statistically identical number of TOs {393(25)} were observed after two hours with the combination of $Fe(OPiv)_2$ and HOAc, suggesting that a 1:1 combination of OAc and OPiv ligands either enhances the reaction rate or the longevity of the catalysis. These findings suggest that (1) solubility of the active oxidant, which is imparted by OPiv groups, is important to catalyst activity, and (2) reaction conditions in which only OPiv groups are present result in poorer catalyst activity and/or longevity relative to reaction conditions that combine OAc and OPiv ligands. These observations are discussed in more detail below.

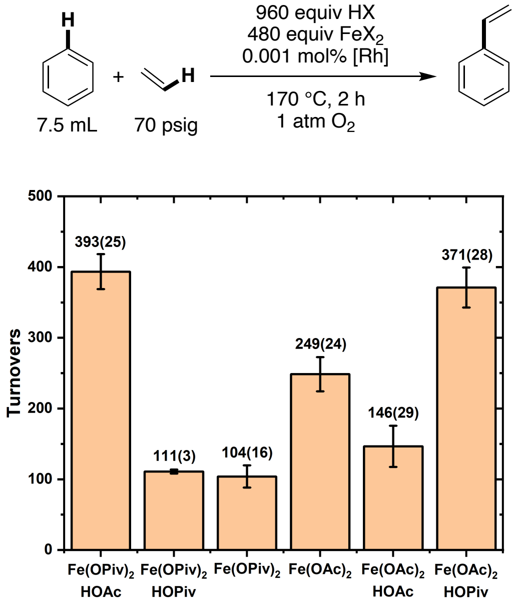

Figure S3. The effect of Fe(II) carboxylate identity and carboxylic acid identity on benzene ethenylation TOs after two hours. Reaction conditions: 7.5 mL benzene, 0.001 mol% (relative to benzene per single Rh atom) $[(\eta^2\text{-}C_2H_4)_2Rh(\mu\text{-}OAc)]_2$, 480 equiv

(relative to Rh) $Fe(OAc)_2$ or $Fe(OPiv)_2$, 960 equiv HOPiv, 70 psig ethylene, 1 atm dioxygen, 170 °C, 2 hours. All data points reflect the average of a minimum of three independent reactions and error bars represent the standard deviation from the multiple independent experiments.

Having observed that carboxylate identity has a significant effect on catalyst activity and/or longevity, we speculated that the catalysis could be further optimized by use of other $Fe(OAc)_2$/carboxylic acid combinations. Accordingly, the kinetics of benzene ethenylation at 150 °C were probed with 7.5 mL of benzene, 70 psig of ethylene, 0.001 mol% $[(\eta^2\text{-}C_2H_4)_2Rh(\mu\text{-}OAc)]_2$ (relative to benzene per single Rh atom), 480 equiv of $Fe(OAc)_2$ (relative to Rh) and 960 equiv of benzoic acid (HOBz), acetic acid (HOAc), propionic acid (HOPr), isobutyric acid (HoiBu), 2-ethylhexanoic acid (HOHex), pivalic acid (HOPiv), or 1-adamatane carboxylic acid (HOAd). It is important to note that with the exception of HOBz, the pKa trend of the carboxylic acids probed follows the steric trend (HOAc < HOPr < HOiBu < HOPiv < HOAd), so it is possible that effects from both acid pKa and sterics are playing a role in the observed trends. As shown in Figure S4a, use of HOBz additive with $Fe(OAc)_2$ gives a similar rate to the reaction performed in the absence of carboxylic acid, likely because it does not impart solubility of the formed oxidized Fe carboxylate. Use of HOAc additive gives a slightly slower rate than the reaction in the absence of added carboxylic acid. HOPr gives slightly faster catalysis than HOBz, and the fastest catalysis is observed with HOiBu and HOPiv. Use of HOHex and HOAd provides a similar initial rate to catalysis with HOiBu and HOPiv, however a deceleration in rate is observed after two hours. These results indicate that bulkier carboxylic acids, which impart

greater solubility of the Fe carboxylate, result in improved catalysis when $Fe(OAc)_2$ is used, and perhaps indicate that HOHex and HOAd, which bear large alkyl substituents promote a deactivation pathway.

To complement the studies with $Fe(OAc)_2$, the effect of using different carboxylic acids in combination with $Fe(OPiv)_2$ was probed. Use of HOBz with $Fe(OPiv)_2$ gives similar results to use of HOBz with $Fe(OAc)_2$, likely as the result of HOBz imparting only partial solubility of the formed oxidized Fe species. As shown in Figure S4b, use of HOPiv and HOAd in combination with $Fe(OPiv)_2$ gives slow, short-lived catalysis, similar to the reaction performed in the absence of carboxylic acid. A slight improvement over HOAd and HOPiv is observed with HOHex additive, and HoiBu gives further improvement. The fastest initial TOF is observed with HOPiv and HOPr. Interestingly, with each carboxylic acid, non-linearity in the TOs versus time plots is observed, perhaps suggesting that a deactivation pathway more readily occurs with $Fe(OPiv)_2$ starting material versus $Fe(OAc)_2$ starting material. The striking observation that less sterically bulky carboxylic acids give improved catalysis when using $Fe(OPiv)_2$, whereas bulkier carboxylic acids give improved catalysis when using $Fe(OAc)_2$, suggests that the catalysis is optimized when there is a combination of sterically-bulky and less sterically-bulky carboxylate groups present. As discussed above, the bulkier carboxylate groups promote solubility of the formed oxidized Fe carboxylate, which is likely the origin of the carboxylic acid trend when using $Fe(OAc)_2$. In contrast, when using $Fe(OPiv)_2$, bulky carboxylic acids result in a slower initial rate and short-lived catalysis. Importantly, the presence of carboxylic acid

additive is necessary, as use of a 1:1 ratio of $Fe(OAc)_2$:$Fe(OPiv)_2$ did not result in improved catalysis relative to use of either complex alone (Figure S5). From these results, the combination of $Fe(OAc)_2$ and HOPiv was identified as optimal, as no evidence of catalyst deactivation was observed after six hours.

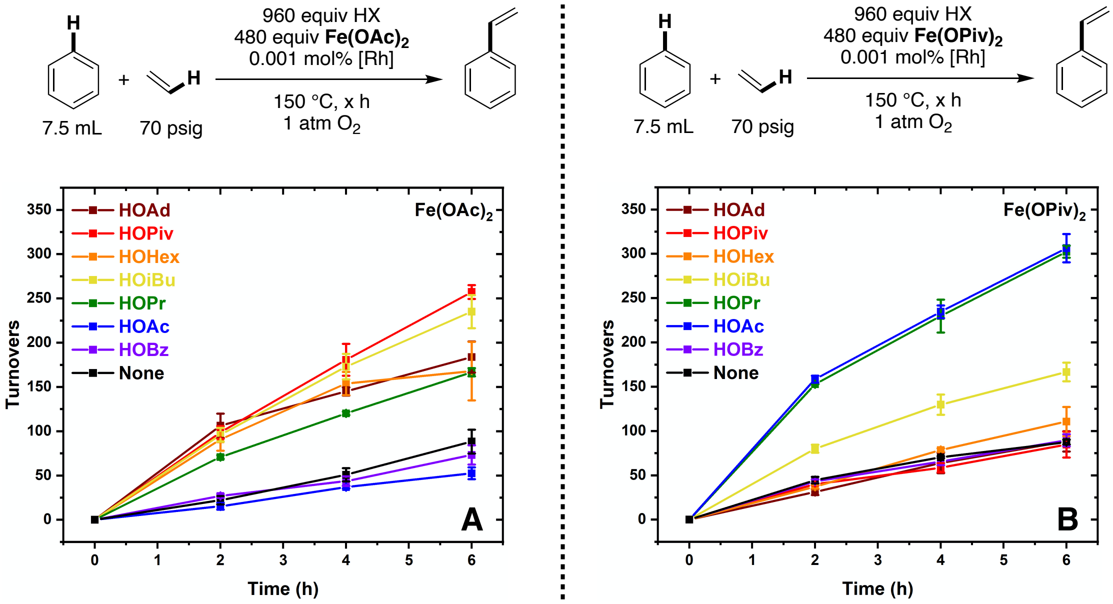

Figure S4. Kinetics of benzene ethenylation as a function of carboxylic acid and Fe(II) carboxylate identity. Reaction conditions: 7.5 mL benzene, 0.001 mol% (relative to benzene per single Rh atom) $[(\eta^2\text{-}C_2H_4)_2Rh(\mu\text{-}OAc)]_2$, 480 equiv (relative to Rh) $Fe(OAc)_2$ or $Fe(OPiv)_2$, 960 equiv HX, 70 psig ethylene, 1 atm dioxygen, 150 °C, x hours. All data points reflect the average of a minimum of three independent reactions and error bars represent the standard deviation from the three trials.

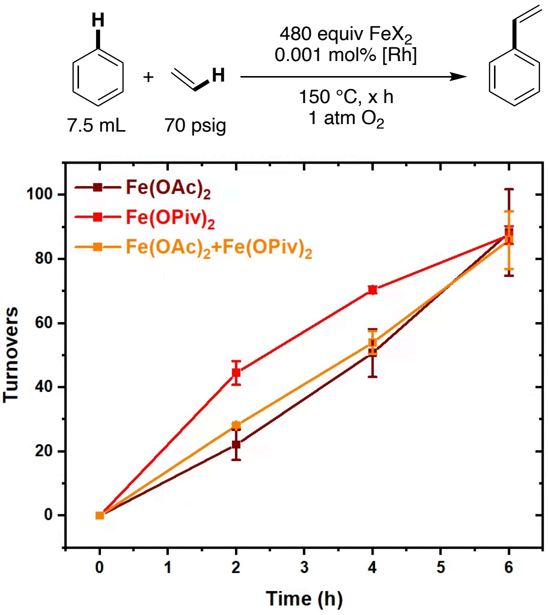

Figure S5. Turnovers versus time plot for aerobic benzene ethenylation performed in the absence of carboxylic acid additive with $Fe(OAc)_2$, $Fe(OPiv)_2$ or a 1:1 molar ratio of $Fe(OAc)_2$ and $Fe(OPiv)_2$. Reaction conditions: 7.5 mL benzene, 0.001 mol% (relative to benzene per single Rh atom) $[(\eta^2\text{-}C_2H_4)_2Rh(\mu\text{-}OAc)]_2$, 480 equiv (relative to Rh) $Fe(OAc)_2$, $Fe(OPiv)_2$, or 240 equiv of both $Fe(OAc)_2$ and $Fe(OPiv)_2$ 960 equiv HX, 70 psig ethylene, 1 atm dioxygen, 150 °C, x hours. All data points reflect the average of a minimum of three independent reactions and error bars represent the standard deviation from the three trials.

Elucidating the origin of limited catalysis when only OPiv ligands are used. As discussed above, limited catalysis is observed under aerobic conditions when $Fe(OPiv)_2$ and HOPiv are used, whereas faster and longer-lived catalysis is observed when a combination of OAc and OPiv sources are present. To determine whether the observed deactivation can be attributed to Rh deactivation, additional $[(\eta^2\text{-}C_2H_4)_2Rh(\mu\text{-}OAc)]_2$ was added to a deactivated reaction mixture in which $Fe(OPiv)_2$ and HOPiv were used to determine if catalysis continues (Figure S6). In Figure S6, the reported TOs are relative to the initial loading of $[(\eta^2\text{-}C_2H_4)_2Rh(\mu\text{-}OAc)]_2$. The results indicate that catalysis continues after addition of $[(\eta^2\text{-}C_2H_4)_2Rh(\mu\text{-}OAc)]_2$, suggesting that Rh deactivation is likely the origin of limited catalysis when only OPiv functional groups

are present. To probe whether the observed Rh deactivation can be reversed by addition of HOAc, 960 equiv of HOAc were added to a deactivated reaction mixture. As shown in Figure S6b, addition of HOAc results in a resumption of catalysis, indicating that Rh deactivation can be reversed by HOAc. These findings are consistent with reversible formation of an inactive (off-cycle) intermediate that is favored when OPiv functionalities are present but disfavored in the presence of OAc.

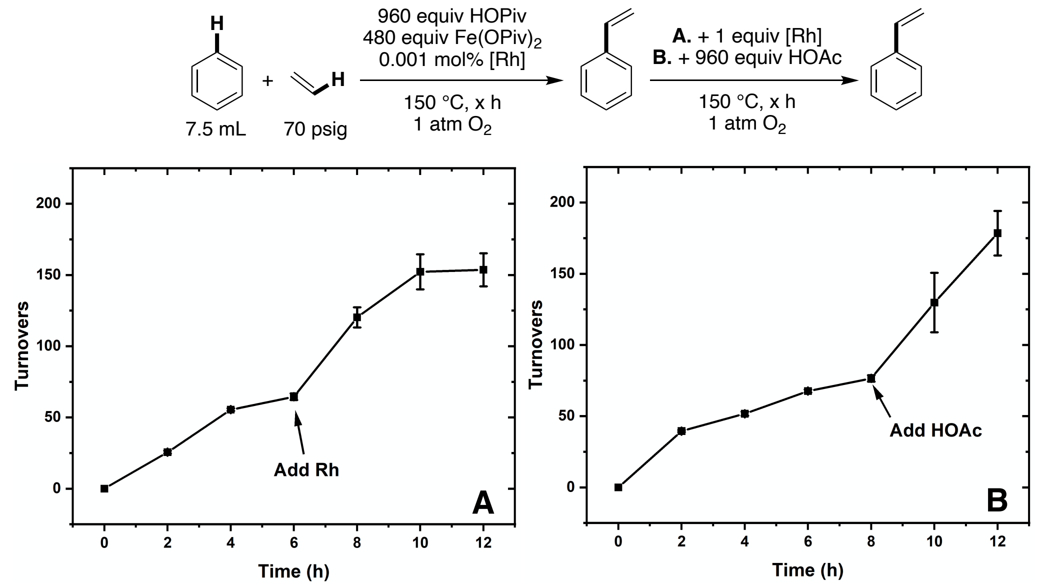

Figure S6. Catalyst deactivation observed when using $Fe(OPiv)_2$ in the presence of HOPiv, and resumption of catalysis upon addition of $[(\eta^2\text{-}C_2H_4)_2Rh(\mu\text{-}OAc)]_2$ or HOAc. Reaction conditions: 7.5 mL benzene, 0.001 mol% (relative to benzene per single Rh atom) $[(\eta^2\text{-}C_2H_4)_2Rh(\mu\text{-}OAc)]_2$, 480 equiv (relative to Rh) $Fe(OPiv)_2$, 960 equiv HOPiv, 70 psig ethylene, 1 atm dioxygen, 150 °C. The reactions were carried out until catalyst deactivation was observed, and either $[(\eta^2\text{-}C_2H_4)_2Rh(\mu\text{-}OAc)]_2$ (1 equiv relative to original Rh loading) or HOAc (960 equiv relative to original Rh loading) were added. Reported TOs are relative to the original Rh loading. All data points reflect the average of a minimum of three independent reactions and error bars represent the standard deviation from the multiple independent experiments.

As is discussed above, the initial reaction rate and catalyst longevity are inferior when using $Fe(OPiv)_2$ and HOPiv versus conditions with a 1:1 OPiv:OAc ratio, which

is likely the result of OPiv ligands promoting Rh deactivation. To probe whether the observed inverse dependence on HOPiv concentration is the result of increasing the OPiv:OAc ratio, carboxylic acid concentration dependence studies were carried out with a 1:1 ratio of HOPiv:HOAc (Figure S7). The results indicate statistically identical initial reaction rates at each carboxylic acid concentration when HOPiv is used versus a 1:1 ratio of HOPiv:HOAc at the same total concentration. Also, when the carboxylic acid concentration is increased to 7680 total equiv of carboxylic acid, the reaction with HOPiv undergoes apparent deactivation, while the reaction with a 1:1 combination of HOAc and HOPiv maintains the initial reaction rate. These observations are consistent with HOAc and HOPiv having similar kinetic inhibitory effects on the reaction rate and with higher OPiv:OAc ratios promoting Rh catalyst deactivation at extended reaction time.

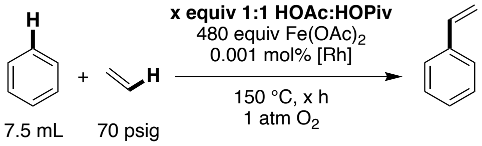

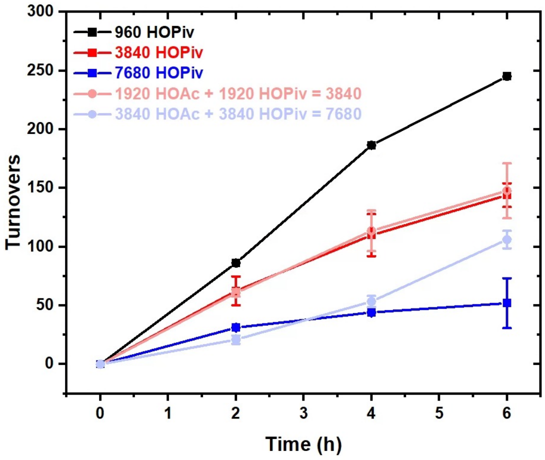

Figure S7. TOs versus time plots for catalysis using either HOPiv or a 1:1 ratio of HOPiv and HOAc as a function of carboxylic acid concentration. Reaction conditions: 7.5 mL benzene, 0.001 mol% (relative to benzene per single Rh atom) $[(\eta^2\text{-}C_2H_4)_2Rh(\mu\text{-}OAc)]$, 480 equiv $Fe(OAc)_2$, x equiv HOPiv or 1:1 ratio of HOAc and HOPiv, 70 psig

ethylene, 1 atm dioxygen, 150 °C. All data points reflect the average of a minimum of three independent reactions and error bars represent the standard deviation from the multiple independent experiments.

Procedure for screening the influence of acetate versus pivalate based Fe(II) carboxylates and carboxylic acids. Under an atmosphere of dry dinitrogen, three 10 mL vials with stir bars were charged with 7.5 mL (84.6 mmol) benzene, $[(\eta^2\text{-}C_2H_4)_2Rh(\mu\text{-}OAc)]_2$ (0.001 mol% relative to benzene per single Rh atom, 0.18 mg, 0.846 μmol), HOAc (960 equiv relative to Rh, 47 μL, 0.812 mmol) or HOPiv (960 equiv relative to Rh, 82.9 mg, 0.812 mmol) or no carboxylic acid, and $Fe(OAc)_2$ (480 equiv relative to Rh, 69.9 mg, 0.406 mmol) or $Fe(OPiv)_2$ (480 equiv relative to Rh, 107.6 mg, 0.406 mmol). The vials were inserted into stainless steel reactors, which were subsequently sealed. The headspaces of the stainless-steel reactors were flushed with dioxygen by pressurizing with 15 psig of dioxygen and releasing the pressure six times, leaving 1 atm (0 psig) of dioxygen in the reactors. The reactors were subsequently pressurized with 70 psig of ethylene and heated in an aluminum block on a hot plate at 170 °C for two hours. Upon cooling to room temperature, reactors were sampled in air using a long needle. Next, 50 μL aliquots of the reaction mixtures were combined with 50 μL of a 11.1 mM hexamethylbenzene benzene solution and diluted in 0.25 mL of benzene to give 100 equiv of external standard hexamethylbenzene. The benzene solutions were washed with a saturated aqueous solution of NaOH (1.5 mL) to remove Fe complexes and carboxylic acid, and the organic layer was analyzed by GC-MS.

Procedure for screening the influence of carboxylic acid and Fe(II) carboxylate identity on reaction kinetics. Under an atmosphere of dry dinitrogen, three 10 mL

vials with stir bars were charged with 7.5 mL (84.6 mmol) benzene, $[(\eta^2\text{-}C_2H_4)_2Rh(\mu\text{-}OAc)]_2$ (0.001 mol% relative to benzene per single Rh atom, 0.18 mg, 0.846 μmol), benzoic (960 equiv relative to Rh, 99.1 mg, 0.812 mmol), acetic (960 equiv relative to Rh, 47 μL, 0.812 mmol), propionic (960 equiv relative to Rh, 61 μL, 0.812 mmol), isobutyric acid (960 equiv relative to Rh, 74 μL, 0.812 mmol), 2-ethyl hexanoic acid (960 equiv relative to Rh, 130 μL, 0.812 mmol), pivalic acid (960 equiv relative to Rh, 82.9 mg, 0.812 mmol), 1-adamantanecarboxylic acid (960 equiv relative to Rh, 146.2 mg, 0.812 mmol, or no carboxylic acid, and either $Fe(OAc)_2$ (480 equiv relative to Rh, 69.9 mg, 0.406 mmol) or $Fe(OPiv)_2$ (480 equiv relative to Rh, 107.6 mg, 0.406 mmol). The vials were inserted into stainless steel reactors, which were subsequently sealed. The headspaces of the stainless-steel reactors were flushed with dioxygen by pressurizing with 15 psig of dioxygen and releasing the pressure six times, leaving 1 atm (0 psig) of dioxygen in the reactors. The reactors were subsequently pressurized with 70 psig of ethylene and heated in an aluminum block on a hot plate at 150 °C for two-hour intervals. Upon cooling to room temperature, reactors were sampled in air using a long needle. Next, 50 μL aliquots of the reaction mixtures were combined with 50 μL of a 11.1 mM hexamethylbenzene benzene solution and diluted in 0.25 mL of benzene to give 100 equiv of external standard hexamethylbenzene. The benzene solutions were washed with a saturated aqueous solution of NaOH (1.5 mL) to remove Fe complexes and carboxylic acid, and the organic layer was analyzed by GC-MS.

Procedure for experiments in which $[(\eta^2\text{-}C_2H_4)_2Rh(\mu\text{-}OAc)]_2$ or HOAc were added to reaction mixtures after apparent catalyst deactivation. Under an

atmosphere of dry dinitrogen, three 10 mL vials with stir bars were charged with 7.5 mL (84.6 mmol) benzene, $[(\eta^2\text{-}C_2H_4)_2Rh(\mu\text{-Oac})]_2$ (0.001 mol% relative to benzene per single Rh atom, 0.18 mg, 0.846 μmol), HOPiv (960 equiv relative to Rh, 82.9 mg, 0.812 mmol) and $Fe(Opiv)_2$ (480 equiv relative to Rh, 107.6 mg, 0.406 mmol). The vials were inserted into stainless steel reactors, which were subsequently sealed. The headspaces of the stainless-steel reactors were flushed with dioxygen by pressurizing with 15 psig of dioxygen and releasing the pressure six times, leaving 1 atm (0 psig) of dioxygen in the reactors. The reactors were subsequently pressurized with 70 psig of ethylene and heated in an aluminum block on a hot plate at 150 °C for two-hour intervals. Upon cooling to room temperature, reactors were sampled in air using a long needle. Next, 50 μL aliquots of the reaction mixtures were combined with 50 μL of a 11.1 mM hexamethylbenzene benzene solution and diluted in 0.25 mL of benzene to give 100 equiv of external standard hexamethylbenzene. The benzene solutions were washed with a saturated aqueous solution of NaOH (1.5 mL) to remove Fe complexes and carboxylic acid, and the organic layer was analyzed by GC-MS. After the initial reaction rate was found to substantially decrease, HOAc (960 equiv relative to Rh, 46 μL, 0.812 mmol) or $[(\eta^2\text{-}C_2H_4)_2Rh(\mu\text{-OAc})]_2$ (0.001 mol% relative to Rh, 0.18 mg, 0.846 μmol) dissolved in 2.5 mL of benzene were added to each reactor in air, and the reactions were continued.

Studies of Fe oxidant stability under the reaction conditions. To determine if the active oxidant is thermally unstable or reactive towards ethylene, we probed whether heating the active oxidant under the reaction conditions prior to the addition of

Rh results in decreased activity (Figure S8). In two experiments, the active oxidant was generated and subsequently heated at 170 °C under anaerobic conditions either in the presence or absence of 70 psig of ethylene. Next, $[(\eta^2\text{-}C_2H_4)_2Rh(\mu\text{-}OAc)]_2$ and ethylene were added to the Fe material, and the reactions were heated for two hours. Statistically identical TOs were observed for the experiments in which the oxidized material was heated in the presence and absence of ethylene relative to a control reaction in which the oxidized material was not heated prior to catalysis. These results indicate that the active Fe oxidant is likely stable under the reaction conditions and is minimally reactive towards benzene and ethylene. Thus, the observation of ~40 TOs when 480 equiv of Fe are present is not likely the result of oxidant instability.

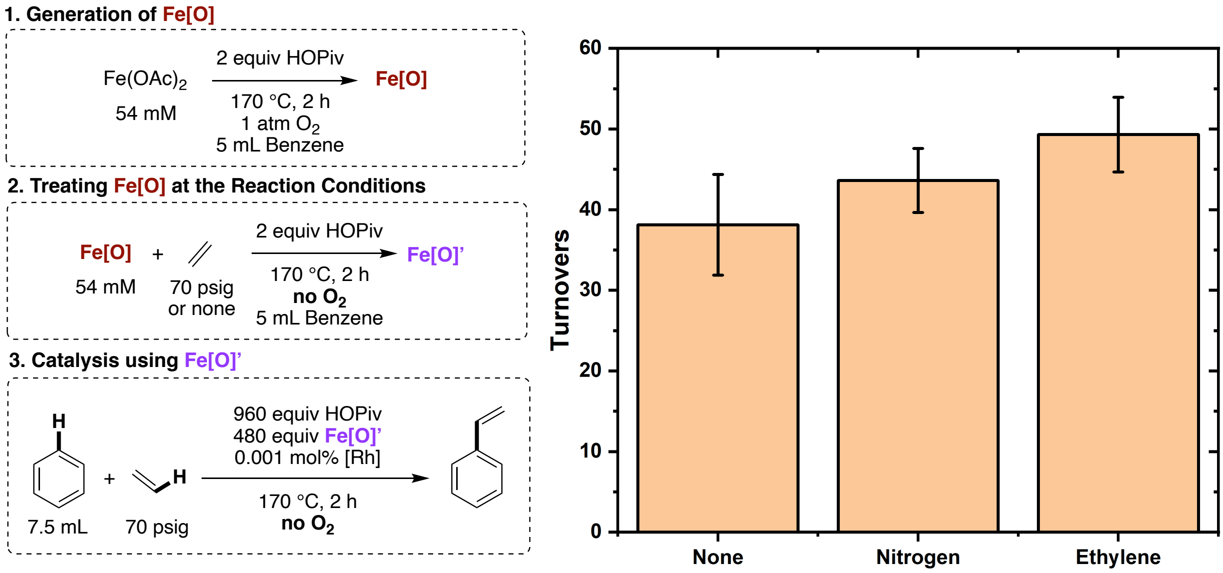

Figure S8. TOs of styrene after two hours of benzene ethenylation reactions using the material generated by reaction of $Fe(OAc)_2$ and HOPiv with dioxygen under anaerobic conditions, which was heated either in the presence or absence of ethylene prior to catalysis. Reaction conditions: Reaction 1: 5 mL benzene, 0.0042 moles of $Fe(OAc)_2$, 0.0084 moles of HOPiv, 1 atm dioxygen and 70 psig N_2, 170 °C, 2 hours to form Fe[O]. Reaction 2: dioxygen was removed from the reaction mixtures from Reaction 1, and reactors were pressurized with 70 psig of either dinitrogen or ethylene to form Fe[O]'. Note: for the reaction labeled "None" in the bar graph, the material from reaction 1 was used without performing reaction 2. Reaction 3: 5 mL solutions from Reaction 1 were combined with $[(\eta^2\text{-}C_2H_4)_2Rh(\mu\text{-}OAc)]_2$ in 2.5 mL of benzene to give 7.5 mL benzene

along with 0.001 mol% [(η^2-C_2H_4)$_2$Rh(μ-OAc)]$_2$, 480 equiv Fe[O]' (relative to Rh per single Fe atom), 960 equiv HOPiv, 70 psig ethylene, 170 °C. All data points reflect the average of a minimum of three independent reactions and error bars represent the standard deviation from the multiple independent experiments.

Procedure for anaerobic benzene ethenylation kinetics using oxidized $Fe(OAc)_2$ in the presence of HOPiv. Under an atmosphere of dry dinitrogen, three 10 mL vials with stir bars were charged with 5 mL (84.6 mmol) benzene, $Fe(OAc)_2$ (69.9 mg, 0.406 mmol), HOPiv (82.9 mg, 0.812 mmol). The vials were inserted into previously described stainless steel reactors. The reactors were subsequently pressurized with 70 psig of dinitrogen and 15 psig of dioxygen and heated in an aluminum block on a hot plate at 170 °C for two hours. The quantity of [(η^2-C_2H_4)$_2$Rh(μ-OAc)]$_2$ for three reactions (0.553 mg, 2.54 μmol) was added to 7.5 mL of benzene, and 2.5 mL aliquots of this solution were added to each of the three stainless steel reactors. Dioxygen was removed from the reactors by cycling between dinitrogen pressure and partial vacuum on a high-pressure line, and the reactors were subsequently pressurized with 70 psig of ethylene. The reactors were heated in an aluminum block on a hot plate for 20-minute intervals for the first hour, and again after two and four-hour reaction times. Next, 50 μL aliquots of the reaction mixtures were combined with 50 μL of an 11.1 mM benzene solution of hexamethylbenzene in 0.25 mL of benzene to give 200 equiv of external standard hexamethylbenzene. The benzene solutions were washed with a saturated aqueous solution of NaOH (1.5 mL) to remove Fe salts and carboxylic acid, and the organic layer was analyzed by GC-MS.

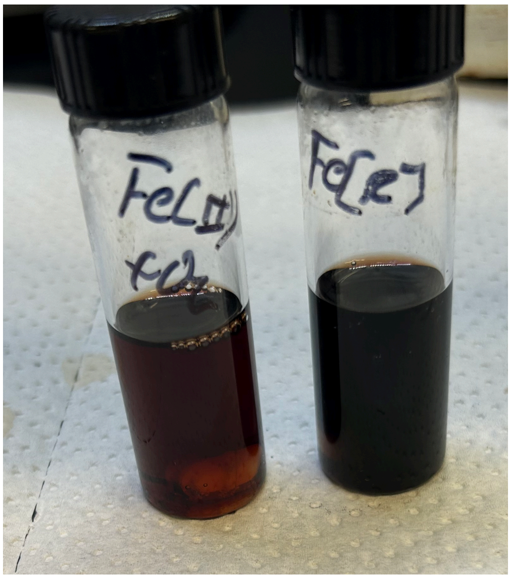

Figure S9. Photographs of reaction mixtures upon reaction of $Fe(OAc)_2$ and HOPiv with dioxygen (left) and after conclusion of catalysis using the oxidized Fe material as the oxidant at anaerobic conditions (right).

Procedure for aerobic benzene ethenylation kinetics using different Fe pivalate complexes. Under an atmosphere of dry dinitrogen, three 10 mL vials with stir bars were charged with 7.5 mL (84.6 mmol) benzene, $[(\eta^2\text{-}C_2H_4)_2Rh(\mu\text{-}OAc)]_2$ (0.001 mol% relative to benzene per single Rh atom, 0.18 mg, 0.846 μmol), HOAc (960 equiv relative to Rh, 47 μL, 0.812 mmol) and either $[Fe^{III}_3(\mu_3\text{-}O)(\mu\text{-}OPiv)_6(H_2O)_3][OPiv]$ (160 equiv, 127.9 mg, 0.135 mmol), $Fe^{III}_2Fe^{II}(\mu_3\text{-}O)(\mu\text{-}OPiv)_6(HOPiv)_3$ (160 equiv, 148.5 mg 0.135 mmol), $Fe^{III}_6(\mu_4\text{-}O_2)(\mu_3\text{-}O)_2(\mu\text{-}OPiv)_{12}(HOPiv)_2$ (80 equiv, 122.9 mg, 0.0679 mmol), or $Fe^{III}_6(\mu\text{-}OH)_2(\mu_3\text{-}O)_2(\mu\text{-}OPiv)_{12}(HOPiv)_2$ (80 equiv, 123.0 mg, 0.0679 mmol). The vials were inserted into stainless steel reactors, which were subsequently sealed. The headspaces of the stainless-steel reactors were flushed with dioxygen by pressurizing with 15 psig of dioxygen and releasing the pressure six times, leaving 1 atm (0 psig) of dioxygen in the reactors. The reactors were subsequently pressurized with 70 psig of ethylene and heated in an aluminum block on a hot plate at

150 °C for two-hour intervals. Upon cooling to room temperature, reactors were sampled in air using a long needle. Next, 50 μL aliquots of the reaction mixtures were combined with 50 μL of a 11.1 mM hexamethylbenzene benzene solution and diluted in 0.25 mL of benzene to give 100 equiv of external standard hexamethylbenzene. The benzene solutions were washed with a saturated aqueous solution of NaOH (1.5 mL) to remove Fe complexes and carboxylic acid, and the organic layer was analyzed by GC-MS.

Procedure for anaerobic benzene ethenylation kinetics using different Fe pivalate complexes. Under an atmosphere of dry dinitrogen, three 10 mL vials with stir bars were charged with 7.5 mL (84.6 mmol) benzene, $[(\eta^2\text{-}C_2H_4)_2Rh(\mu\text{-}OAc)]_2$ (0.001 mol% relative to benzene per single Rh atom, 0.18 mg, 0.846 μmol), HOAc (960 equiv relative to Rh, 47 μL, 0.812 mmol) and either $Fe^{III}{}_2Fe^{II}(\mu_3\text{-}O)(\mu\text{-}OPiv)_6(HOPiv)_3$ (160 equiv, 148.5 mg 0.135 mmol), $Fe^{III}{}_6(\mu_4\text{-}O_2)(\mu_3\text{-}O)_2(\mu\text{-}OPiv)_{12}(HOPiv)_2$ (80 equiv, 122.9 mg, 0.0679 mmol), or $Fe^{III}{}_6(\mu\text{-}OH)_2(\mu_3\text{-}O)_2(\mu\text{-}OPiv)_{12}(HOPiv)_2$ (80 equiv, 123.0 mg, 0.0679 mmol). The vials were inserted into stainless steel reactors, which were subsequently sealed. The reactors were subsequently pressurized with 70 psig of ethylene and heated in an aluminum block on a hot plate at 150 °C for two-hour intervals. Upon cooling to room temperature, reactors were sampled in air using a long needle. Next, 50 μL aliquots of the reaction mixtures were combined with 50 μL of a 11.1 mM hexamethylbenzene benzene solution and diluted in 0.25 mL of benzene to give 100 equiv of external standard hexamethylbenzene. The benzene solutions were

washed with a saturated aqueous solution of NaOH (1.5 mL) to remove Fe complexes and carboxylic acid, and the organic layer was analyzed by GC-MS.

Procedure for characterization of the material generated from the reaction of HOPiv and Fe(OPiv)$_2$ with dioxygen. Under an atmosphere of dry dinitrogen, 10 mL vials equipped with stir bars were charged with 7.5 mL (84.6 mmol) benzene, HOPiv (0.960 mol% relative to benzene, 82.9 mg, 0.812 mmol) and Fe(OPiv)$_2$ (0.480 mol% relative to benzene, 107.6 mg, 0.406 mmol). The vials were inserted into stainless steel reactors, which were sealed and subsequently flushed with dioxygen by pressurizing the reactors with 15 psig of dioxygen and releasing the pressure six times, leaving one atm (0 psig). The reactors were then pressurized with 70 psig of dinitrogen and heated on an aluminum heating block on a hot plate at 150 °C for two hours. After cooling to room temperature, the gas phase was collected in Tedlar bags for GC-MS analysis. The reaction solutions were either (1) transferred to test tubes for slow evaporation crystallization, (2) 0.1 mL aliquots were diluted in benzene to a total volume of 5 mL, and UV-Visible spectroscopy was performed or (3) benzene was removed *in vacuo* for ^{1}H NMR spectroscopy analysis.

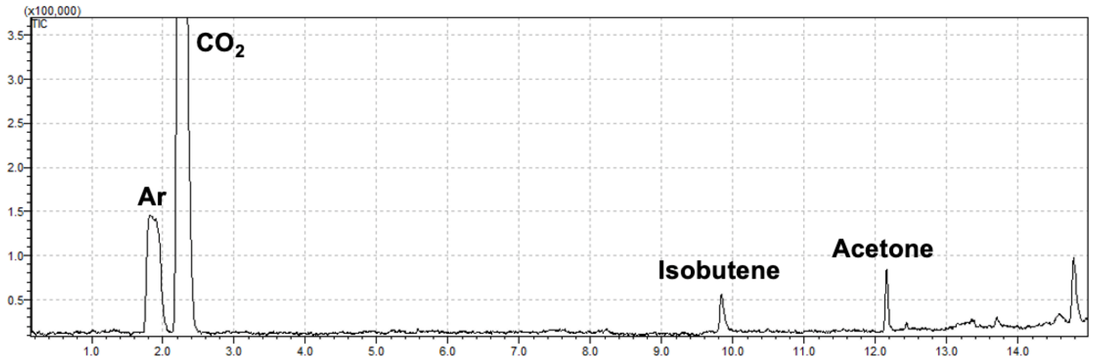

Figure S10. Representative GC-MS chromatogram of the gas phase from the reaction of Fe(OPiv)$_2$ and HOPiv with dioxygen.

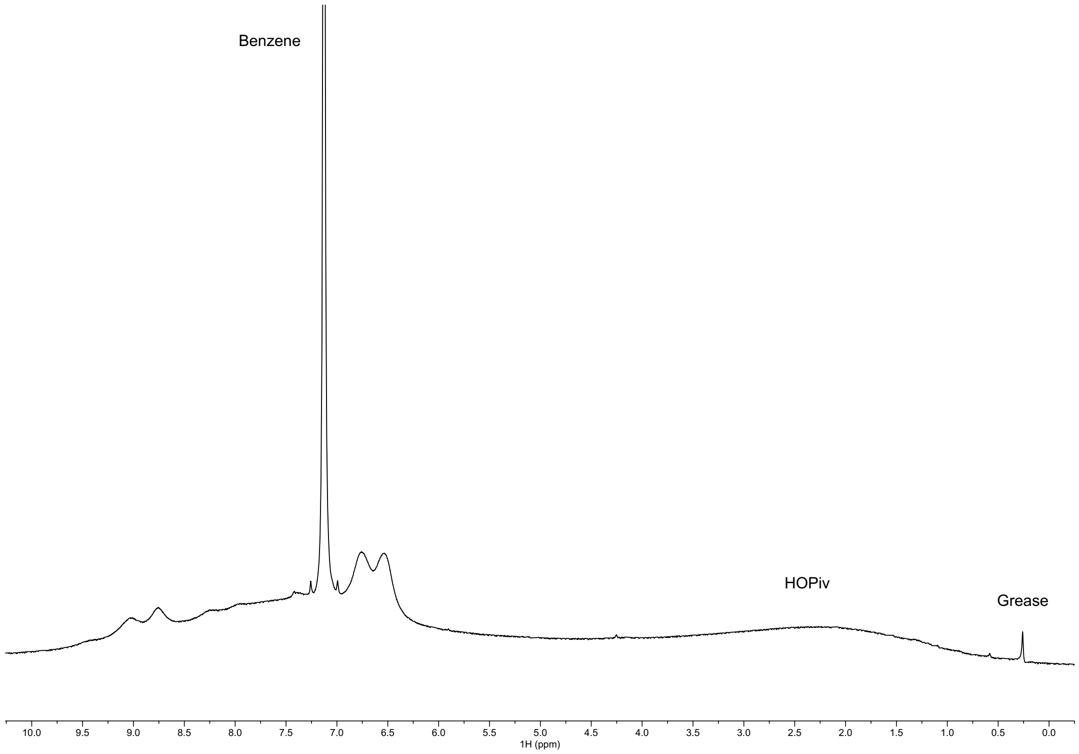

Figure S11. ^{1}H NMR spectrum of the material generated by heating $Fe(OPiv)_2$ and 2 equiv of HOPiv under 1 atm of dioxygen at 150 °C for 2 hours. The reaction was performed in C_6H_6 and the solvent was subsequently removed *in vacuo*, and the material dissolved in C_6D_6.

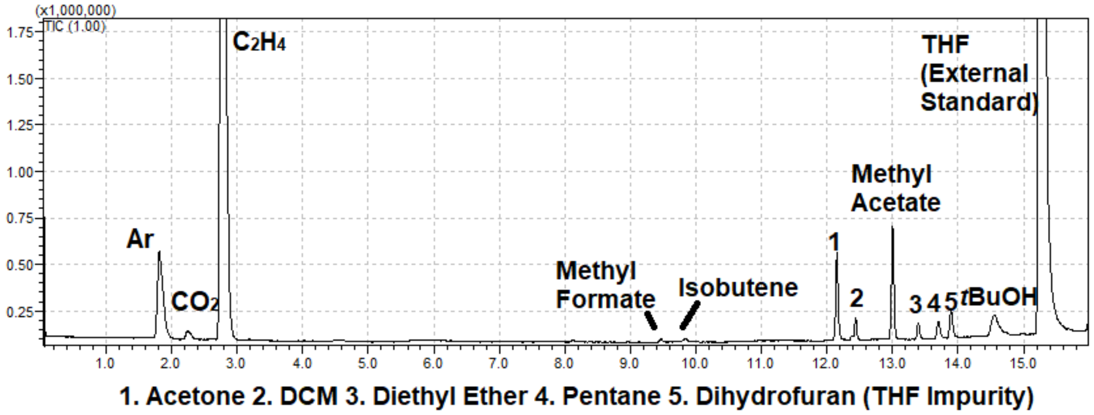

Figure S12. Representative GC-MS chromatogram for the liquid phase of the reaction of $Fe(OAc)_2$ and 2 equiv HOPiv with dioxygen. This reaction was performed in *tert*-butylbenzene to allow for cooling of the reaction to -50 °C to condense gaseous and volatile liquid products.

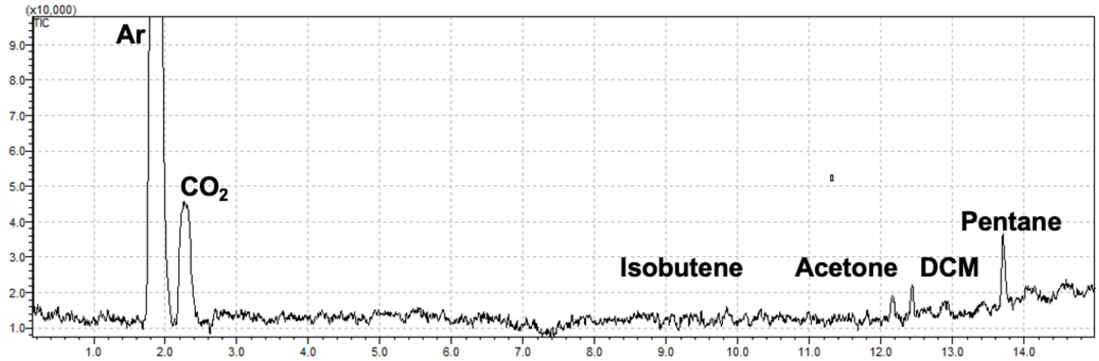

Figure S13. Representative GC-MS chromatogram for the gas phase from the reaction of $Fe^{III}_6(\mu\text{-}OH)_2(\mu_3\text{-}O)_2(\mu\text{-}OPiv)_{12}(HOPiv)_2$ and 12 equiv of HOPiv heated anaerobically at 150 °C for two hours.

Procedure for characterization of the material generated from the reaction of HOPiv and $Fe^{III}_6(\mu_4\text{-}O_2)(\mu_3\text{-}O)_2(\mu\text{-}OPiv)_{12}(HOPiv)_2$ under anaerobic conditions. Under an atmosphere of dry dinitrogen, 10 mL vials equipped with stir bars were charged with 7.5 mL (84.6 mmol) benzene, HOPiv (0.960 mol% relative to benzene, 82.9 mg, 0.812 mmol) and $Fe^{III}_6(\mu_4\text{-}O_2)(\mu_3\text{-}O)_2(\mu\text{-}OPiv)_{12}(HOPiv)_2$ (0.080 mol% relative to benzene, 122.9 mg, 0.0680 mmol). The vials were inserted into stainless steel reactors which were then pressurized with 70 psig of dinitrogen and heated on an aluminum heating block on a hot plate at 150 °C for two hours. After cooling to room temperature, the gas phase was collected in Tedlar bags for GC-MS analysis. 1 mL aliquots of the reaction solutions were diluted in benzene to a total volume of 5 mL, and UV-Visible spectroscopy was performed.

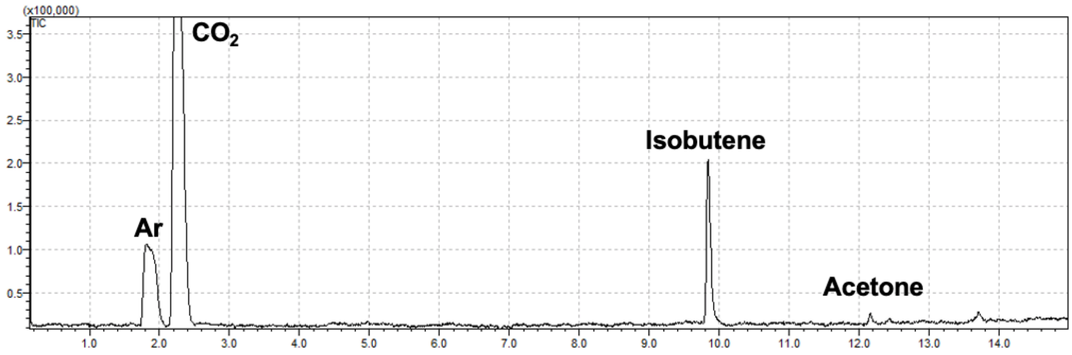

Figure S14. Representative GC-MS chromatogram for the gas phase from the reaction of $Fe^{III}_6(\mu_4\text{-}O_2)(\mu_3\text{-}O)_2(\mu\text{-}OPiv)_{12}(HOPiv)_2$ and 12 equiv of HOPiv heated anaerobically at 150 °C for two hours.

Procedure for ^{1}H NMR characterization of the material generated from the reaction of HOPiv and $Fe^{III}_6(\mu_4\text{-}O_2)(\mu_3\text{-}O)_2(\mu\text{-}OPiv)_{12}(HOPiv)_2$. Under an atmosphere of dry dinitrogen, three J-Young NMR tubes were charged with 0.3 mL benzene-d_6, HOPiv (0.960 mol% relative to benzene, 3.3 mg, 0.032 mmol) and $Fe^{III}_6(\mu_4\text{-}O_2)(\mu_3\text{-}O)_2(\mu\text{-}OPiv)_{12}(HOPiv)_2$ (0.080 mol% relative to benzene-d_6, 4.9 mg, 0.00027 mmol). The tubes were sealed and pressurized with 50 psig of dinitrogen and heated in an oil bath at 150 °C for two hours. After cooling to room temperature, ^{1}H NMR spectroscopy analysis was performed.

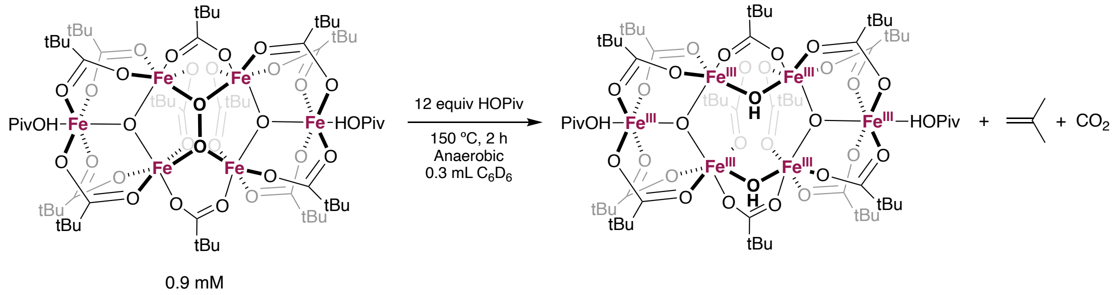

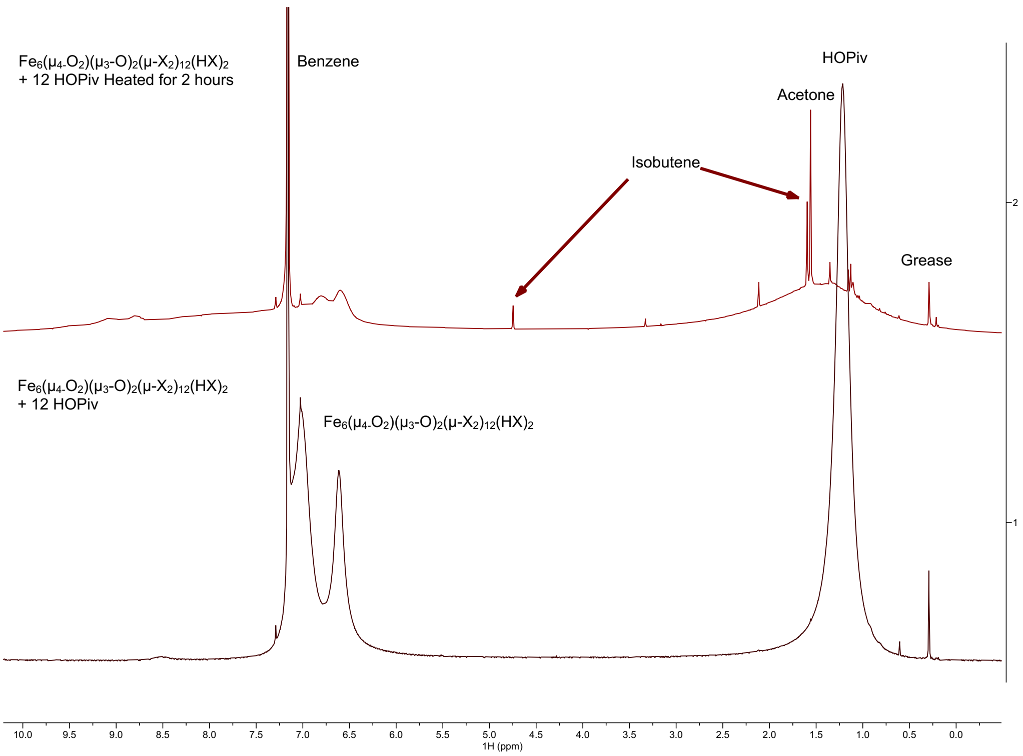

Figure S15. *in situ* ^{1}H NMR spectra of $Fe^{III}_6(\mu_4\text{-}O_2)(\mu_3\text{-}O)_2(\mu\text{-}OPiv)_{12}(HOPiv)_2$ and 12 equiv of HOPiv and its conversion to $Fe_6(\mu\text{-}OH)_2(\mu_3\text{-}O)_2(\mu\text{-}OPiv)_{12}(HOPiv)_2$ upon heating at 150 °C for two hours under anaerobic conditions.

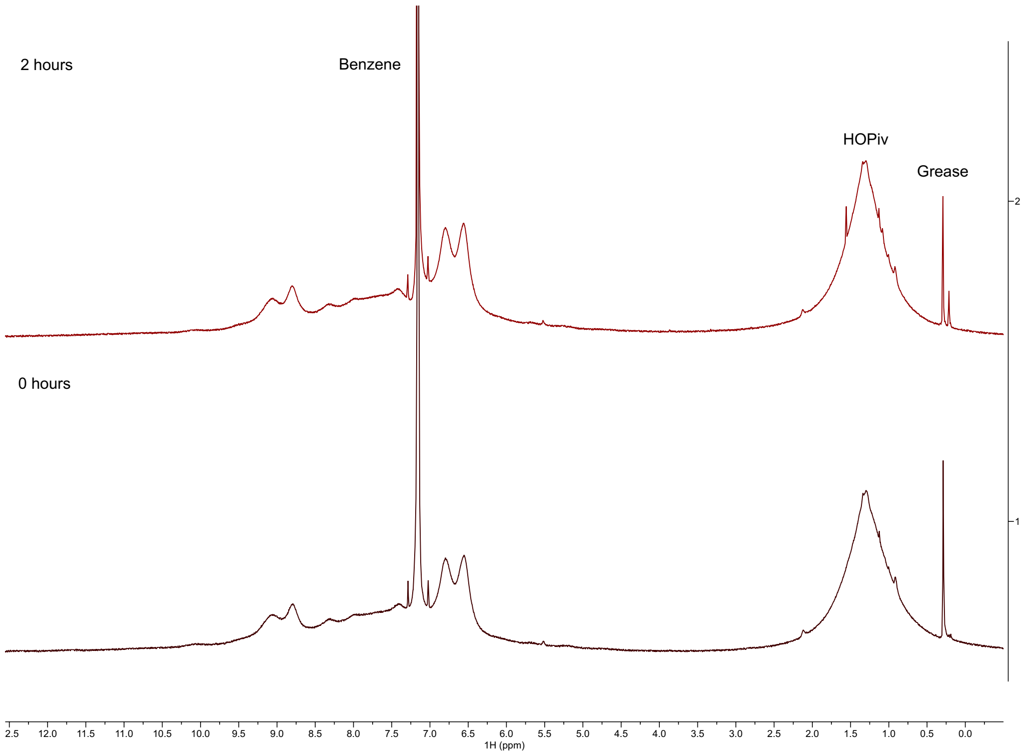

Figure S16. *in situ* ^{1}H NMR spectra of $Fe_6(\mu\text{-}OH)_2(\mu_3\text{-}O)_2(\mu\text{-}OPiv)_{12}(HOPiv)_2$ and 12 equiv of HOPiv before and after heating at 150 °C for two hours under anaerobic conditions.

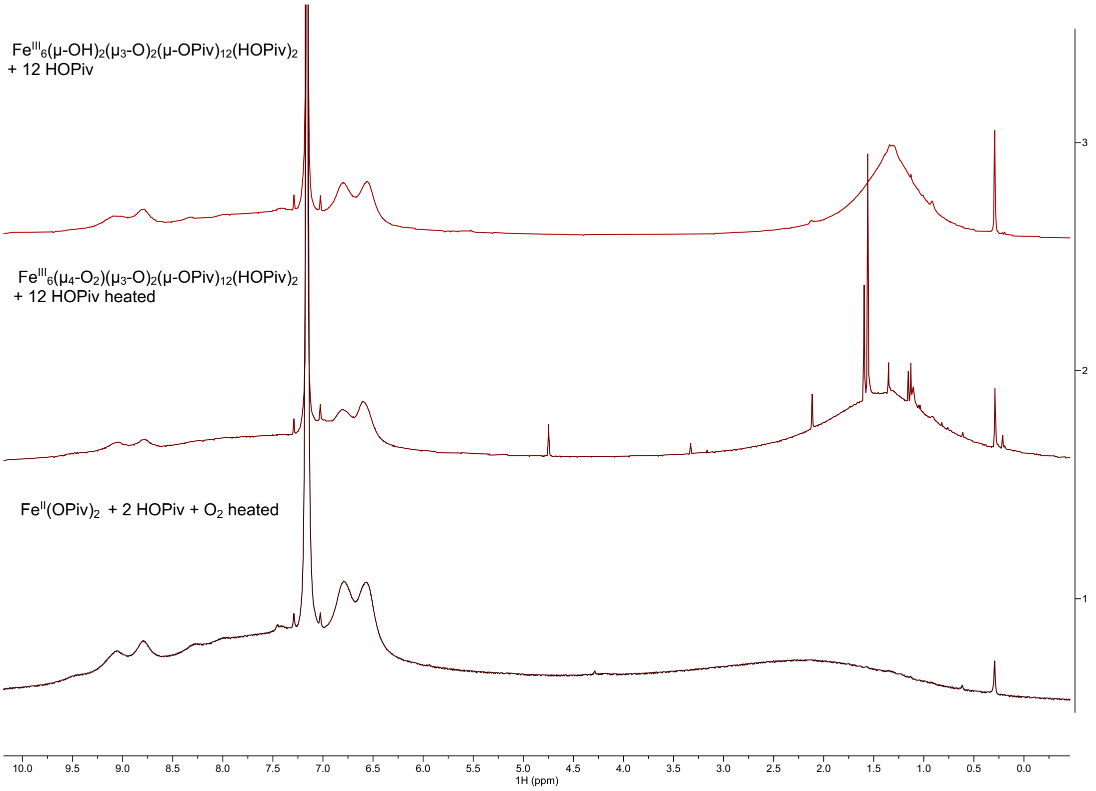

Figure S17. Stacked ^{1}H NMR spectra of (1) material generated by heating $Fe(OPiv)_2$ and 2 equiv of HOPiv under 1 atm of dioxygen at 150 °C for 2 hours in C_6D_6 and removing the solvent, (2) material generated by heating of $Fe^{III}_6(\mu_4\text{-}O_2)(\mu_3\text{-}O)_2(\mu\text{-}OPiv)_{12}(HOPiv)_2$ and 12 equiv of HOPiv under anaerobic conditions at 150 °C in a J-Young tube in an *in situ* ^{1}H NMR experiment, (3) $Fe^{III}_6(\mu\text{-}OH)_2(\mu_3\text{-}O)_2(\mu\text{-}OPiv)_{12}(HOPiv)_2$ and 12 equiv of HOPiv.

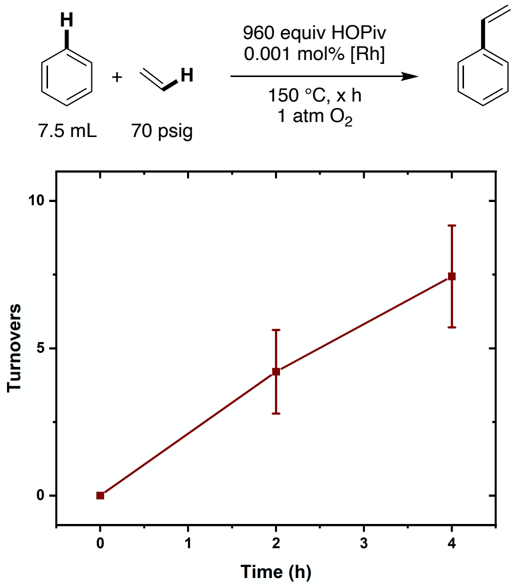

Figure S18. Turnovers versus time plot for aerobic benzene ethenylation reactions performed in the absence of Fe additive with only dioxygen as the oxidant. Reaction conditions: 7.5 mL benzene, 0.001 mol% (relative to benzene per single Rh atom) [(η^2-C_2H_4)$_2$Rh(μ-OAc)], 960 equiv HOPiv, 70 psig ethylene, 1 atm dioxygen, 150 °C. All data points reflect the average of a minimum of three independent reactions and error bars represent the standard deviation from the multiple independent experiments.

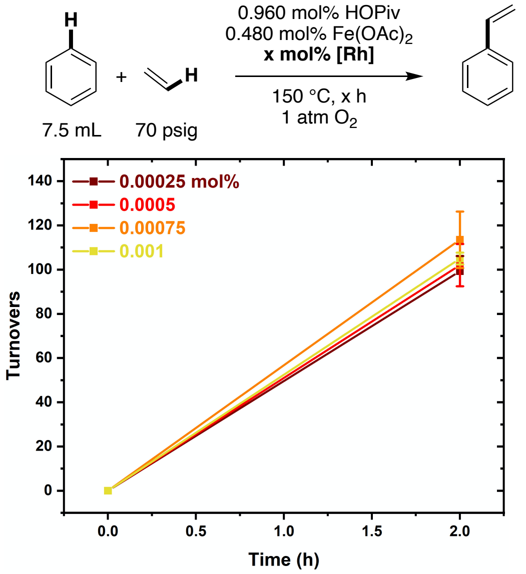

Figure S19 Turnovers versus time plots for aerobic benzene ethenylation reactions at varying [(η^2-C_2H_4)$_2$Rh(μ-OAc)]$_2$ concentration. Reaction conditions: 7.5 mL benzene, x mol% (relative to benzene per single Rh atom) [(η^2-C_2H_4)$_2$Rh(μ-OAc)], 0.480 mol% $Fe(OAc)_2$, 0.960 mol% HOPiv, 70 psig ethylene, 1 atm dioxygen, 150 °C. All data points reflect the average of a minimum of three independent reactions and error bars represent the standard deviation from the multiple independent experiments.

Procedure for aerobic benzene ethenylation at varying [(η^2-C_2H_4)$_2$Rh(μ-OAc)]$_2$ concentration. Under an atmosphere of dry dinitrogen, three 10 mL vials with stir bars were charged with 7.5 mL (84.6 mmol) benzene, [(η^2-C_2H_4)$_2$Rh(μ-OAc)]$_2$ (0.00025, 0.0005, 0.00075 or 0.001 mol% relative to benzene per single Rh atom), Fe(OAc)$_2$ (0.480 mol% relative to benzene, 69.9 mg, 0.406 mmol) and HOPiv (0.960 mol% relative to benzene, 82.9 mg, 0.812 mmol). The vials were inserted into stainless steel reactors, which were subsequently sealed. The headspace of the stainless-steel reactors was flushed with dioxygen by pressurizing with 15 psig of dioxygen and releasing the pressure six times, leaving 1 atm (0 psig) of dioxygen in the reactors. The reactors were subsequently pressurized with 70 psig of ethylene and heated in an aluminum block on a hot plate at 170 °C for two hours. Upon cooling to room temperature, reactors were sampled in air using a long needle. Next, 50 μL aliquots of the reaction mixtures were combined with 50 μL of a 11.1 mM hexamethylbenzene benzene solution and diluted in 0.25 mL of benzene to give 1000, 200, 133 or 100 equiv of external standard hexamethylbenzene. The benzene solutions were washed with a saturated aqueous solution of NaOH (1.5 mL) to remove Fe complexes and HOPiv, and the organic layer was analyzed by GC-MS.

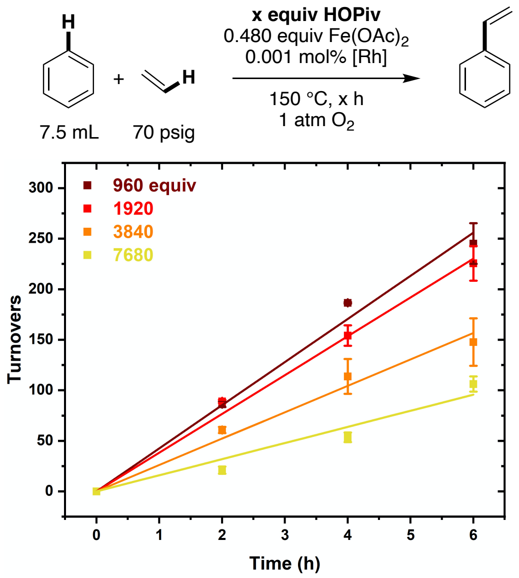

Figure S20. Turnovers versus time plots for aerobic benzene ethenylation reactions at varying HOPiv concentration. Reaction conditions: 7.5 mL benzene, 0.001 mol% (relative to benzene per single Rh atom) $[(\eta^2\text{-}C_2H_4)_2Rh(\mu\text{-}OAc)]$, 480 equiv $Fe(OAc)_2$, x equiv HOPiv, 70 psig ethylene, 1 atm dioxygen, 150 °C. All data points reflect the average of a minimum of three independent reactions and error bars represent the standard deviation from the multiple independent experiments.

Procedure for aerobic benzene ethenylation at varying HOPiv concentration. Under an atmosphere of dry dinitrogen, three 10 mL vials with stir bars were charged with 7.5 mL (84.6 mmol) benzene, $[(\eta^2\text{-}C_2H_4)_2Rh(\mu\text{-}OAc)]_2$ (0.001 mol% relative to benzene per single Rh atom, 0.18 mg, 0.846 µmol), $Fe(OAc)_2$ (480 equiv relative to Rh, 69.9 mg, 0.406 mmol) and HOPiv (960, 1920, 3840 or 7680 equiv). The vials were inserted into stainless steel reactors, which were subsequently sealed. The headspaces of the stainless-steel reactors were flushed with dioxygen by pressurizing with 15 psig of dioxygen and releasing the pressure six times, leaving 1 atm (0 psig) of dioxygen in the reactors. The reactors were subsequently pressurized with 70 psig of ethylene and heated in an aluminum block on a hot plate at 170 °C for two hours. Upon cooling to room temperature, reactors were sampled in air using a long needle. Next, 50 µL aliquots of the reaction mixtures were combined with 50 µL of a 11.1 mM

hexamethylbenzene benzene solution and diluted in 0.25 mL of benzene to give 100 equiv of external standard hexamethylbenzene. The benzene solutions were washed with a saturated aqueous solution of NaOH (1.5 mL) to remove Fe complexes and HOPiv, and the organic layer was analyzed by GC-MS.

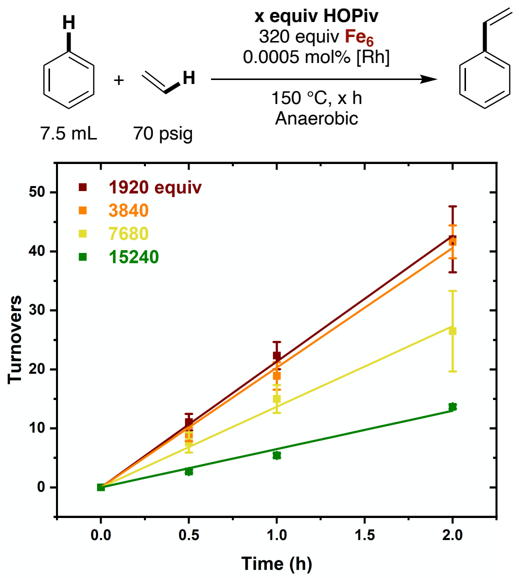

Figure S21. Turnovers versus time plots for anaerobic benzene ethenylation reactions at varying HOPiv concentration. Reaction conditions: 7.5 mL benzene, 0.0005 mol% (relative to benzene per single Rh atom) $[(\eta^2\text{-}C_2H_4)_2Rh(\mu\text{-}OAc)]_2$, x equiv HOPiv (relative to Rh), 70 psig ethylene, 80 psig dinitrogen, 320 equiv Fe_6, 150 °C. Fe_6 = $Fe^{III}_6(\mu\text{-}OH)_2(\mu_3\text{-}O)_2(\mu\text{-}X)_{12}(HX)_2$ which was prepared *in situ* by heating 0.81 mmol of $Fe(OAc)_2$ and 1.62 mmol of HOPiv at 150 °C in 5 mL of benzene under 3 atm of dioxygen for 2 hours. All data points reflect the average of a minimum of three independent reactions and error bars represent the standard deviation from the multiple independent experiments.

Procedure for anaerobic benzene ethenylation kinetics at varying HOPiv concentration. Under an atmosphere of dry dinitrogen, three 10 mL vials with stir bars were charged with 5 mL (84.6 mmol) benzene, $[(\eta^2\text{-}C_2H_4)_2Rh(\mu\text{-}OAc)]_2$ (0.001 mol% relative to benzene per single Rh atom, 0.18 mg, 0.846 µmol), $Fe(OAc)_2$ (139.7 mg, 0.812 mmol), and HOPiv (165.7 mg, 1.62 mmol). The vials were inserted into previously described stainless steel reactors. The reactors were subsequently

pressurized with 70 psig of dinitrogen and 45 psig of dioxygen and heated in an aluminum block on a hot plate at 150 °C for two hours. The quantity of [(η^2-C_2H_4)$_2$Rh(μ-OAc)]$_2$ for three reactions (0.276 mg, 1.27 μmol) was added to 7.5 mL of benzene and the appropriate quantity of additional HOPiv, and 2.5 mL aliquots of this solution were added to each of the three stainless steel reactors. Dioxygen was removed from the reactors by cycling between dinitrogen pressure and partial vacuum on a high-pressure line, and the reactors were subsequently pressurized with 70 psig of ethylene. The reactors were heated in an aluminum block on a hot plate for 30-minute intervals for the first hour, and again after two hours. Next, 50 μL aliquots of the reaction mixtures were combined with 50 μL of and 11.1 mM benzene solution of hexamethylbenzene in 0.25 mL of benzene to give 200 equiv of external standard hexamethylbenzene. The benzene solutions were washed with a saturated aqueous solution of NaOH (1.5 mL) to remove Fe salts and carboxylic acid, and the organic layer was analyzed by GC-MS.

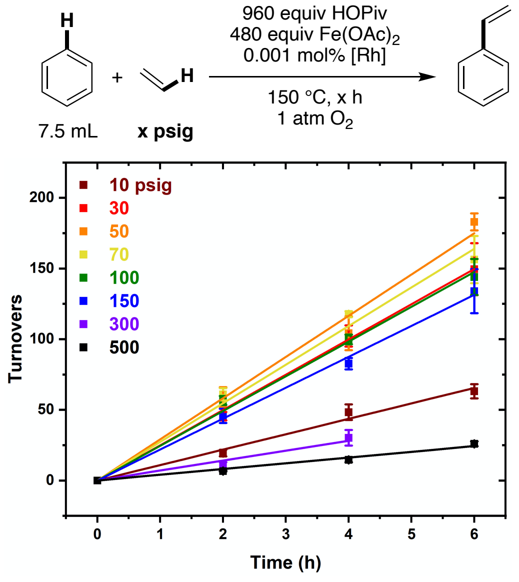

Figure S22. Turnovers versus time plots for aerobic benzene ethenylation reactions at varying ethylene pressure. Reaction conditions: 7.5 mL benzene, 0.001 mol% (relative to benzene per single Rh atom) [(η^2-C_2H_4)$_2$Rh(μ-OAc)], 480 equiv Fe(OAc)$_2$ or Cu(OPiv)$_2$, 0.960 mol% HOPiv, x psig ethylene, 1 atm dioxygen, 150 °C. All data points reflect the average of a minimum of three independent reactions and error bars represent the standard deviation from the multiple independent experiments.

Procedure for aerobic benzene ethenylation at varying ethylene concentration. Under an atmosphere of dry dinitrogen, three 10 mL vials with stir bars were charged with 7.5 mL (84.6 mmol) benzene, [(η^2-C_2H_4)$_2$Rh(μ-OAc)]$_2$ (0.001 mol% relative to benzene per single Rh atom, 0.18 mg, 0.846 μmol), Fe(OAc)$_2$ (480 equiv relative to Rh, 69.9 mg, 0.406 mmol) and HOPiv (0.960 mol% relative to benzene, 82.9 mg, 0.812 mmol). The vials were inserted into stainless steel reactors, which were subsequently sealed. The headspace of the stainless-steel reactors was flushed with dioxygen by pressurizing with 15 psig of dioxygen and releasing the pressure six times, leaving 1 atm (0 psig) of dioxygen in the reactors. The reactors were subsequently pressurized with 70 psig of ethylene and heated in an aluminum block on a hot plate at 150 °C for two-hour intervals. Upon cooling to room temperature, the reactors were sampled in air using a long needle. Next, 50 μL aliquots of the reaction mixtures were combined with 50 μL of a 11.1 mM hexamethylbenzene benzene solution and diluted in 0.25 mL of benzene to give 100 equiv of external standard hexamethylbenzene. The benzene solutions were washed with a saturated aqueous solution of NaOH (1.5 mL) to remove Fe complexes and carboxylic acid, and the organic layer was analyzed by GC-MS.

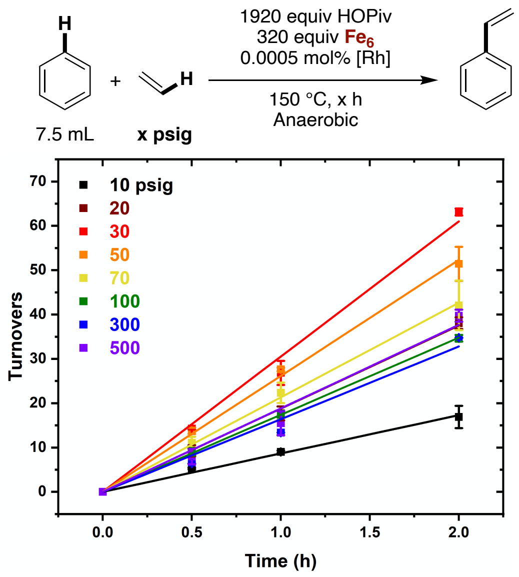

Figure S23. Turnovers versus time plots for anaerobic benzene ethenylation reactions at varying ethylene pressure. Reaction conditions: 7.5 mL benzene, 0.0005 mol% (relative to benzene per single Rh atom) $[(\eta^2\text{-}C_2H_4)_2Rh(\mu\text{-}OAc)]_2$, 1920 equiv HOPiv (relative to Rh), x psig ethylene and for ethylene pressures < 150 psig, dinitrogen pressure was added to bring the total pressure to 150 psig, 320 equiv Fe_6, 150 °C. Fe_6 = $Fe^{III}_6(\mu\text{-}OH)_2(\mu_3\text{-}O)_2(\mu\text{-}X)_{12}(HX)_2$ which was prepared *in situ* by heating 0.81 mmol of $Fe(OAc)_2$ and 1.62 mmol of HOPiv at 150 °C in 5 mL of benzene under 3 atm of dioxygen for 2 hours. All data points reflect the average of a minimum of three independent reactions and error bars represent the standard deviation from the multiple independent experiments.

Table S2. Selectivity for the arene ethenylation reaction versus ethylene oxidation side products as a function of ethylene pressure.

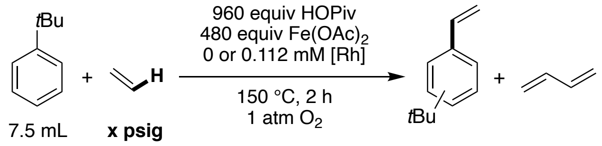

Ethylene Pressure (psig)	Butadiene (TOs)	*tert*-Butyl Styrenes (TOs)
70 (No Rh)	N.D.	N.D.
70	4.9(5)	26(1)
500	3.7(4)	6(1)

Procedure for studying selectivity for alkenyl arenes versus low boiling point ethylene oxidation products. Condensing gaseous products was necessary to obtain

reproducible data. Since the freezing point of benzene is ~5 °C, *tert*-butylbenzene was used in its place. Under an atmosphere of dry dinitrogen, three 10 mL vials with stir bars were charged with 7.5 mL (48.1 mmol) *tert*-butyl benzene, [(η^2-C_2H_4)$_2$Rh(μ-OAc)]$_2$ (if present, 0.112 mM per single Rh atom, 0.18 mg, 0.846 μmol), Fe(OAc)$_2$ (480 equiv relative to Rh, 69.9 mg, 0.406 mmol) and HOPiv (960 equiv, 82.9 mg, 0.812 mmol). The vials were inserted into stainless steel reactors, which were subsequently sealed. The headspace of the stainless-steel reactors was flushed with dioxygen by pressurizing with 15 psig of dioxygen and releasing the pressure six times, leaving 1 atm (0 psig) of dioxygen in the reactors. The reactors were subsequently pressurized with 70 or 500 psig of ethylene and heated in an aluminum block on a hot plate at 150 °C for two-hours. Upon cooling to room temperature, the reactors were cooled to -50 °C in an acetonitrile-dry ice bath. The reactors were slowly vented, and 1000 equiv of external standard tetrahydrofuran were added to the reactors while cold. 1 μL aliquots were analyzed by GC-MS to quantify butadiene production. Following butadiene quantification, 50 μL aliquots of the reaction mixtures were combined with 50 μL of a 11.1 mM hexamethylbenzene benzene solution and diluted in 0.25 mL of benzene to give 100 equiv of external standard hexamethylbenzene. The benzene solutions were washed with a saturated aqueous solution of NaOH (1.5 mL) to remove Fe complexes and carboxylic acid, and the organic layer was analyzed by GC-MS to quantify production of *tert*-butylstyrenes.

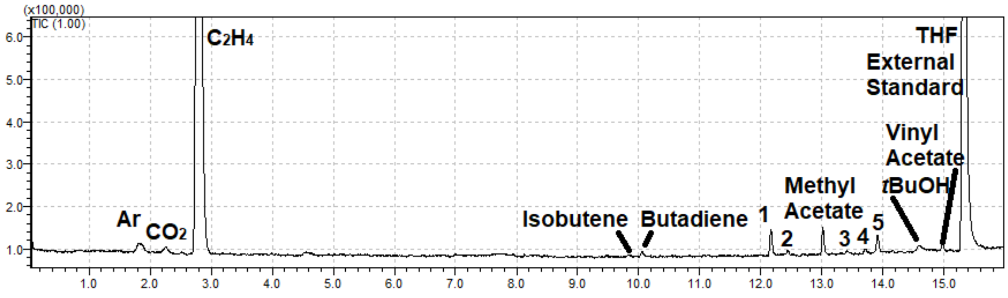

Figure S24. Representative GC-MS chromatogram used to quantify the production of butadiene in *tert*-butylbenzene solvent.

Procedure for anaerobic benzene ethenylation kinetics at varying ethylene concentration. Under an atmosphere of dry dinitrogen, three 10 mL vials with stir bars were charged with 5 mL (84.6 mmol) benzene, $Fe(OAc)_2$ (139.7 mg, 0.812 mmol), and HOPiv (165.7 mg, 1.62 mmol). The vials were inserted into previously described stainless steel reactors. The reactors were subsequently pressurized with 70 psig of dinitrogen and 45 psig of dioxygen and heated in an aluminum block on a hot plate at 150 °C for two hours. The quantity of $[(\eta^2\text{-}C_2H_4)_2Rh(\mu\text{-}OAc)]_2$ for three reactions (0.276 mg, 1.27 μmol per single Rh atom) was added to 7.5 mL of benzene, and 2.5 mL aliquots of this solution were added to each of the three stainless steel reactors. Dioxygen was removed from the reactors by cycling between dinitrogen pressure and partial vacuum on a high-pressure line, and the reactors were subsequently pressurized with 10, 20, 30, 50, 70, 100, 300 or 500 psig of ethylene. The reactors were heated in an aluminum block on a hot plate for 30-minute intervals for the first hour, and sampled again after two hours. Next, 50 μL aliquots of the reaction mixtures were combined with 50 μL of and 11.1 mM benzene solution of hexamethylbenzene in 0.25 mL of

benzene to give 200 equiv of external standard hexamethylbenzene. The benzene solutions were washed with a saturated aqueous solution of NaOH (1.5 mL) to remove Fe salts and carboxylic acid, and the organic layer was analyzed by GC-MS.

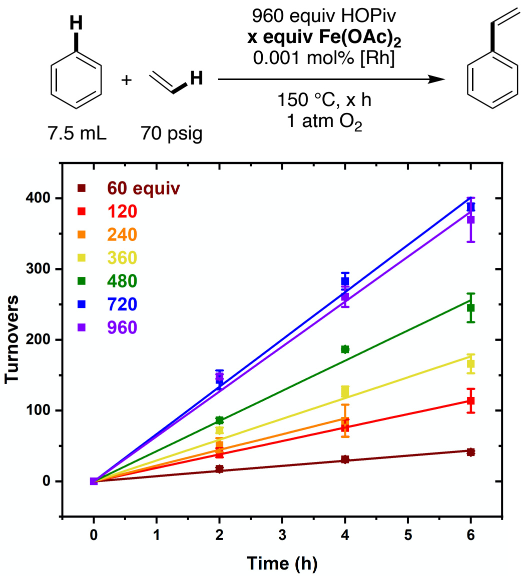

Figure S25. Turnovers versus time plots for aerobic benzene ethenylation reactions at varying $Fe(OAc)_2$ concentration. Reaction conditions: 7.5 mL benzene, 0.001 mol% (relative to benzene per single Rh atom) $[(\eta^2\text{-}C_2H_4)_2Rh(\mu\text{-}OAc)]$, x equiv $Fe(OAc)_2$ or $Cu(OPiv)_2$, 960 equiv HOPiv, 70 psig ethylene, 1 atm dioxygen, 150 °C. All data points reflect the average of a minimum of three independent reactions and error bars represent the standard deviation from the multiple independent experiments.

Procedure for aerobic benzene ethenylation at varying $Fe(OAc)_2$ concentration. Under an atmosphere of dry dinitrogen, three 10 mL vials with stir bars were charged with 7.5 mL (84.6 mmol) benzene, $[(\eta^2\text{-}C_2H_4)_2Rh(\mu\text{-}OAc)]_2$ (0.001 mol% relative to benzene per single Rh atom, 0.18 mg, 0.846 µmol), $Fe(OAc)_2$ (60, 120, 360, 480, 720 or 960 equiv relative to single Rh atom) and HOPiv (960 equiv relative to single Rh atom, 82.9 mg, 0.812 mmol). The vials were inserted into stainless steel reactors, which were subsequently sealed. The headspace of the stainless-steel reactors was flushed with dioxygen by pressurizing with 15 psig of dioxygen and releasing the

pressure six times, leaving 1 atm (0 psig) of dioxygen in the reactors. The reactors were subsequently pressurized with 70 psig of ethylene and heated in an aluminum block on a hot plate at 170 °C for two hours. Upon cooling to room temperature, reactors were sampled in air using a long needle. Next, 50 μL aliquots of the reaction mixtures were combined with 50 μL of a 11.1 mM hexamethylbenzene benzene solution and diluted in 0.25 mL of benzene to give 1000, 200, 133 or 100 equiv of external standard hexamethylbenzene. The benzene solutions were washed with a saturated aqueous solution of NaOH (1.5 mL) to remove Fe complexes and HOPiv, and the organic layer was analyzed by GC-MS.

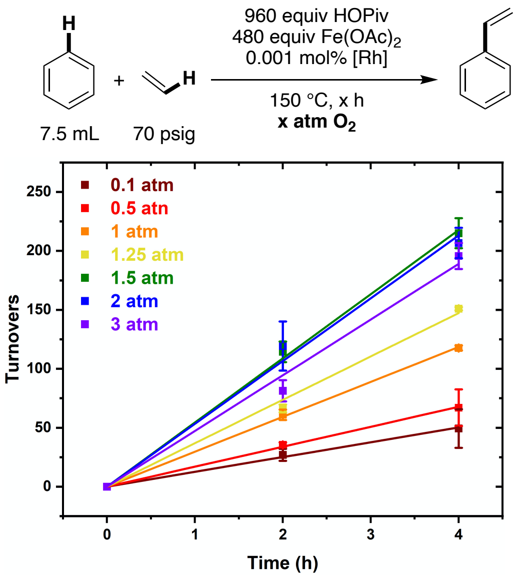

Figure S26. Turnovers versus time plots for aerobic benzene ethenylation reactions at varying dioxygen pressure. Reaction conditions: 7.5 mL benzene, 0.001 mol% (relative to benzene per single Rh atom) $[(\eta^2\text{-}C_2H_4)_2Rh(\mu\text{-}OAc)]$, 480 equiv $Fe(OAc)_2$ or $Cu(OPiv)_2$, 960 equiv HOPiv, 70 psig ethylene, x atm dioxygen, 150 °C. All data points reflect the average of a minimum of three independent reactions and error bars represent the standard deviation from the multiple independent experiments.

Procedure for aerobic benzene ethenylation at varying dioxygen pressure. Under an atmosphere of dry dinitrogen, three 10 mL vials with stir bars were charged with 7.5 mL (84.6 mmol) benzene, $[(\eta^2\text{-}C_2H_4)_2Rh(\mu\text{-}OAc)]_2$ (0.001 mol% relative to benzene per single Rh atom, 0.18 mg, 0.846 μmol per single Rh atom), $Fe(OAc)_2$ (60, 120, 360, 480, 720 or 960 equiv relative to single rh atom) and HOPiv (960 equiv relative to single Rh atom, 82.9 mg, 0.812 mmol). The vials were inserted into stainless steel reactors, which were subsequently sealed. The reactors were subsequently pressurized with 70 psig of ethylene and 0.1, 0.5, 1, 1.25, 1.5, 2, or 3 atm of dioxygen and heated in an aluminum block on a hot plate at 170 °C for two hours. Upon cooling to room temperature, reactors were sampled in air using a long needle. Next, 50 μL aliquots of the reaction mixtures were combined with 50 μL of a 11.1 mM hexamethylbenzene benzene solution and diluted in 0.25 mL of benzene to give 100 equiv of external standard hexamethylbenzene. The benzene solutions were washed with a saturated aqueous solution of NaOH (1.5 mL) to remove Fe complexes and carboxylic acid, and the organic layer was analyzed by GC-MS.

Procedure for studying aerobic benzene ethenylation catalyst longevity at varying ethylene pressure. Under an atmosphere of dry dinitrogen, three 10 mL vials with stir bars were charged with 7.5 mL (84.6 mmol) benzene, $[(\eta^2\text{-}C_2H_4)_2Rh(\mu\text{-}OAc)]_2$ (0.001 mol% relative to benzene per single Rh atom, 0.18 mg, 0.846 μmol per single Rh atom), $Fe(OAc)_2$ (480 equiv relative to Rh, 69.9 mg, 0.406 mmol) and HOPiv (960 mol% relative to benzene, 82.9 mg, 0.812 mmol). The vials were inserted into stainless steel reactors, which were subsequently sealed. The reactors were subsequently

pressurized with 70, 120 or 500 psig of ethylene and 3 atm of dioxygen and heated in an aluminum block on a hot plate at 170 °C for two hours. Upon cooling to room temperature, reactors were sampled in air using a long needle. Next, 50 μL aliquots of the reaction mixtures were combined with 50 μL of a 11.1 mM hexamethylbenzene benzene solution and diluted in 0.25 mL of benzene to give 100 equiv of external standard hexamethylbenzene. The benzene solutions were washed with a saturated aqueous solution of NaOH (1.5 mL) to remove Fe complexes and carboxylic acid, and the organic layer was analyzed by GC-MS.

Procedure for studying aerobic benzene ethenylation catalyst longevity at varying HOPiv concentration. Under an atmosphere of dry dinitrogen, three 10 mL vials with stir bars were charged with 7.5 mL (84.6 mmol) benzene, $[(\eta^2\text{-}C_2H_4)_2Rh(\mu\text{-}OAc)]_2$ (0.001 mol% relative to benzene per single Rh atom, 0.18 mg, 0.846 μmol per single Rh atom), $Fe(OAc)_2$ (0.480 mol% relative to benzene, 69.9 mg, 0.406 mmol) and HOPiv (0, 0.960 or 3.840 mol% relative to benzene). The vials were inserted into stainless steel reactors, which were subsequently sealed. The reactors were subsequently pressurized with 70 psig of ethylene and 3 atm of dioxygen and heated in an aluminum block on a hot plate at 170 °C for two hours. Upon cooling to room temperature, reactors were sampled in air using a long needle. Next, 50 μL aliquots of the reaction mixtures were combined with 50 μL of a 11.1 mM hexamethylbenzene benzene solution and diluted in 0.25 mL of benzene to give 100 equiv of external standard hexamethylbenzene. The benzene solutions were washed with a saturated

aqueous solution of NaOH (1.5 mL) to remove Fe complexes and carboxylic acid, and the organic layer was analyzed by GC-MS.

Procedure for studying aerobic benzene ethenylation catalyst longevity at varying $Fe(OAc)_2$ concentration. Under an atmosphere of dry dinitrogen, three 10 mL vials with stir bars were charged with 7.5 mL (84.6 mmol) benzene, $[(\eta^2\text{-}C_2H_4)_2Rh(\mu\text{-}OAc)]_2$ (0.001 mol% relative to benzene per single Rh atom, 0.18 mg, 0.846 μmol per single Rh atom), $Fe(OAc)_2$ (0.240, 0.480 or 0.960 mol% relative to benzene) and HOPiv (960 mol% relative to benzene, 82.9 mg, 0.812 mmol). The vials were inserted into stainless steel reactors, which were subsequently sealed. The reactors were subsequently pressurized with 70 psig of ethylene and 3 atm of dioxygen and heated in an aluminum block on a hot plate at 170 °C for two hours. Upon cooling to room temperature, reactors were sampled in air using a long needle. Next, 50 μL aliquots of the reaction mixtures were combined with 50 μL of a 11.1 mM hexamethylbenzene benzene solution and diluted in 0.25 mL of benzene to give 100 equiv of external standard hexamethylbenzene. The benzene solutions were washed with a saturated aqueous solution of NaOH (1.5 mL) to remove Fe complexes and carboxylic acid, and the organic layer was analyzed by GC-MS.

Procedure for studying aerobic benzene ethenylation catalyst longevity at varying $[(\eta^2\text{-}C_2H_4)_2Rh(\mu\text{-}OAc)]_2$ concentration. Under an atmosphere of dry dinitrogen, three 10 mL vials with stir bars were charged with 7.5 mL (84.6 mmol) benzene, $[(\eta^2\text{-}C_2H_4)_2Rh(\mu\text{-}OAc)]_2$ (0.0001, 0.0005, 0.001 or 0.002 mol% relative to benzene per single Rh atom), $Fe(OAc)_2$ (0.480 mol% relative to benzene, 69.9 mg,

0.406 mmol) and HOPiv (960 mol% relative to benzene, 82.9 mg, 0.812 mmol). The vials were inserted into stainless steel reactors, which were subsequently sealed. The reactors were subsequently pressurized with 70 psig of ethylene and 3 atm of dioxygen and heated in an aluminum block on a hot plate at 170 °C for two hours. Upon cooling to room temperature, reactors were sampled in air using a long needle. Next, 50 μL aliquots of the reaction mixtures were combined with 50 μL of a 11.1 mM hexamethylbenzene benzene solution and diluted in 0.25 mL of benzene to give 1000, 200, 100 or 50 equiv of external standard hexamethylbenzene. The benzene solutions were washed with a saturated aqueous solution of NaOH (1.5 mL) to remove Fe complexes and carboxylic acid, and the organic layer was analyzed by GC-MS.

Discussion on the Effects of Water, Benzaldehyde or Styrene Additive. The effects of water, styrene and benzaldehyde, which are the most prominent products of the reaction, on catalyst longevity were studied. Figure S27a shows results from reaction mixtures that were spiked with styrene. The Figure plots TOs of styrene versus time <u>with the initial loading of styrene subtracted</u>. The results indicate that the initial reaction rate is substantially decreased as initial styrene loading is increased, while the maximum additional TOs of styrene produced are decreased. As shown in Figure S27b, the addition of 300 equiv of benzaldehyde produces no statistically significant difference in the initial reaction rate and catalyst longevity. With 600 equiv of benzaldehyde, the initial reaction rate is suppressed, and the total TOs after 16 hours are decreased by one-half; however, 600 equiv of benzaldehyde is ~4-fold higher than the quantity of benzaldehyde produced after 16 hours under typical reaction conditions.

The addition of 480 and 960 equiv of water results in a slight decrease in both reaction rate and catalyst longevity (Figure S27c); however, the effect is insignificant when compared to the inhibitive effect of styrene additive. Taken together, the results in Figure S27 are consistent with the accumulation of styrene product being the most significant factor that limits the longevity of catalysis. As discussed above, high ethylene pressures (>150 psig) inhibit the reaction rate, so it is likely that styrene inhibits the reaction by a similar mechanism to ethylene

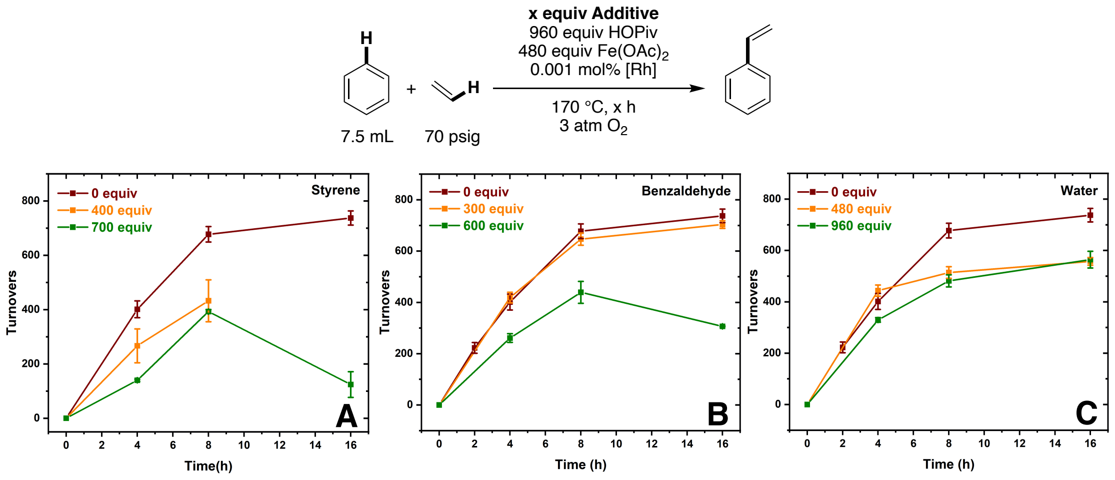

Figure S27. TOs versus time plots at varying water, styrene and benzaldehyde concentrations. Reaction conditions: 7.5 mL benzene, 0.001 mol% (relative to benzene per single Rh atom) $[(\eta^2\text{-}C_2H_4)_2Rh(\mu\text{-}OAc)]_2$, 480 equiv (per single Rh atom) $Fe(OAc)_2$, 960 equiv HOPiv, x equiv water, benzaldehyde or styrene additive, 70 psig ethylene, 3 atm dioxygen, 170 °C. All data points reflect the average of a minimum of three independent reactions and error bars represent the standard deviation from the multiple independent experiments.

Procedure for studying aerobic benzene ethenylation catalyst longevity in the presence of added styrene. Under an atmosphere of dry dinitrogen, three 10 mL vials with stir bars were charged with 7.5 mL (84.6 mmol) benzene, $[(\eta^2\text{-}C_2H_4)_2Rh(\mu\text{-}OAc)]_2$ (0.001 mol% relative to benzene per single Rh atom, 0.18 mg, 0.846 μmol per single Rh atom), $Fe(OAc)_2$ (480 equiv relative to Rh, 69.9 mg, 0.406 mmol), HOPiv (960

equiv relative to Rh, 82.9 mg, 0.812 mmol) and styrene (0, 300 or 500 equiv relative to Rh). The vials were inserted into stainless steel reactors, which were subsequently sealed. The initial loading of styrene was verified by GC-MS analysis. The reactors were subsequently pressurized with 70 psig of ethylene and 3 atm of dioxygen and heated in an aluminum block on a hot plate at 170 °C. Upon cooling to room temperature, reactors were sampled in air using a long needle. Next, 50 μL aliquots of the reaction mixtures were combined with 50 μL of a 11.1 mM hexamethylbenzene benzene solution and diluted in 0.25 mL of benzene to give 100 equiv of external standard hexamethylbenzene. The benzene solutions were washed with a saturated aqueous solution of NaOH (1.5 mL) to remove Fe complexes and carboxylic acid, and the organic layer was analyzed by GC-MS.

Procedure for studying aerobic benzene ethenylation catalyst longevity in the presence of added benzaldehyde. Under an atmosphere of dry dinitrogen, three 10 mL vials with stir bars were charged with 7.5 mL (84.6 mmol) benzene, $[(\eta^2\text{-}C_2H_4)_2Rh(\mu\text{-}OAc)]_2$ (0.001 mol% relative to benzene per single Rh atom, 0.18 mg, 0.846 μmol per single Rh atom), $Fe(OAc)_2$ (480 equiv relative to Rh, 69.9 mg, 0.406 mmol), HOPiv (960 equiv relative to Rh, 82.9 mg, 0.812 mmol) and benzaldehyde (0, 300 or 600 equiv relative to Rh). The vials were inserted into stainless steel reactors, which were subsequently sealed. The initial loading of benzaldehyde was verified by GC-MS analysis. The reactors were subsequently pressurized with 70 psig of ethylene and 3 atm of dioxygen and heated in an aluminum block on a hot plate at 170 °C. Upon cooling to room temperature, reactors were sampled in air using a long needle. Next,

50 μL aliquots of the reaction mixtures were combined with 50 μL of a 11.1 mM hexamethylbenzene benzene solution and diluted in 0.25 mL of benzene to give 100 equiv of external standard hexamethylbenzene. The benzene solutions were washed with a saturated aqueous solution of NaOH (1.5 mL) to remove Fe complexes and carboxylic acid, and the organic layer was analyzed by GC-MS.

Procedure for studying aerobic benzene ethenylation catalyst longevity in the presence of added water. Under an atmosphere of dry dinitrogen, three 10 mL vials with stir bars were charged with 7.5 mL (84.6 mmol) benzene, $[(\eta^2\text{-}C_2H_4)_2Rh(\mu\text{-}OAc)]_2$ (0.001 mol% relative to benzene per single Rh atom, 0.18 mg, 0.846 μmol per single Rh atom), $Fe(OAc)_2$ (480 equiv relative to Rh, 69.9 mg, 0.406 mmol), HOPiv (960 equiv relative to Rh, 82.9 mg, 0.812 mmol) and water (0, 480 or 960 equiv relative to Rh). The vials were inserted into stainless steel reactors, which were subsequently sealed. The reactors were subsequently pressurized with 70 psig of ethylene and 3 atm of dioxygen and heated in an aluminum block on a hot plate at 170 °C. Upon cooling to room temperature, reactors were sampled in air using a long needle. Next, 50 μL aliquots of the reaction mixtures were combined with 50 μL of a 11.1 mM hexamethylbenzene benzene solution and diluted in 0.25 mL of benzene to give 100 equiv of external standard hexamethylbenzene. The benzene solutions were washed with a saturated aqueous solution of NaOH (1.5 mL) to remove Fe complexes and carboxylic acid, and the organic layer was analyzed by GC-MS.

Discussion of Styrene Oxidation to Benzaldehyde. We probed whether the rate of styrene oxidation to benzaldehyde, the most significant side product, is enhanced in

the presence of $[(\eta^2\text{-}C_2H_4)_2Rh(\mu\text{-}OAc)]_2$ or $Fe(OAc)_2$. To do so, we studied styrene oxidation in benzene under 3 atm of dioxygen with a 0.960 mol% loading of styrene (relative to benzene) and a 0.960 mol% loading of HOPiv in the presence of either no additive, 0.001 mol% of $[(\eta^2\text{-}C_2H_4)_2Rh(\mu\text{-}OAc)]_2$, or 0.960 mol% of $Fe(OAc)_2$. As shown in Figure S28, benzaldehyde formation occurs slowly in the absence of $[(\eta^2\text{-}C_2H_4)_2Rh(\mu\text{-}OAc)]_2$ or $Fe(OAc)_2$. The addition of 0.001 mol% of $[(\eta^2\text{-}C_2H_4)_2Rh(\mu\text{-}OAc)]_2$ results in a benzaldehyde production rate that is ~5-fold faster, and the addition of 0.480 mol% of $Fe(OAc)_2$ results in a rate that is ~20-fold faster than the reaction in the absence of additives.

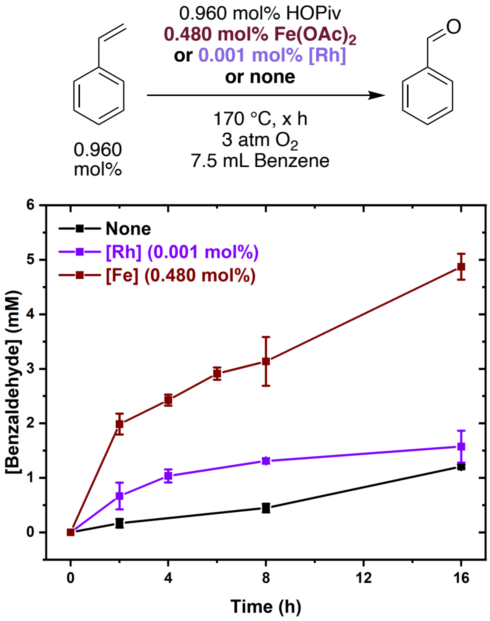

Figure S28. Kinetics of styrene oxidation to benzaldehyde. Reaction conditions: 7.5 mL benzene, 0.960 mol% HOPiv, 0.960 mol% styrene, 3 atm dioxygen, and either (1) no metal additive, (2) 0.001 mol% $[(\eta^2\text{-}C_2H_4)_2Rh(\mu\text{-}OAc)]_2$ (relative to benzene per single Rh atom), or (3) 0.480 mol% $Fe(OAc)_2$. All data points reflect the average of a minimum of three independent reactions and error bars represent the standard deviation from the multiple independent experiments.

Procedure for studying the kinetics of styrene oxidation to benzaldehyde. Under an atmosphere of dry dinitrogen, three 10 mL vials with stir bars were charged

with 7.5 mL (84.6 mmol) benzene, HOPiv (0.960 mol% relative to benzene, 82.9 mg, 0.812 mmol), styrene (0.960 mol% relative to benzene, 93 μL, 0.812 mmol), and either $[(\eta^2\text{-}C_2H_4)_2Rh(\mu\text{-}OAc)]_2$ (0.001 mol% relative to benzene per single Rh atom, 0.18 mg, 0.846 μmol per single Rh atom), $Fe(OAc)_2$ (0.480 mol% relative to benzene, 69.9 mg, 0.406 mmol) or no additive. The vials were inserted into stainless steel reactors, which were subsequently sealed. The reactors were subsequently pressurized with 70 psig of ethylene and 3 atm of dioxygen and heated in an aluminum block on a hot plate at 170 °C. Upon cooling to room temperature, reactors were sampled in air using a long needle. Next, 50 μL aliquots of the reaction mixtures were combined with 50 μL of a 11.1 mM hexamethylbenzene benzene solution and diluted in 0.25 mL of benzene to give an effective hexamethylbenzene concentration of 11.1 mM. The benzene solutions were washed with a saturated aqueous solution of NaOH (1.5 mL) to remove Fe complexes and carboxylic acid, and the organic layer was analyzed by GC-MS.

Discussion on the Optimization of Anaerobic Catalysis. Given the relatively short-lived catalysis observed at aerobic conditions and the observation of benzaldehyde as a significant side product, the turnover frequency, selectivity and longevity of anaerobic catalysis using $Fe^{III}_6(\mu\text{-}OH)_2(\mu_3\text{-}O)_2(\mu\text{-}X)_{12}(HX)_2$ as the oxidant and limiting reagent were studied. $Fe^{III}_6(\mu\text{-}OH)_2(\mu_3\text{-}O)_2(\mu\text{-}X)_{12}(HX)_2$ was obtained by heating $Fe(OAc)_2$ and 2 equiv of HOPiv {relative to $Fe(OAc)_2$} in the presence of 3 atm of dioxygen at 170 °C for two hours prior to the addition of $[(\eta^2\text{-}C_2H_4)_2Rh(\mu\text{-}OAc)]_2$. We studied the effect of $Fe^{III}_6(\mu\text{-}OH)_2(\mu_3\text{-}O)_2(\mu\text{-}X)_{12}(HX)_2$ loading with the goal of maximizing the loading of $Fe^{III}_6(\mu\text{-}OH)_2(\mu_3\text{-}O)_2(\mu\text{-}X)_{12}(HX)_2$ without

negatively impacting the turnover frequency and catalyst longevity. Shown in Figure S29a is a TOs versus time plot at varying $Fe^{III}_6(\mu\text{-}OH)_2(\mu_3\text{-}O)_2(\mu\text{-}X)_{12}(HX)_2$ loadings, which demonstrates the turnover frequency as a function of $Fe^{III}_6(\mu\text{-}OH)_2(\mu_3\text{-}O)_2(\mu\text{-}X)_{12}(HX)_2$ loading. The initial turnover frequency increases from 800 to 1600 equiv of $Fe^{III}_6(\mu\text{-}OH)_2(\mu_3\text{-}O)_2(\mu\text{-}X)_{12}(HX)_2$, and use of 2400 equiv of $Fe^{III}_6(\mu\text{-}OH)_2(\mu_3\text{-}O)_2(\mu\text{-}X)_{12}(HX)_2$ gives a similar rate to 1600 equiv. Increasing the $Fe^{III}_6(\mu\text{-}OH)_2(\mu_3\text{-}O)_2(\mu\text{-}X)_{12}(HX)_2$ loading to 3200 equiv results in both a slower initial rate and apparently shorter-lived catalysis. Figure S29b illustrates that 100% conversion relative to $Fe^{III}_6(\mu\text{-}OH)_2(\mu_3\text{-}O)_2(\mu\text{-}X)_{12}(HX)_2$ is achieved with 800 and 1600 equiv of $Fe^{III}_6(\mu\text{-}OH)_2(\mu_3\text{-}O)_2(\mu\text{-}X)_{12}(HX)_2$, but apparent catalyst deactivation occurs at low conversions with 2400 and 3600 equiv. These findings suggest that high $Fe^{III}_6(\mu\text{-}OH)_2(\mu_3\text{-}O)_2(\mu\text{-}X)_{12}(HX)_2$ loadings promote a catalyst deactivation pathway. 1600 equiv of Fe_6 was taken as optimal, as the initial reaction rate was maximized at these conditions, with quantitative conversion of Fe_6 without evidence for catalyst deactivation achieved.

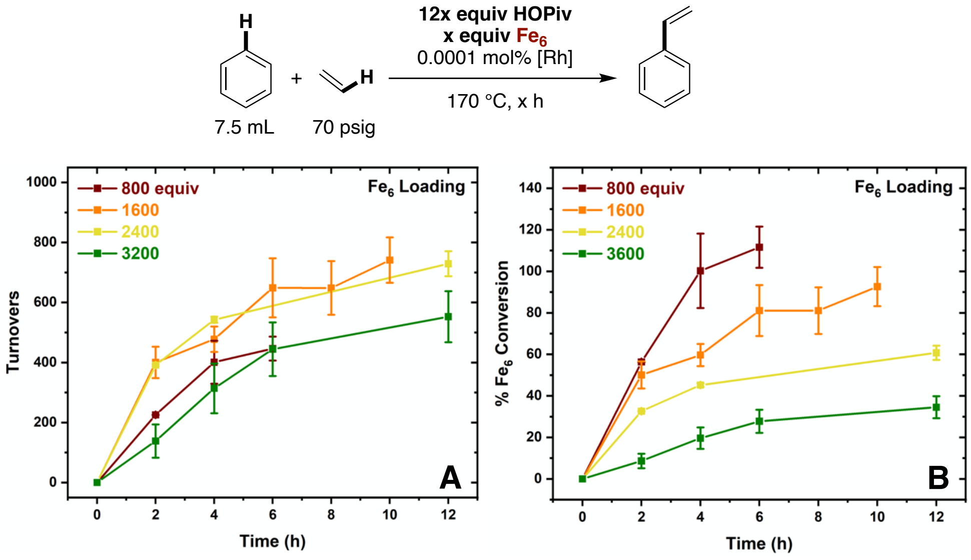

Figure S29. TOs of styrene versus time plots as a function of Fe loading. Reaction conditions: 7.5 mL benzene, 0.0001 mol% (relative to benzene per single Rh atom)

$[(\eta^2\text{-}C_2H_4)_2Rh(\mu\text{-}OAc)]_2$, x equiv Fe_6, 12x equiv HOPiv (relative to Rh), 70 psig ethylene, 80 psig dinitrogen, 170 °C. Fe_6 = $Fe^{III}{}_6(\mu\text{-}OH)_2(\mu_3\text{-}O)_2(\mu\text{-}X)_{12}(HX)_2$ which was prepared *in situ* by heating $Fe(OAc)_2$ and 2 equiv HOPiv (relative to $Fe(OAc)_2$) at 170 °C in 5 mL of benzene under 3 atm of dioxygen for 2 hours. All data points reflect the average of a minimum of three independent reactions and error bars represent the standard deviation from the multiple independent experiments.

The longevity and turnover frequency as a function of catalyst loading was optimized next, with the goal of minimizing the Rh loading while achieving quantitative yields relative to $Fe^{III}{}_6(\mu\text{-}OH)_2(\mu_3\text{-}O)_2(\mu\text{-}X)_{12}(HX)_2$. As shown in Figure S30a, a similar turnover frequency is observed with 0.00005 mol% and 0.0001 mol% loadings of $[(\eta^2\text{-}C_2H_4)_2Rh(\mu\text{-}OAc)]_2$ (relative to benzene per single Rh atom), while a slower initial turnover frequency was observed with a Rh loading of 0.00001 mol%. Figure S30b demonstrates that approximate quantitative yields relative to $Fe^{III}{}_6(\mu\text{-}OH)_2(\mu_3\text{-}O)_2(\mu\text{-}X)_{12}(HX)_2$ are achieved with Rh loadings of 0.0001 and 0.00005 mol%, while apparent deactivation occurs prior to a 100% conversion when a catalyst loading of 0.00001 mol% is used. From the studies of $[(\eta^2\text{-}C_2H_4)_2Rh(\mu\text{-}OAc)]_2$ and $Fe^{III}{}_6(\mu\text{-}OH)_2(\mu_3\text{-}O)_2(\mu\text{-}X)_{12}(HX)_2$ loading, conditions with a 0.00005 mol% loading of $[(\eta^2\text{-}C_2H_4)_2Rh(\mu\text{-}OAc)]_2$ and 3200 equiv (0.160 mol%) of $Fe^{III}{}_6(\mu\text{-}OH)_2(\mu_3\text{-}O)_2(\mu\text{-}X)_{12}(HX)_2$ were taken as optimal (entry shown in orange in Figure S30).

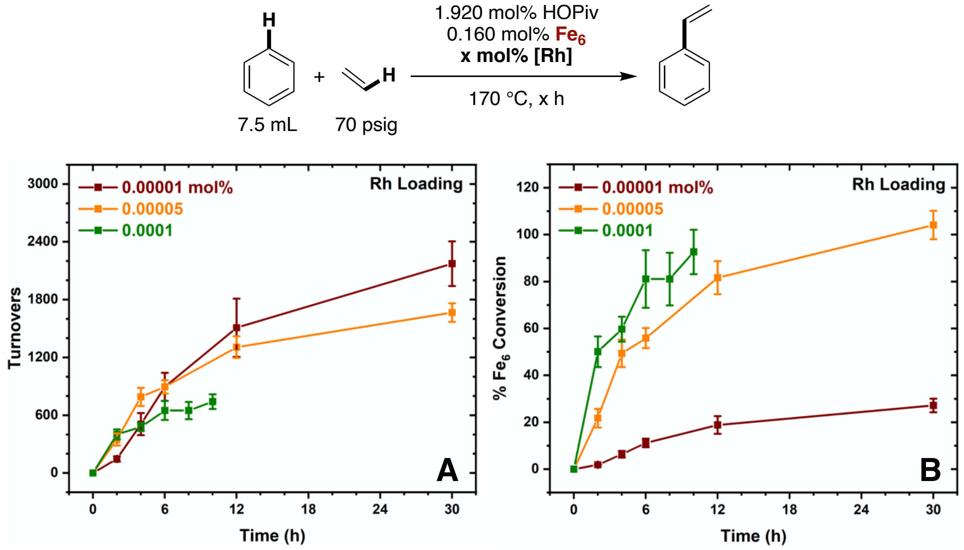

Figure S30. TOs of styrene versus time plots as a function of Rh loading. Reaction conditions: 7.5 mL benzene, x mol% (relative to benzene per single Rh atom) $[(\eta^2\text{-}C_2H_4)_2Rh(\mu\text{-}OAc)]_2$, 0.160 mol% Fe_6, 1.920 mol% HOPiv, 70 psig ethylene, 80 psig dinitrogen, 170 °C. $Fe_6 = Fe^{III}_6(\mu\text{-}OH)_2(\mu_3\text{-}O)_2(\mu\text{-}X)_{12}(HX)_2$ which was prepared *in situ* by heating 0.81 mmol of $Fe(OAc)_2$ and 1.62 mmol of HOPiv at 170 °C in 5 mL of benzene under 3 atm of dioxygen for 2 hours. All data points reflect the average of a minimum of three independent reactions and error bars represent the standard deviation from the multiple independent experiments.

Procedure for anaerobic benzene ethenylation kinetics at varying $Fe^{III}_6(\mu\text{-}OH)_2(\mu_3\text{-}O)_2(\mu\text{-}X)_{12}(HX)_2$ concentration. Under an atmosphere of dry dinitrogen, three 10 mL vials with stir bars were charged with 5 mL (84.6 mmol) benzene, and a 1:2 molar ratio of $Fe(OAc)_2$:HOPiv. For the experiment with 800 equiv of $Fe^{III}_6(\mu\text{-}OH)_2(\mu_3\text{-}O)_2(\mu\text{-}X)_{12}(HX)_2$, 0.406 mmol (69.9 mg) of $Fe(OAc)_2$ was combined with 0.812 mmol (82.9 mg) of HOPiv. The vials were inserted into previously described stainless steel reactors. The reactors were subsequently pressurized with 70 psig of dinitrogen and 45 psig of dioxygen and heated in an aluminum block on a hot plate at 170 °C for two hours. The quantity of $[(\eta^2\text{-}C_2H_4)_2Rh(\mu\text{-}OAc)]_2$ for three reactions (0.054 mg, 0.254 μmol per Rh atom) was added to 7.5 mL of benzene, and 2.5 mL aliquots of this solution were added to each of the three stainless steel reactors.

Dioxygen was removed from the reactors by cycling between dinitrogen pressure and partial vacuum on a high-pressure line, and the reactors were subsequently pressurized with 70 psig of ethylene. The reactors were heated in an aluminum block on a hot plate at 170 °C. Next, 50 μL aliquots of the reaction mixtures were combined with 50 μL of an 11.1 mM benzene solution of hexamethylbenzene in 0.25 mL of benzene to give 1,000 equiv of external standard hexamethylbenzene. The benzene solutions were washed with a saturated aqueous solution of NaOH (1.5 mL) to remove Fe salts and carboxylic acid, and the organic layer was analyzed by GC-MS.

Procedure for anaerobic benzene ethenylation kinetics at varying [(η^2-C_2H_4)$_2$Rh(μ-OAc)]$_2$ concentration. Under an atmosphere of dry dinitrogen, three 10 mL vials with stir bars were charged with 5 mL (84.6 mmol) benzene, $Fe(OAc)_2$ (139.7 mg, 0.812 mmol), and HOPiv (165.7 mg, 1.62 mmol). The vials were inserted into previously described stainless steel reactors. The reactors were subsequently pressurized with 70 psig of dinitrogen and 45 psig of dioxygen and heated in an aluminum block on a hot plate at 170 °C for two hours. The quantity of [(η^2-C_2H_4)$_2$Rh(μ-OAc)]$_2$ for three reactions (0.0254, 0.0508 or 0.254 μmol per Rh atom) was added to 7.5 mL of benzene, and 2.5 mL aliquots of this solution were added to each of the three stainless steel reactors. Dioxygen was removed from the reactors by cycling between dinitrogen pressure and partial vacuum on a high-pressure line, and the reactors were subsequently pressurized with 70 psig of ethylene. The reactors were heated in an aluminum block on a hot plate at 170 °C. Next, 50 μL aliquots of the reaction mixtures were combined with 50 μL of an 11.1 mM benzene solution of

hexamethylbenzene in 0.25 mL of benzene to give 1,000 equiv of external standard hexamethylbenzene. The benzene solutions were washed with a saturated aqueous solution of NaOH (1.5 mL) to remove Fe salts and carboxylic acid, and the organic layer was analyzed by GC-MS.

Procedure for anaerobic benzene ethenylation kinetics at varying reaction temperature. Under an atmosphere of dry dinitrogen, three 10 mL vials with stir bars were charged with 5 mL (84.6 mmol) benzene, $Fe(OAc)_2$ (139.7 mg, 0.812 mmol), and HOPiv (165.7 mg, 1.62 mmol). The vials were inserted into previously described stainless steel reactors. The reactors were subsequently pressurized with 70 psig of dinitrogen and 45 psig of dioxygen and heated in an aluminum block on a hot plate at 170 °C for two hours. The quantity of $[(\eta^2\text{-}C_2H_4)_2Rh(\mu\text{-}OAc)]_2$ for three reactions (0.0276 g, 0.0423 μmol) was added to 7.5 mL of benzene, and 2.5 mL aliquots of this solution were added to each of the three stainless steel reactors to give an Rh loading of 0.00005 mol% relative to benzene per single Rh atom. Dioxygen was removed from the reactors by cycling between dinitrogen pressure and partial vacuum on a high-pressure line, and the reactors were subsequently pressurized with 70 psig of ethylene and 80 psig of dinitrogen. The reactors were heated in an aluminum block on a hot plate at 170, 180, 190 or 200 °C. Next, 50 μL aliquots of the reaction mixtures were combined with 50 μL of an 11.1 mM benzene solution of hexamethylbenzene in 0.25 mL of benzene to give 2,000 equiv of external standard hexamethylbenzene. The benzene solutions were washed with a saturated aqueous solution of NaOH (1.5 mL) to remove Fe salts and carboxylic acid, and the organic layer was analyzed by GC-MS.

Procedure for anaerobic benzene ethenylation kinetics with varying carboxylic acid. Under an atmosphere of dry dinitrogen, three 10 mL vials with stir bars were charged with 5 mL (84.6 mmol) benzene, $Fe(OAc)_2$ (139.7 mg, 0.812 mmol), and HOPiv, HOiBu or HOAc (1.62 mmol). The vials were inserted into previously described stainless steel reactors. The reactors were subsequently pressurized with 70 psig of dinitrogen and 45 psig of dioxygen and heated in an aluminum block on a hot plate at 170 °C for two hours. The quantity of $[(\eta^2\text{-}C_2H_4)_2Rh(\mu\text{-}OAc)]_2$ for three reactions (0.0276 g, 0.0423 μmol) was added to 7.5 mL of benzene, and 2.5 mL aliquots of this solution were added to each of the three stainless steel reactors to give an Rh loading of 0.00005 mol% relative to benzene per single Rh atom. Dioxygen was removed from the reactors by cycling between dinitrogen pressure and partial vacuum on a high-pressure line, and the reactors were subsequently pressurized with 70 psig of ethylene and 80 psig of dinitrogen. The reactors were heated in an aluminum block on a hot plate at 170 °C. Next, 50 μL aliquots of the reaction mixtures were combined with 50 μL of an 11.1 mM benzene solution of hexamethylbenzene in 0.25 mL of benzene to give 2,000 equiv of external standard hexamethylbenzene. The benzene solutions were washed with a saturated aqueous solution of NaOH (1.5 mL) to remove Fe salts and carboxylic acid, and the organic layer was analyzed by GC-MS.

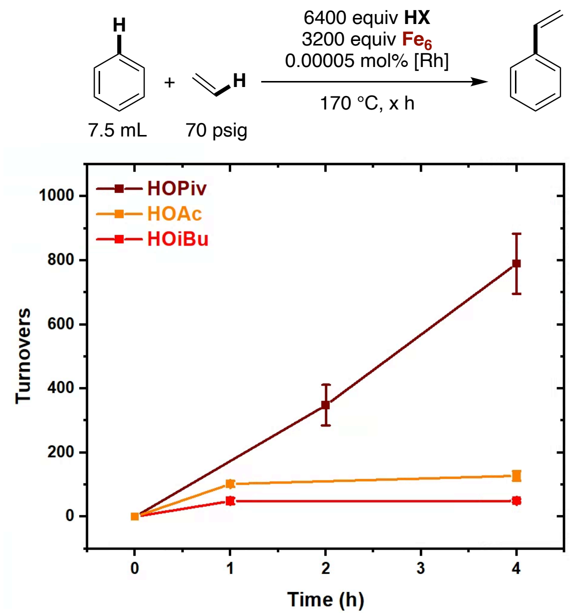

Figure S31. Turnovers versus time plot for anaerobic benzene ethenylation reactions performed with either HOPiv, HOAc or HOiBu additive. Reaction conditions: 7.5 mL benzene, 0.00005 mol% (relative to benzene per single Rh atom) $[(\eta^2\text{-}C_2H_4)_2Rh(\mu\text{-}OAc)]_2$, 3200 equiv Fe_6, 38400 equiv HOPiv, 70 psig ethylene, 80 psig dinitrogen, 170, 200 °C. $Fe_6 = Fe^{III}_6(\mu\text{-}OH)_2(\mu_3\text{-}O)_2(\mu\text{-}X)_{12}(HX)_2$ which was prepared *in situ* by heating 0.81 mmol of $Fe(OAc)_2$ and 1.62 mmol of HOPiv, HOAc or HOiBu at 170 °C in 5 mL of benzene under 3 atm of dioxygen for 2 hours. All data points reflect the average of a minimum of three independent reactions and error bars represent the standard deviation from the multiple independent experiments.

Procedure for anaerobic benzene ethenylation with a separate dioxygen-reoxidation step. Under an atmosphere of dry dinitrogen, three 10 mL vials with stir bars were charged with 5 mL (84.6 mmol) benzene, $Fe(OAc)_2$ (139.7 mg, 0.812 mmol), and HOPiv (165.7 mg, 1.62 mmol). The vials were inserted into previously described stainless steel reactors. The reactors were subsequently pressurized with 70 psig of dinitrogen and 45 psig of dioxygen and heated in an aluminum block on a hot plate at 170 °C for two hours. The quantity of $[(\eta^2\text{-}C_2H_4)_2Rh(\mu\text{-}OAc)]_2$ for three reactions (0.0276 g, 0.0423 μmol) was added to 7.5 mL of benzene, and 2.5 mL aliquots of this solution was added to each of the three stainless steel reactors to give an Rh loading of

0.00005 mol% relative to benzene per single Rh atom. Dioxygen was removed from the reactors by cycling between dinitrogen pressure and partial vacuum on a high-pressure line, and the reactors were subsequently pressurized with 70 psig of ethylene and 80 psig of dinitrogen. The reactors were heated in an aluminum block on a hot plate at 190 °C for four-hour intervals. Next, 50 μL aliquots of the reaction mixtures were combined with 50 μL of an 11.1 mM benzene solution of hexamethylbenzene in 0.25 mL of benzene to give 2,000 equiv of external standard hexamethylbenzene. The benzene solutions were washed with a saturated aqueous solution of NaOH (1.5 mL) to remove Fe salts and carboxylic acid, and the organic layer was analyzed by GC-MS. After each sampling, the reactors were pressurized with 70 psig of dinitrogen and 45 psig of dioxygen and heated at 100 °C for 18 hours. Upon re-oxidation, the reaction mixtures were depressurized and subsequently cycled between ethylene pressure and partial vacuum to remove dioxygen, and pressurized with 70 psig of ethylene and 80 psig of dinitrogen.

Table S3. DFT Energies for hexanuclear Fe species investigated in Figure 6 of manuscript. The number in the species column denotes the spin multiplicity.

Species	H	G	S_{trans}	S_{rot}	S_{vib}	S_{elec}
OO_31	-3784.54664	-3784.65453	46.887	39.591	177.008	6.824
OOH_30	-3785.15214	-3785.26409	46.889	39.801	185.519	6.759
O_OH_30	-3785.16954	-3785.27875	46.889	39.788	179.751	6.759
OH_OH_31	-3785.84529	-3785.95566	46.892	39.839	182.109	6.824
OH_2_OH_30	-3786.44244	-3786.55690	46.895	39.675	190.859	6.759
OH_2_OH_2_29	-3787.05463	-3787.17577	46.897	39.719	204.974	6.692
29_(2 H_2O Removed)	-3634.1436	-3634.2566	46.799	39.465	188.018	6.692
30_(H_2O Removed)	-3709.99380	-3710.1066	46.846	39.532	187.528	6.759
